# Efficacy and safety of varenicline and bupropion, in combination and alone, for alcohol use disorder: a randomized, double-blind, placebo-controlled multicentre trial

**DOI:** 10.1016/j.lanepe.2025.101310

**Published:** 2025-05-13

**Authors:** Bo Söderpalm, Helga Lidö, Johan Franck, Anders Håkansson, Daniel Lindqvist, Markus Heilig, Joar Guterstam, Markus Samuelson, Barbro Askerup, Cecilia Wallmark-Nilsson, Andrea de Bejczy

**Affiliations:** a Addiction Biology Unit, Psychiatry and Neurochemistry Section, Institute of Neuroscience and Physiology, Sahlgrenska Academy, University of Gothenburg, Sweden & Department of Addiction and Dependency, Sahlgrenska University Hospital, Gothenburg, Sweden; b Department of Clinical Neuroscience, Centre for Psychiatry Research, Karolinska Institute & Stockholm Health Care Services, Stockholm County Council, Stockholm, Sweden; c Lund University, Faculty of Medicine, Department of Clinical Sciences Lund, Psychiatry, Lund, Sweden & Office for Psychiatry and Habilitation, Malmö Addiction Centre, Region Skåne, Malmö, Sweden; d Unit for Biological and Precision Psychiatry, Department of Clinical Sciences Lund, Lund University, Sweden and Office for Psychiatry and Habilitation, Psychiatry Research Skåne, Region Skåne, Lund, Sweden; e Centre for Social and Affective Neuroscience, BKV, Linköping University, Sweden; f Malmö Addiction Centre, Bokgatan 16, Malmö, Sweden

**Keywords:** AUD, Bupropion, Combination treatment, RCT, Varenicline

## Abstract

**Background:**

Alcohol use disorder (AUD) is associated with an enormous burden of disease and cost to society. The dopamine deficiency hypothesis posits that negative reinforcement generated by a low brain dopamine state drives ethanol intake. Here, we evaluated the efficacy and safety of combined administration of two dopamine-enhancing drugs, varenicline (a partial nicotinic acetylcholine receptor agonist) and bupropion (a weak dopamine-reuptake inhibitor) on alcohol intake in AUD.

**Methods:**

Participants aged 25–70 years with moderate-to-severe AUD (defined as ≥4/11 Diagnostic and Statistical Manual of Mental Disorders [DSM]-5 criteria) were enrolled in this randomized, double-blind, placebo-controlled trial, done at four outpatient clinics in Sweden. Participants were randomly assigned (block size 8) 1:1:1:1 to Placebo + Placebo, Varenicline + Bupropion, Varenicline + Placebo, or Placebo + Bupropion. After a 1-week titration period, Varenicline was taken as 1 mg orally twice per day and bupropion as 150 mg orally twice per day for 12 weeks. Participants, investigators, and all study personnel were unaware of treatment allocation. The two primary outcomes were phosphatidylethanol in blood (B-PEth) and self-reported percentage heavy drinking days (%HDD), assessed over a steady state 10-week-period (from start of week 2 to end of week 11). Modified intention-to-treat (mITT) and per protocol analyses (PP) were performed using a sequential hierarchical statistical method. This registered study (EudraCT 2018–000048-24; clinicaltrials.govNCT04167306) is completed.

**Findings:**

Between March 4, 2019, and December 14, 2022, 384 participants were randomly assigned: Placebo + Placebo = 97, Varenicline + Bupropion = 100, Varenicline + Placebo = 96, Placebo + Bupropion = 91. 72% participants were male (277/384) and 28% female (107/384), median age 57 (13) years. In the mITT analyses, Varenicline + Bupropion reduced B-PEth (Cohen's d [d] = 0·39, p = 0·004) and %HDD (d = 0·31, p = 0·008) vs Placebo + Placebo. Varenicline + Placebo also reduced B-PEth (d = 0·30, p = 0·005) and %HDD (d = 0·36, p = 0·023) vs Placebo + Placebo. For both primary endpoints, differences between the Varenicline + Bupropion and Varenicline + Placebo groups were not statistically significant (B-PEth: d = 0·022, p = 0·97, %HDD: d = 0·027, p = 0·76), precluding further comparisons according to the statistical hierarchy. In PP analyses, both primary outcomes were reduced with Varenicline + Bupropion (d = 0·43 [B-PEth]; d = 0·41 [%HDD]) and Varenicline + Placebo (d = 0·29 [B-PEth]; d = 0·34 [%HDD]) compared with Placebo + Placebo. Nausea, the only safety concern, was more common in the Varenicline + Placebo group than in the Placebo + Placebo group (49/96 vs 11/97, p < 0·0001) and of longer median duration (45 (70) vs 10 (14·5) days, p = 0·001). Nausea incidence was lower in the Varenicline + Bupropion group vs Varenicline + Placebo (36/100 vs 49/96, p = 0·048) and of shorter median duration (16·5 (39·3) vs 45 (70) days, p = 0·010).

**Interpretation:**

Two brain dopamine elevating treatments (Varenicline + Bupropion; Varenicline + Placebo) reduce alcohol consumption compared with placebo alone. Effect sizes were largest when Varenicline and Bupropion were combined and compliance was high (PP-population). Bupropion reduced Varenicline-induced nausea. Varenicline + Bupropion or other mild dopamine enhancers should be further explored for treatment of AUD.

**Funding:**

This study was funded primarily by the 10.13039/501100004359Swedish Research Council.

**Research in context**

**Evidence before this study**

Animal experiments and brain imaging studies in alcohol use disorder (AUD) indicate that alcohol intake may be driven by a low dopamine state. However, this hypothesis has not been tested using placebo-controlled, clinical trials that aim to reduce alcohol intake by elevating brain dopamine levels with pharmacological agents. This is especially true for unselected AUD populations and using objective alcohol biomarkers with high sensitivity and specificity, such as phosphatidylethanol in blood (B-PEth), as a primary outcome measure. We searched PubMed using English search terms (“clinical trial” OR “randomized controlled trial”) AND (“alcohol dependence” OR alcoholism) AND (varenicline OR bupropion OR amphetamine OR methylphenidate OR l-DOPA OR selegeline) for studies published before 2018 (when planning this study) and similarly for studies published in 2018–Aug 6, 2024 (when performing the study and writing this report). We determined whether studies were performed in unselected AUD patients or in a subgroup, and whether an objective marker of alcohol intake with high sensitivity and specificity was used as a primary outcome variable. Our initial search revealed no studies on bupropion plus varenicline, bupropion, methylphenidate, amphetamine, or selegeline. We found only two, early, comparatively small, negative studies on l-DOPA, one pilot study and two phase 2 studies on varenicline with conflicting results as regards its effect on the primary outcome that nevertheless provided some support for our hypothesis. Our second search identified two positive studies on varenicline in a subpopulation of people with AUD (those with AUD with heavy smoking) and one study on varenicline plus naltrexone in the same subpopulation. None of the published studies, either before or after 2018, used an objective biomarker with high sensitivity and specificity, as a primary outcome variable.

**Added value of this study**

This randomized and controlled trial has demonstrated, for the first time, that combined administration of varenicline and bupropion, two drugs with complementary dopamine elevating actions, robustly and safely reduces B-PEth and percentage heavy drinking days (two primary endpoints), as well as total alcohol consumption (g/day; secondary endpoint), compared with placebo. Effect sizes were around 0·4 (Cohen's d), albeit larger in participants with the highest compliance. Notably, bupropion reduced varenicline-induced nausea, a common side effect of varenicline. This study confirms animal data, and is the first to translate this beneficial effect of combined varenicline and bupropion on alcohol intake to humans. Furthermore, this is also the first study to use B-PEth, a highly specific and sensitive objective biomarker for alcohol consumption, as a primary outcome variable in a randomized controlled trial on AUD.

**Implications of all the available evidence**

This study provides pharmacological support in humans for the dopamine deficiency hypothesis of AUD and suggests that a combination of varenicline and bupropion is a well-tolerated, safe and efficacious treatment option for AUD. This is important since AUD poses a significant burden globally on healthcare and social-care resources and not least on the individuals affected by AUD. The reduction of nausea of a bupropion/varenicline combination approach, compared with varenicline alone, may also be an advantage when used for smoking cessation.

## Introduction

Approximately 100 million people worldwide meet the criteria for alcohol use disorder (AUD), with 3 million deaths per year attributable to alcohol intake and a life-span shortened by 20–35 years in individuals with the highest drinking levels.^1^ Alcohol consumption imposes a tremendous burden on healthcare and social-care systems. The annual cost to Sweden alone is approx. 10 billion USD.^2^ Since the life-time risk of dying from alcohol-related causes increases exponentially with alcohol consumption, the risk of death in individuals at the highest levels of use can be profoundly lowered not only by abstinence, but also by reduced alcohol intake.^3^ Therefore, harm reduction has become the new aim of treating AUD in addition to abstinence—especially as many individuals are not motivated by abstinence, but can accept a low-risk drinking level goal.^4^

Disulfiram, acamprosate, and naltrexone are approved for the treatment of AUD by the US Food and Drug Administration and the European Medicines Agency (EMA), with nalmefene additionally being approved by the EMA.^5^ Disulfiram, an aldehyde dehydrogenase inhibitor, is limited to individuals who accept abstinence as a treatment goal due to the adverse effects of acetaldehyde accumulation upon drinking, and its efficacy is only supported with supervised administration.^6^ The functional glutamate antagonist acamprosate and the mu-opioid receptor antagonists naltrexone and nalmefene can be used to promote abstinence and to achieve harm reduction goals, but effect sizes are small (Cohen's d [d] = 0·2)^7^ and clinical uptake is minimal. A lack of effective therapies contributes to a treatment gap of 90%^8^ that could be narrowed using treatments with larger effect sizes.

Notably, none of the available treatments was originally developed based on theories of AUD neurobiology. The mesolimbic dopamine system has long been implicated in the rewarding effects of ethanol, and prolonged exposure to ethanol appears to down-regulate this system.^9^ Reduced dopamine neurotransmission has been associated with increased alcohol intake, craving, and ethanol-cue reactivity in both animal^10^ and human studies.^11^ Furthermore, dopamine neurotransmission is reduced both pre- and post-synaptically in AUD.^12^ A compromised dopamine system, due to chronic alcohol use, genetic factors, or both, has been hypothesized to attenuate the value of natural rewards and/or to produce anhedonia that by negative reinforcement increases incentive for alcohol use,^8^ as supported by the worsening of AUD by the dopamine D_2_-receptor antagonist, flupentixole.^13^

The smoking cessation agent Varenicline releases dopamine via a partial agonistic effect at brain nicotinic acetylcholine receptors (nAChRs).^14^ Varenicline reduces alcohol intake in people with AUD with effect sizes (d) of approximately 0·3 in randomized controlled trials (RCTs) using subjectively reported outcomes^15^ or the specific biomarker of alcohol use, phosphatidylethanol in blood (B-PEth).^16^^,^^17^ We have shown that the weak dopamine-reuptake inhibitor Bupropion enhances the effect of Varenicline in a rat model of dopamine output in the nucleus accumbens and reduces the alcohol deprivation effect in rat,^18^ a model with predictive value of clinical responses.^19^ Additionally, we have reported that B-PEth detected an effect of Varenicline to reduce drinking that was not detected by self-report measures.^16^

The main aim of this RCT was to assess the efficacy and safety of Varenicline and Bupropion in combination on alcohol consumption vs Placebo in participants with moderate-to-severe AUD. Secondary aims were to investigate whether earlier findings with Varenicline could be replicated, and whether Bupropion could also have an effect. Varenicline and Bupropion have complementary dopamine-enhancing mechanisms so by examining whether Varenicline + Bupropion reduces alcohol intake in participants with moderate-to-severe AUD significantly more than Placebo + Placebo and with larger effect sizes than Varenicline or Bupropion alone, we were also able to address pharmacologically the dopamine deficiency hypothesis of AUD. For efficacy, we used two primary outcomes, i.e., one objective measure, B-PEth, and one subjective measure that covers the aspect of intensive drinking occasions and is accepted by regulatory authorities (percentage of heavy drinking days, %HDD; one HDD = >70 g/day and >56 g/day for men and women, respectively). Secondary outcomes included total alcohol consumption (g/day) measured by the timeline follow-back procedure (TLFB) for self-reported alcohol consumption.^20^

## Methods

### Study design

The detailed methodology for this Phase 2 study has been published.^21^ The study is registered with EudraCT 2018–000048-24 (approved December 28, 2018) and clinicaltrials.gov identifier: NCT04167306.

This is a randomized, double-blind, placebo-controlled, 13-week, multicentre clinical trial performed at four outpatient clinics in Sweden, with four parallel arms designed to evaluate the efficacy of two substances, Varenicline and Bupropion, in combination and alone vs Placebo on alcohol consumption in participants with moderate to severe AUD. Treatment comprised one week of titration (Varenicline from 0.5 to 1 mg orally twice daily**,** Bupropion SR from 150 mg orally once to twice daily or corresponding Placebo capsules) plus 12 weeks of full-dose treatment (Varenicline 2 mg daily, Bupropion SR 300 mg daily). No dose adjustment was allowed. Two primary efficacy endpoints were applied: (i) alcohol consumption as measured by B-PEth (μmol/L), and (ii) subjective alcohol consumption as measured by %HDD using the timeline follow-back (TLFB) procedure and one HDD defined as ≥5 units/day for men and ≥4 units/day for women, with 1 unit defined as 14 g of pure alcohol.

### Participants

Potential participants who had responded to media advertisements (newspapers, radio or TV) were pre-screened for eligibility by telephone or on site. Eligible individuals were required to be aged 25–70 years at screening, be diagnosed with moderate-to-severe AUD (≥4/11 Diagnostic and Statistical Manual of Mental Disorders [DSM]-5 criteria), to provide signed informed consent, and have a B-PEth level of ≥0·5 μmol/L at screening and high alcohol consumption during the 3 months prior to screening (i.e., ≥2 heavy drinking days [HDD] per week during a typical week). Key exclusion criteria included abstinence between screening and randomisation visits, receiving alcohol withdrawal treatment within 30 days of study initiation, or any pharmacological/non-pharmacological treatment affecting alcohol consumption 3 months pre-screening or during the study. Individuals with significant abnormal laboratory or clinical values, any current clinically significant psychiatric/medical disorders, medication(s) that could compromise safety or affect study outcomes, or a history of delirium tremens/seizures or need for alcohol withdrawal treatment were excluded.

### Procedures

The study period comprised 9 visits (Visit 1, screening to assess eligibility) to the clinic for monitoring and evaluation of participants and blood sample collection. Data collected included TLFB, demographics, alcohol-specific factors, nicotine-specific factors, AUD diagnosis, medical history, routine physical examination, and vital signs. All assessments, timelines, scales and questionnaires have been described previously.^21^ Data were recorded (coded) in the electronic Case Report File provided by Medicase AB (Sweden) and handled according to the Data Management Plan (Appendix).

The study was conducted according to the protocol, the Declaration of Helsinki, GCP principles, and all applicable national and EU regulatory requirements with ethical approval granted by the Swedish Ethical Review Authority (D.nr. 431-18, 2018-06-18).

### Randomisation and masking

Participants were randomly assigned 1:1:1:1 to Placebo + Placebo, Varenicline + Bupropion, Varenicline + Placebo, or Placebo + Bupropion. Apoteket Produktion & Laboratorier (APL, Sweden) manufactured blinded study medication with encapsulated, commercially available varenicline tartrate (Pfizer Innovations, USA/Apotex, Canada), Bupropion Sandoz SR (slow-release formulation; Sandoz AS, Switzerland), and placebo. APL also performed block randomisation (block size 8) and allocated randomised study drugs to study sites. APL generated and kept the randomisation code. Eligible participants were randomised consecutively. Treatment was blinded to participants, investigators, and all study personnel. The blinding code was only to be broken in medical emergencies where treatment knowledge was necessary. In this study, the code was only broken after database lock and the statistical analysis was performed blinded with treatment groups coded.

### Outcomes

The efficacy of Varenicline and Bupropion in combination and alone compared to Placebo (primary outcome) was evaluated using two primary endpoints of alcohol consumption: B-PEth (μmol/L) and %HDD (=HDD share) over steady state treatment analysis period weeks 2–11. Statistically significant reductions of both B-PEth and %HDD by the combination of Varenicline + Bupropion compared to Placebo alone would constitute a successful outcome of the study.

The main secondary outcome reported here was total consumption of alcohol in mean grams of alcohol per day (g/day) with additional secondary outcomes of percentage of abstinent days and alcohol craving measured by a visual analogue scale. The study protocol included also GGT, CDT, and AUDIT as secondary outcomes. Before analyses it was decided to limit the secondary outcomes to such collected at every visit, i.e., those retrieved by TLFB and alcohol craving. GGT and CDT were collected at every visit but are inferior to the primary outcome B-PEth^17^ and were judged not to add more information in this context. They will however be reported in a planned follow-up paper on comparisons of the three biomarkers in relation to self-reported alcohol consumption by the TLFB method.

The study was monitored and independently audited by Hellgren GCP Consulting. Safety was monitored using blood pressure measurement and clinical and laboratory parameters (including levels of aspartate aminotransferase, alanine aminotransferase, prothrombin complex, creatinine, glucose, Na^+^/K^+^, haemoglobin, high-sensitive C-reactive protein, and human chorionic gonadotropin for fertile women, as well as the leukocyte plasma count, thrombocyte particle concentration, and urine toxicology), and the Montgomery-Asberg Depression Scale and Drug Disorder Identification Test self-reporting scales for depression and drug abuse. Adverse events were coded using the Medical Dictionary for Regulatory Activities and assessed and authorized by a responsible medical physician. All serious adverse events were reported.

### Statistical analyses

#### Populations

The mITT cohort included all participants from the ITT population who had completed the screening visit and the randomisation visit and had reported that they had taken at least one dose of the randomised Investigational Medical Product (IMP). The PP cohorts comprised all participants from the ITT population who had taken the IMP at least 80% of the planned number of days of the active treatment period (as determined by pill count) and who had completed the TLFB and provided successful B-PEth analyses at the screening visit, Day 0, Day 21, Day 49, and Day 77.

### Sample size calculation

The power analysis was based on two independent primary hypotheses: (i) B-PEth differed significantly between Varenicline and Bupropion in combination and alone vs Placebo; and, (ii) the %HDD differed significantly between Varenicline and Bupropion in combination and alone vs Placebo.

As regards B-PEth, it was assumed that ordinary t-tests based on normal distribution, with α = 0·025, 1-β = 0·80 and using a standard deviation of 0·8 [based on previous results^16^] could be used. Recruiting 320 individuals who were randomised to four groups of 80 participants per group resulted in a detectable effect of 0·35 μmol/L and a Cohen's d of 0·44.

As regards HDD, it was assumed that ordinary t-tests based on normal distribution, with α = 0·025, 1-β = 0·80 and using a standard deviation of maximum 0·35 [based on previous results^15^] could be used. Recruiting 360 individuals who were randomised to four groups of 90 participants per group resulted in a detectable effect of 15% and a Cohen's d of 0·43. Hence, a sample size of approximately 360 patients was required for randomisation.

Power calculations were based on assumptions that allowed detection of lower effect sizes than originally hypothesized in the study protocol, in order to decrease the risk for type 2 errors.

Based on prior studies^16^ with similar design and outcome, the drop-out rate is estimated to approx. 20% between screening and randomisation and approx. 6.5% from randomisation to participants included in mITT. To balance the drop-out, 380 participants are estimated for randomisation to reach 360 participants in the mITT.

All statistical analyses followed the statistical analysis plan (Appendix). The two primary efficacy outcomes, B-PEth and %HDD, were calculated separately as the change in alcohol consumption between baseline measurement (screening) and the mean of the reported measurements, over a 10-week steady state treatment period (visits 4 to 8, i.e., data from the beginning of week 2 until the end of week 11). Visit 9 was predetermined to be omitted from the statistical analyses based on the assumption that data collected towards the end of clinical trials are more unreliable due, for example, to drop-outs, reduced compliance, and lack of motivation.^16^

Missing data were assessed in a blind data review. Available data were used for analysing participants who did not follow the precise treatment regime. Mean values were calculated using available data and no imputations were conducted. Pairwise comparisons between treatment arms were then tested via a sequential test procedure, in a hierarchical method, with an overall significance level of 5% (see Supplement). When a test was significant, the procedure continued to the next hypothesis, and the test proceeded until the first non-significant outcome. Hypotheses were as follows (in hierarchical order): Varenicline + Bupropion vs Placebo + Placebo; Varenicline + Placebo vs Placebo + Placebo; Varenicline + Placebo vs Varenicline + Bupropion; Placebo + Bupropion vs Varenicline + Bupropion; and, Placebo + Bupropion vs Placebo + Placebo. The hierarchical order was based on hypotheses generated in a preceding study in rats^18^ and on available clinical data.^15^^,^^16^ In each step, the within-patient difference for B-PEth and %HDD was analysed using a linear regression model blocked for gender and baseline value of B-PEth and %HDD, respectively. Correction for multiple comparisons was inherent to the statistical method used for the primary outcomes, with a total type 1 error rate at 5% for each primary endpoint. Additionally, the Cohen's d (d) effect size was estimated in each test.

Secondary outcomes were limited to those retrieved at all visits by TLFB and by a visual analogue scale (craving) and are reported as total consumption of alcohol (g/day), % abstinent days (=abstinent days share), and alcohol craving, and were calculated as the within-patient difference between baseline and the mean of visits 4–8. All comparisons between treatment arms were tested, with an individual significance level of 5%, with and without multiple testing adjustments (Holm-Bonferroni). Descriptive statistics are shown per visit. Nicotine (QF) was recorded only at baseline.

The prevalence of nausea was analysed using chi-square tests and the duration of nausea (days) was analysed using Wilcoxon rank sum tests.

### Role of the funding source

The funders of the study had no role in its design, data collection, analysis, or interpretation, the writing of this report, or in the decision to submit the paper for publication.

## Results

This study was performed between March 4, 2019 and December 14, 2022.

**Fig. 1:**
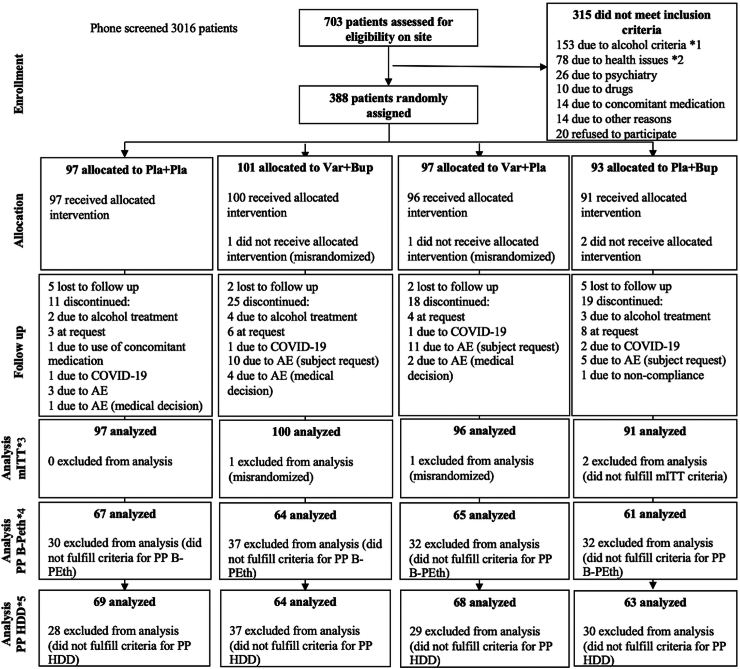
**Study flow of COMB participants (CONSORT) and analysis groups**. Figure shows the final cohort included in the mITT analyses (i.e., all participants who completed the screening and randomisation visits and that reported having taken at least one dose of the randomised study drug). All mITT participants were analysed according to their randomised study drug. The PP analysis set constituted all participants from the mITT population considered to be a “completer” defined as a participant who had taken the study drug at least 80% of the planned number of days of the active treatment period and provided successful outcome measures of B-PEth and/or TFLB at screening and visits 2, 4, 6 and 8. All inclusion and exclusion criteria have been reported previously.^21^ ∗1 including B-PEth levels <0·5, abstinence between screening and randomisation, need of alcohol withdrawal treatment, and concomitant alcohol treatment; ∗2 including, for example, blood pressure >180/110 mmHg and high liver enzymes/physical signs of liver failure. ∗3 mITT criteria; completed screening and randomisation visit and reported having taken at least one full dose of study drug. ∗4 PP B-PEth criteria; reported having taken at least 80% of the study drug from Visit 2 through Visit 8 and provided successful B-PEth sampling from Visit 4, Visit 5 or 6, and Visit 7 or 8. ∗5 PP HDD criteria; reported having taken at least 80% of the study drug from Visit 2 through Visit 8 and provided successful TLFB data from Visit 4, Visit 5 or 6, and Visit 7 or 8. AE, adverse event; B-PEth, phosphatidylethanol in blood; Bup, bupropion; COVID-19, infection with SARS-Cov·2; HDD, heavy drinking days; mITT, modified intention-to-treat; PP, per-protocol; Pla, placebo; TFLB, timeline follow-back procedure; Var, varenicline.

Of the individuals who had responded to media advertisements, 3016 were pre-screened for eligibility by telephone and 703 on site. The “first patient in” was reported on March 4, 2019 and the “last patient out” on December 14, 2022. Participants were distributed across the following sites: Gothenburg (n = 247), Region Skåne (n = 62), Stockholm (n = 61), and Linköping (n = 18). A total of 388 consenting, eligible participants were randomised to one of the four study arms and of these, N = 384 were included in the mITT analyses. One participant was incorrectly randomized because two B-PEth analysis results were confused, and one individual was randomized erroneously in the system due to a data management error. Both of these misrandomisations were discovered before the participants received an IMP. Two other participants did not meet the mITT criteria. None of these four participants entered any analyses. Fig. 1 shows the final cohorts included in the mITT and PP analyses (Fig. 1).

The study was performed during an ongoing pandemic (COVID-19); consequently, the PP-population was smaller than expected.

Of all participants enrolled, 72% were men and 28% women, similar to the relative prevalence of AUD. The proportion of nicotine users was 49%. Demographic characteristics of the mITT population are shown in Table 1, and those of the PP B-PEth and HDD populations in Tables S1 and S2 in the Appendix.

**Table 1:** Demographics of the modified intention-to-treat population.

	Pla + Pla (n = 97)	Var + Bup (n = 100)	Var + Pla (n = 96)	Pla + Bup (n = 91)	Total (n = 384)
**Baseline B-PEth**					
Mean (SD)	1·2 (0·55)	1·2 (0·66)	1·2 (0·76)	1·3 (0·62)	1·2 (0·65)
Median [Min, Max]	1·1 [0·50, 2·6]	1·0 [0·50, 4·1]	0·99 [0·52, 5·0]	1·2 [0·50, 3·1]	1·1 [0·50, 5·0]
**Baseline proportion of HDD**					
Mean (SD)	0·74 (0·28)	0·72 (0·30)	0·76 (0·25)	0·75 (0·26)	0·74 (0·27)
Median [Min, Max]	0·86 [0, 1·0]	0·86 [0, 1·0]	0·86 [0·14, 1·0]	0·86 [0, 1·0]	0·86 [0, 1·0]
**Sex, n (%)**^a^					
Male	63 (64·9%)	72 (72·0%)	69 (71·9%)	73 (80·2%)	277 (72·1%)
Female	34 (35·1%)	28 (28·0%)	27 (28·1%)	18 (19·8%)	107 (27·9%)
**Age (years)**					
Mean (SD)	56 (9·9)	56 (8·3)	56 (9·7)	56 (8·9)	56 (9·2)
Median [Min, Max]	58 [31, 70]	57 [33, 69]	56 [32, 70]	57 [31, 70]	57 [31, 70]
**Marital status, n (%)**					
Single	16 (16·7%)	18 (18·0%)	14 (14·6%)	15 (16·5%)	63 (16·4%)
Married	47 (49·0%)	45 (45·0%)	50 (52·1%)	42 (46·2%)	184 (48·0%)
Partner, cohabiting	12 (12·5%)	17 (17·0%)	15 (15·6%)	16 (17·6%)	60 (15·7%)
Partner, non-cohabiting	6 (6·3%)	10 (10·0%)	7 (7·3%)	8 (8·8%)	31 (8·1%)
Divorced	12 (12·5%)	6 (6·0%)	9 (9·4%)	7 (7·7%)	34 (8·9%)
Separated	1 (1·0%)	3 (3·0%)	1 (1·0%)	2 (2·2%)	7 (1·8%)
Widowed	2 (2·1%)	1 (1·0%)	0 (0%)	1 (1·1%)	4 (1·0%)
**Education, n (%)**					
Preparatory high school	37 (38·5%)	51 (51·0%)	38 (39·6%)	41 (45·1%)	167 (43·6%)
High school	13 (13·5%)	5 (5·0%)	5 (5·2%)	9 (9·9%)	32 (8·4%)
Higher education ≤3 years	21 (21·9%)	21 (21·0%)	32 (33·3%)	24 (26·4%)	98 (25·6%)
Higher education >3 years	24 (25·0%)	23 (23·0%)	21 (21·9%)	17 (18·7%)	85 (22·2%)
**Weight (kg)**					
Mean (SD)	85 (16)	91 (16)	86 (17)	86 (18)	87 (17)
Median [Min, Max]	84 [52, 120]	90 [51, 130]	87 [50, 140]	85 [53, 130]	86 [50, 140]
**Age at alcohol debut (years)**					
Mean (SD)	16 (5·3)	15 (3·0)	15 (4·1)	15 (2·7)	16 (3·9)
Median [Min, Max]	15 [7, 48]	15 [12, 35]	15 [7, 40]	15 [11, 30]	15 [7, 48]
**Heredity for alcohol problems, n (%)**					
Yes	16 (16·7%)	25 (25·0%)	15 (15·6%)	17 (18·7%)	73 (19·1%)
No	70 (72·9%)	73 (73·0%)	70 (72·9%)	69 (75·8%)	282 (73·6%)
Don't know	10 (10·4%)	2 (2·0%)	11 (11·5%)	5 (5·5%)	28 (7·3%)
**Nicotine daily, n (%)**					
Yes	49 (51·0%)	48 (48·0%)	41 (43·0%)	50 (54·0%)	188 (49·0%)
No	48 (49·0%)	52 (52·0%)	55 (57·0%)	41 (45·0%)	196 (51·0%)

n = 2 missing for education status as they had been classified as “other”. B-PEth, phosphatidylethanol in blood; Bup, bupropion; HDD, heavy drinking days; Pla, placebo; SD, standard deviation; Var, varenicline.

a As defined by subject. Footnote: ethnicity was not registered in this study (see limitations in Discussion).

**Fig. 2:**
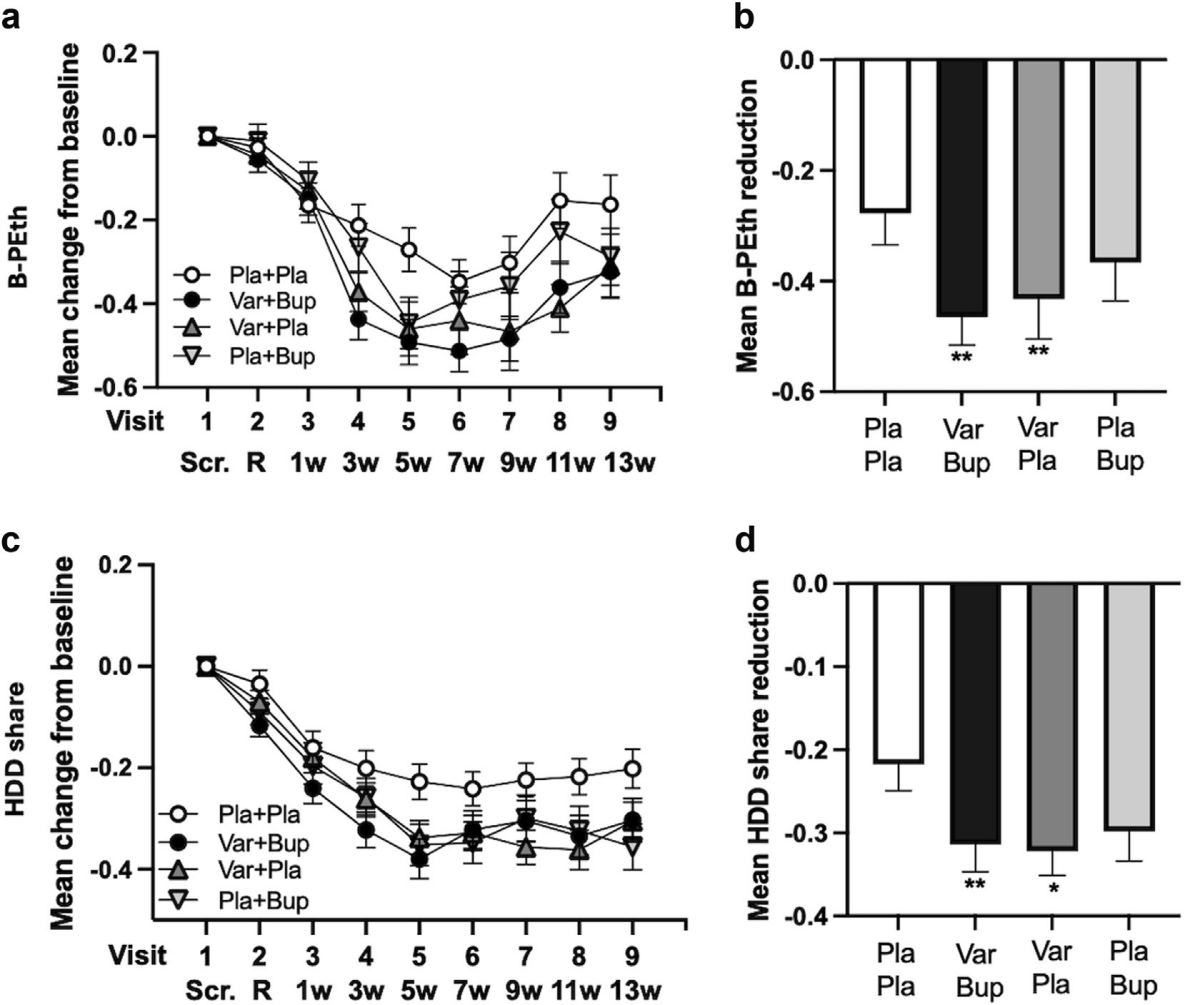
**Mean change from baseline (±SEM) of primary outcome variables B-PEth and HDD share according to the modified intention-to-treat analysis**. a (B-PEth) and c (HDD share) show the mean change at the respective visit (±SEM). b (B-PEth) and d (HDD share) show the mean reduction (±SEM) over visits 4–8, the pre-determined, steady-state period over which statistics were performed. Significance levels and effect sizes (Cohen's d) are presented in Tables 2 and in heat maps (Figure S3, Appendix). B-PEth, phosphatidylethanol in blood; Bup, bupropion; HDD, heavy drinking days; Pla, placebo; SEM, standard error mean; Var, varenicline; Scr., screening day; R, randomisation day; w, week. ∗p = 0·023 vs Pla + Pla, ∗∗p = 0·004 vs Pla + Pla for both Var + Bup and Var + Pla regarding B-PEth. ∗∗p = 0·008 vs Pla + Pla regarding HDD share.

In the mITT cohort, B-PEth levels were reduced in all groups from Visit 2 through Visit 7 (Fig. 2a). At visits 8 and 9, this reduction was largely maintained in the Varenicline + Bupropion and Varenicline + Placebo groups, but had partly returned towards baseline levels in the Placebo + Placebo and Placebo + Bupropion groups. In the mITT analysis of the predetermined, steady-state analysis period (data retrieved from Visit 4 through Visit 8) (Table 2), B-PEth was significantly reduced in the Varenicline + Bupropion (d = 0·39; p = 0·004) and Varenicline + Placebo (d = 0·31; p = 0·004) groups vs Placebo + Placebo (Fig. 2b). There was no significant difference between the Varenicline + Placebo and Varenicline + Bupropion (d = 0·02; p = 0·971) groups, precluding further comparisons.

**Table 2:** Primary outcomes, mITT.

Treatments	Coefficient estimate	CI (2·5%)	CI (97·5%)	p-value	Cohen's d for difference score
**Estimates of treatment effects on B-PEth, mITT**					
Var + Bup vs Pla + Pla	−0·176	−0·293	−0·058	0.004	0·394
Var + Pla vs Pla + Pla	−0·182	−0·304	−0·060	0.004	0·314
Var + Pla vs Var + Bup	−0·002	−0·123	0·119	0.97	0·022
Pla + Bup vs Var + Bup	0·099	−0·033	0·230	0.14^a^	0·220
Pla + Bup vs Pla + Pla	−0.072	−0·207	0·062	0.29^a^	0·138
**Estimates of treatment effects on % HDD, mITT**					
Var + Bup vs Pla + Pla	−0·106	−0·184	−0·028	0·008	0·312
Var + Pla vs Pla + Pla	−0·092	−0·171	−0·013	0·023	0·358
Var + Pla vs Var + Bup	0·013	−0·068	0·093	0·76	0·027
Pla + Bup vs Var + Bup	0·021	−0·062	0·104	0·62^a^	0·049
Pla + Bup vs Pla + Pla	−0·089	−0·171	−0·006	0·04^a^	0·252

Abbreviations: B-PEth, phosphatidylethanol in blood; Bup, bupropion; CI, confidence interval; HDD, heavy drinking days; mITT, modified intention to treat; Pla, placebo; Var, varenicline.

a Lower in the hierarchy than the first non-significant result.

The %HDD outcome was reduced in all groups from Visit 2 through Visit 9 (Fig. 2c; mITT analyses). In the mITT-analysis of the predetermined steady-state analysis period (i.e., data retrieved from Visit 4 through Visit 8) (Table 2), %HDD was significantly reduced compared to Placebo + Placebo in the Varenicline + Bupropion (d = 0·31; p = 0·008) and Varenicline + Placebo (d = 0·36; p = 0·023) groups (Fig. 2d). There was no significant difference between the Varenicline + Placebo and Varenicline + Bupropion (d = 0·03; p = 0·756) groups, precluding further comparisons.

**Fig. 3:**
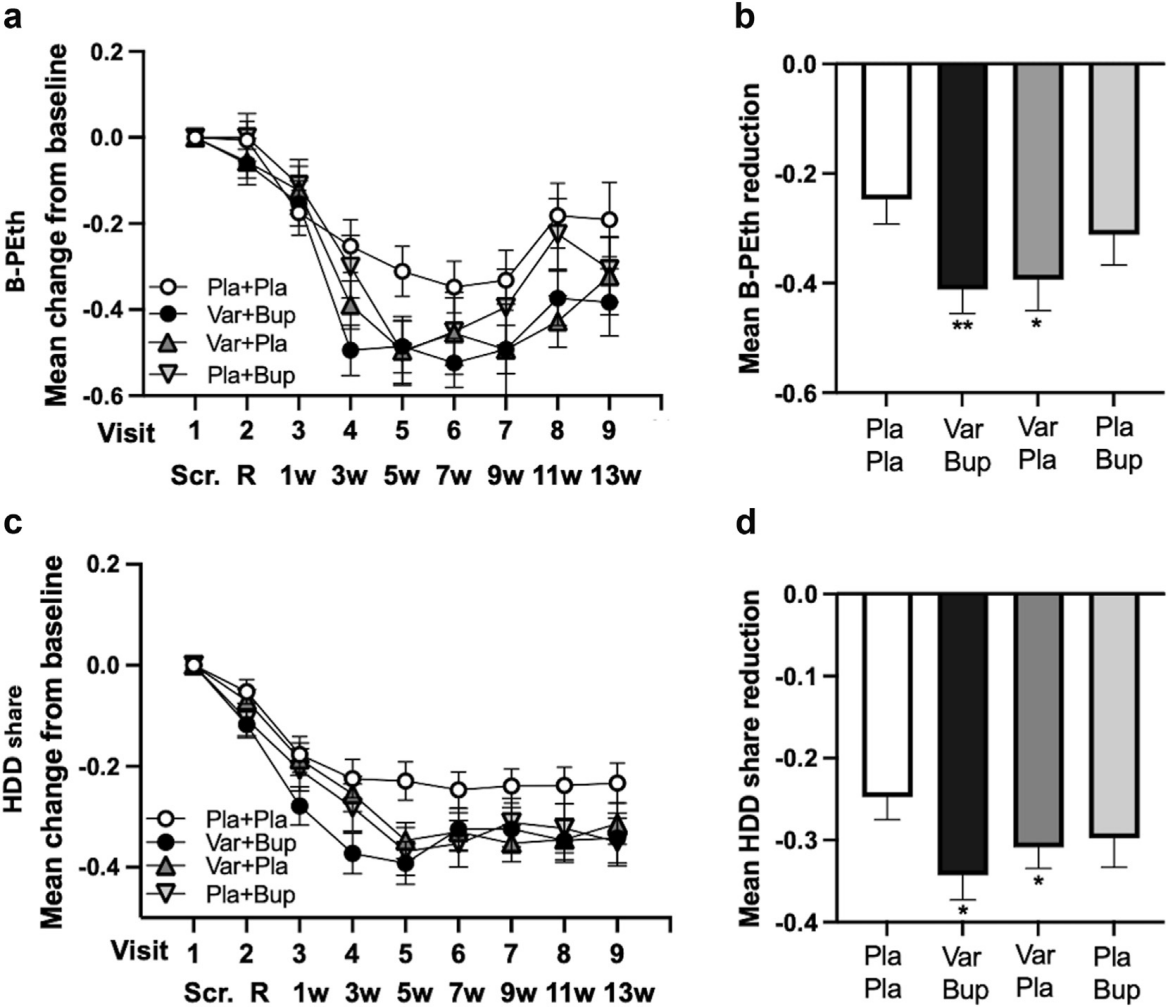
**Mean change from baseline (±SEM) of primary outcome variables B-PEth and HDD share according to the per-protocol analysis**. a (B-PEth) and c (HDD share) show the mean change at the respective visit (±SEM). b (B-PEth) and d (HDD share) show the mean reduction (±SEM) over visits 4–8, the pre-determined, steady-state period over which statistics were performed. Significance levels and effect sizes (Cohen's d) are presented in Tables 3 and in heat maps (Fig. 3, Supplement). B-PEth, phosphatidylethanol in blood; Bup, bupropion; HDD, heavy drinking days; Pla, placebo; SEM, standard error mean; Var, varenicline; Scr., screening day; R, randomisation day; w, week. ∗p = 0·029 vs Pla + Pla regarding B-PEth. ∗∗p = 0·007 vs Pla + Pla regarding B-PEth. ∗p = 0·012 vs Pla + Pla regarding HDD share for Var + Bup. ∗p = 0·044 vs Pla + Pla regarding HDD share for Var + Pla.

In the PP analysis of the predetermined analysis period (Table 3, Fig. 3), B-PEth was significantly reduced in the Varenicline + Bupropion group vs Placebo + Placebo (p = 0·007) with a larger effect size than in the mITT-analysis (d = 0·43). B-PEth was also significantly reduced by Varenicline + Placebo vs Placebo + Placebo (p = 0·029), although the effect size was smaller than in the mITT analysis (d = 0·31). There was no significant difference between the Varenicline + Placebo and Varenicline + Bupropion (d = 0·04; p = 0·778) groups, precluding further comparisons.

**Table 3:** Primary outcomes, PP.

Treatments	Coefficient estimate	CI (2·5%)	CI (97·5%)	p-value	Cohen's d for difference score
**Estimates of treatment effects on B-PEth, PP**					
Var + Bup vs Pla + Pla	−0·192	−0·332	−0·053	0·007	0·43
Var + Pla vs Pla + Pla	−0·170	−0·323	−0·018	0·029	0·307
Var + Pla vs Var + Bup	0·020	−0·123	0·163	0·78	0·043
Pla + Bup vs Var + Bup	0·135	−0·023	0·292	0·09^a^	0·21
Pla + Bup vs Pla + Pla	−0·058	−0·228	0·112	0·50^a^	0·175
**Estimates of treatment effects on % HDD, PP**					
Var + Bup vs Pla + Pla	−0·117	−0·208	−0·026	0·012	0·386
Var + Pla vs Pla + Pla	−0·089	−0·176	−0·003	0·044	0·280
Var + Pla vs Var + Bup	0·022	−0·067	0·111	0·63	0·128
Pla + Bup vs Var + Bup	0·017	−0·077	0·112	0·72^a^	0·079
Pla + Bup vs Pla + Pla	−0·101	−0·197	−0·005	0·04^a^	0·279

Abbreviations: B-PEth, phosphatidylethanol in blood; Bup, bupropion; CI, confidence interval; HDD, heavy drinking days; mITT, modified intention to treat; Pla, placebo; Var, varenicline.

a Lower in the hierarchy than the first non-significant result.

The PP analysis of the predetermined analysis period also showed that %HDD was significantly reduced by Varenicline + Bupropion vs Placebo + Placebo (p = 0·012) with a larger effect size than in the mITT-analysis (d = 0·39) (Table 3, Fig. 3). The %HDD was also significantly reduced by Varenicline + Placebo vs Placebo + Placebo (p = 0·044), although the effect size was smaller than in the mITT-analysis (d = 0·28). There was no significant difference between the Varenicline + Placebo and Varenicline + Bupropion (d = 0·13; p = 0·628) groups, precluding further comparisons.

**Fig. 4:**
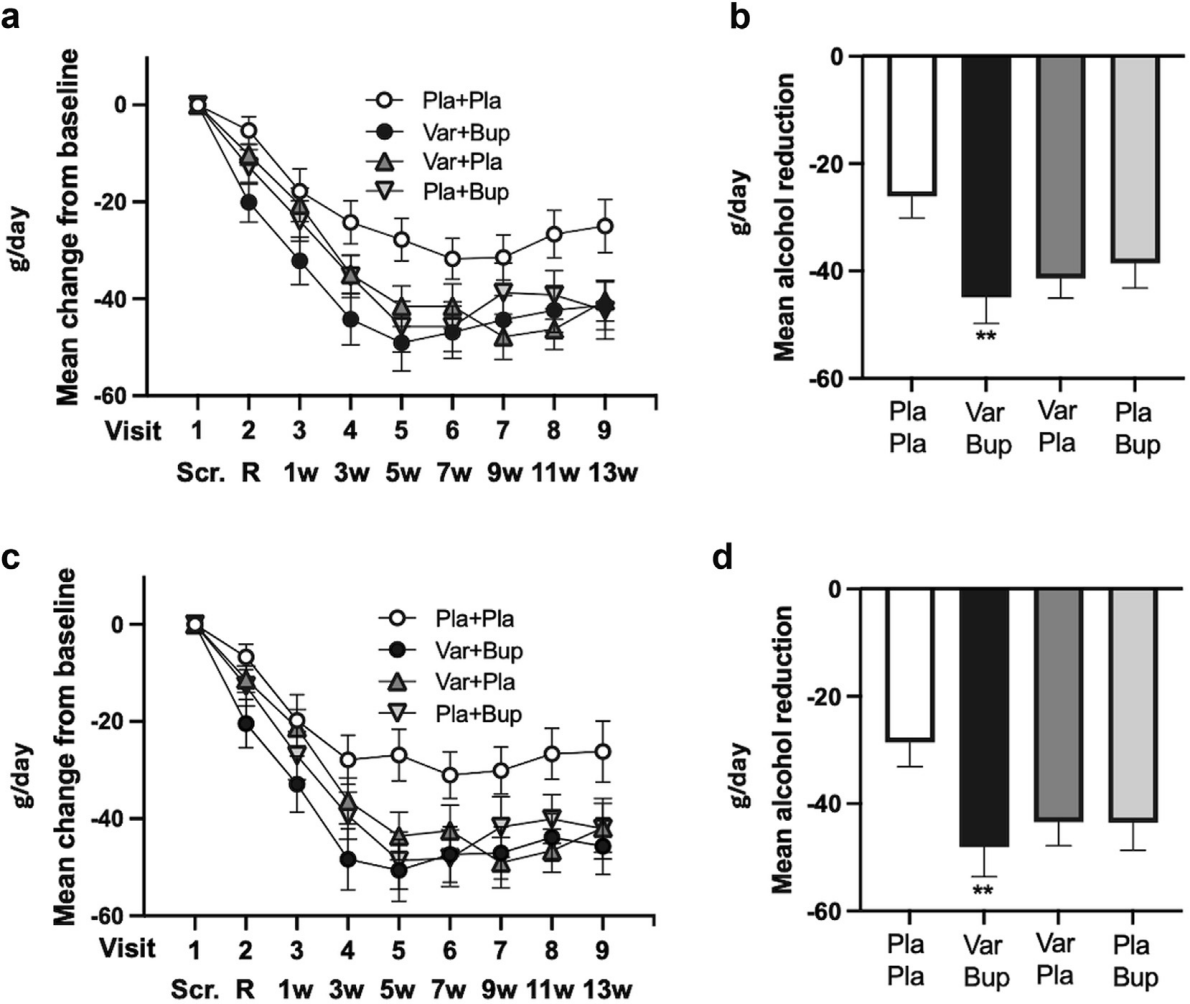
**Mean change from baseline (±SEM) of secondary outcome variable total consumption of alcohol (g/day) according to the modified intention-to-treat and per-protocol analyses**. a (mITT) and c (PP) show the mean change at the respective visit (±SEM). b (mITT) and d (PP) show the mean reduction (±SEM) over visits 4–8, the pre-determined, steady-state period over which statistics were performed. Significance levels and effect sizes (Cohen's d) are presented in Tables 3 and 4 in the Supplement and in heat maps (Fig. 3, Supplement). B-PEth, phosphatidylethanol in blood; Bup, bupropion; HDD, heavy drinking days; mITT, modified intention-to-treat; Pla, placebo; PP, per-protocol; SEM, standard error mean; Var, varenicline; Scr., screening day; R, randomisation day; w, week. ∗∗p = 0·002 vs Pla + Pla regarding mITT. ∗∗p = 0·005 vs Pla + Pla regarding PP.

Results from analyses of the secondary outcomes are given in the Appendix (Tables S3 and S4). When secondary outcomes derived from the TLFB were corrected for multiple testing, the only remaining significant finding was that for total consumption of alcohol (g/day), which was significantly reduced only in the Varenicline + Bupropion group vs Placebo + Placebo, in both the mITT (d = 0·44; p = 0·002) (Fig. 4a and b) and PP (d = 0·47; p = 0·005) analyses (Fig. 4c and d).

Alcohol craving was significantly reduced in the Varenicline + Placebo group vs Placebo + Placebo (d = 0·43; p = 0·005), following correction for multiple testing, however only in the PP-analysis.

**Figure S3:**
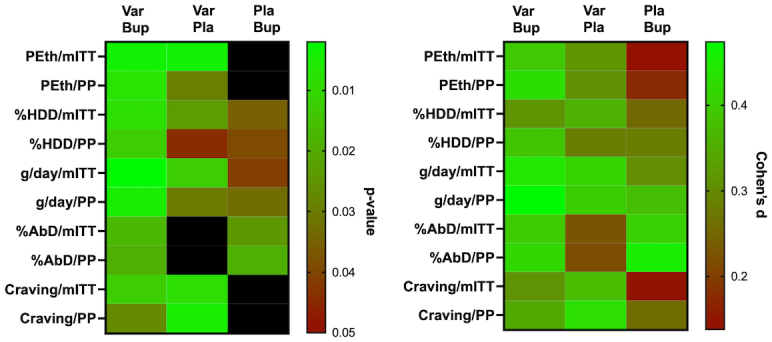


Heat maps summarizing all pair-wise statistical comparisons (significance levels) vs Placebo + Placebo and effect sizes (d) for both primary and secondary endpoints can be found in Figure S3 in the Appendix.

Serious adverse events were evenly distributed between treatment arms and none was considered to have a high probability of being related to treatment. No suspected, unexpected serious adverse reactions were recorded and both drugs were well tolerated (Table 4). The most frequent adverse event was headache followed by nausea, nasopharyngitis, insomnia, depressed mood, and fatigue that occurred in over 5% of the study population. All adverse events except nausea and fatigue were evenly distributed across treatment groups (Table 4).

**Table 4:** Adverse events.

	Var + Bup	Var + Pla	Pla + Pla	Pla + Bup	Total
Serious adverse event, per treatment group, by system organ class					
Upper abdominal pain		1			1
Acute myocardial infarction		1			1
Atrial defibrillation			1	1	2
Breast cancer			1		1
Acute cardiac failure	1				1
Acute cholecystitis				1	1
Malignant melanoma				1	1
Prostate cancer			1		1
Pulmonary embolism	1				1
Tachyarrhythmia		1			1
Total	2	3	3	3	11
Adverse event ≥5% per treatment group, by preferred term					
Headache	39	35	40	27	141
Nausea	38	61	11	12	122
Nasopharyngitis	27	26	23	18	94
Insomnia	18	16	10	15	59
Depressed mood	13	15	16	13	57
Abnormal dreams	18	23	12	9	62
Fatigue	9	23	11	12	55
Dizziness	5	14	1	15	35
Hepatic enzyme elevation	3	8	11	7	29
Anxiety	9	9	2	7	27
Dry mouth	11	5	4	6	26
Abnormal blood pressure	6	6	7	4	23
Diarrhoea	3	8	8	4	23
Cough	7	8	2	6	23
Vertigo	8	6	3	5	22
Sleep disorder	9	4	6	2	21
Arthralgia	4	3	7	7	21
Pyrexia	6	3	3	8	20
Back pain	1	6	7	5	19
Total	400	460	326	315	1501

Abbreviations: Bup, bupropion; Pla, placebo; Var, varenicline.

**Fig. 5:**
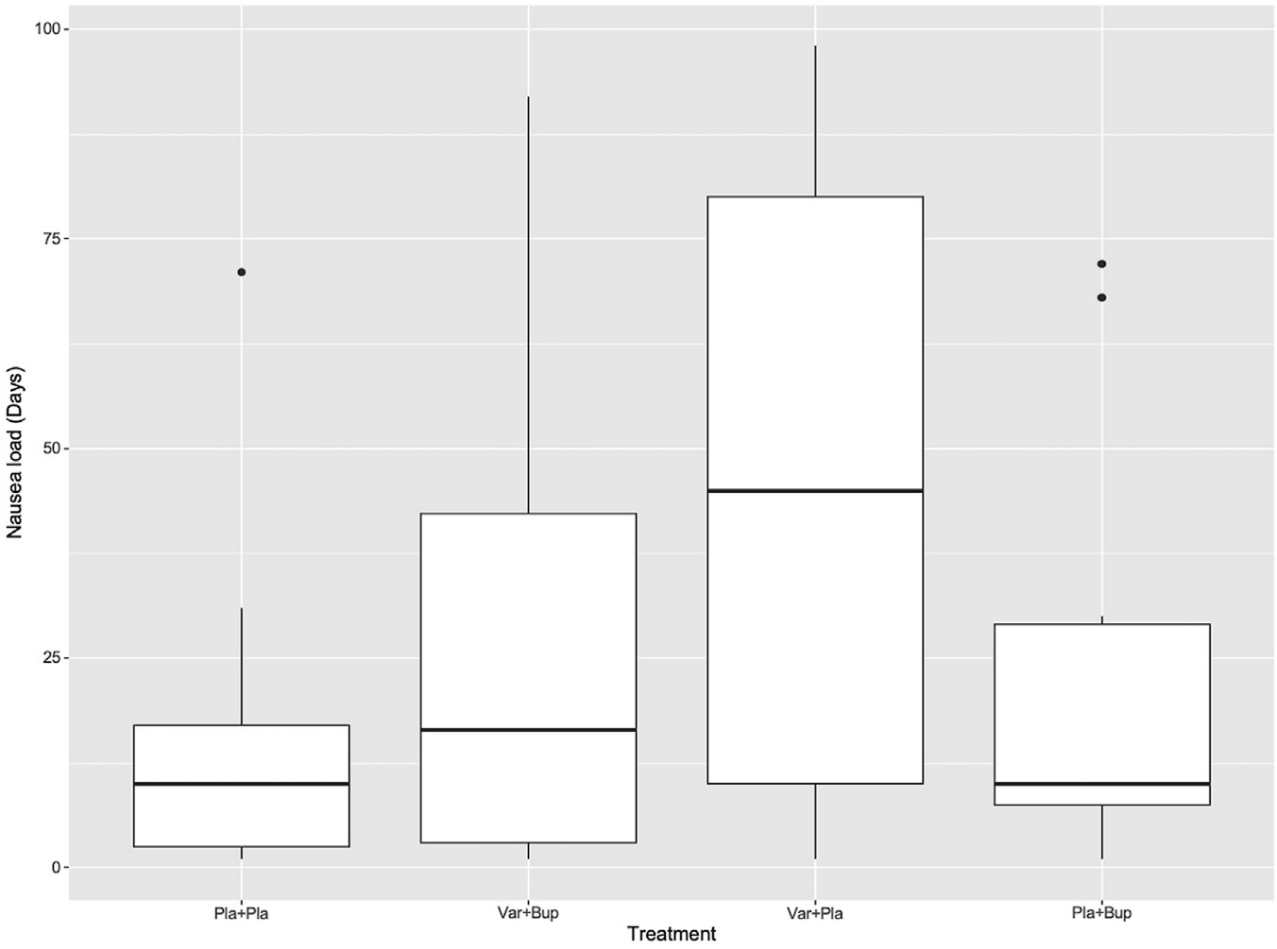
**Nausea load across the four treatment groups**. Box plot shows the nausea load (median number of days with nausea in participants experiencing nausea) across the four treatment groups. Shown are the medians (fat horizontal bars), first and third quartiles (the lower and upper borders of the boxes), minimum and maximum values (ends of vertical bars) and outliers (dots) Var + Pla vs Pla + Pla, p = 0·001; Var + Pla vs Var + Bup, p = 0·010; Var + Bup vs Pla + Pla, p = 0·186. Bup, bupropion; Pla, placebo; Var, varenicline.

Nausea occurred more commonly in the Varenicline + Placebo group than in the Placebo + Placebo group (participants with at least one day of nausea: 49/96 vs 11/97, p < 0·0001). Nausea load (median number of days with nausea in participants experiencing nausea) was also higher in the Varenicline + Placebo than in the Placebo + Placebo group (45 (70) vs 10 (14·5) days, p = 0·001). The Varenicline + Bupropion-treated participants reported statistically significant less nausea than those treated with Varenicline + Placebo (36/100 (36%) vs 49/96 (51%), p = 0·048). For the Varenicline + Bupropion group, the median duration of nausea was also significantly reduced compared to the Varenicline + Placebo group (16·5 (39·3) vs 45 (70) days, p = 0·010), and did not significantly differ from the Placebo + Placebo group (16·5 (39·3) vs 10 (14·5) days, p = 0·186) (Fig. 5) (Appendix Tables S5 and S6).

## Discussion

This randomized-controlled, double-blind, four-arm, multicentre study suggests that Varenicline + Bupropion is a potential pharmacotherapy for AUD. The mITT analysis showed that in combination, Varenicline + Bupropion reduced alcohol intake in moderate-to-severe AUD compared to Placebo + Placebo for both primary outcomes (i.e., the objective measure B-PEth and the self-reported %HDD) with effect sizes (d; 0·39 and 0·31, respectively) larger than reported in a meta-analysis of established pharmacological AUD treatments naltrexone and acamprosate (d = 0·2).^7^ The PP analyses of participants with high compliance revealed statistically significant effects with even larger effect sizes (d = 0·43 [B-PEth] and 0·39 [%HDD]). Additionally, Varenicline alone outperformed placebo on both primary endpoints with effect sizes (d = 0·29–0·36) in line with previous reports.^15^^,^^16^ In contrast, the reducing effects of Bupropion alone were smaller (d = 0·14–0·29) and did not differ significantly from placebo, according to the hierarchical statistical analysis applied.

Because of the hypothesis-driven nature of the study, secondary outcomes were analysed with and without correction for multiple testing. With correction, only the reduced total consumption of alcohol (g/day) produced by the Varenicline + Bupropion combination was significant compared to placebo, both in the mITT (d = 0·44) and PP (d = 0·47) analyses. In the non-corrected analyses, Varenicline + Bupropion also reduced alcohol craving and increased % abstinent days. Varenicline or Bupropion alone failed to show significant beneficial affects across all three, self-reported secondary outcomes.

Data were analysed with respect to mean differences between baseline values and mean values over a pre-specified time-period (10 weeks), and not by differences between baseline and study end-point. The advantage of this was that baseline values were compared to a “sum” measure over a time-period when the study medication was at steady state. Thus, this reflected the effect on the load of alcohol intake over time, which is a clinically meaningful measure in line with the harm reduction strategy. A potential disadvantage was that the effect might be temporary and not present at study end. However, the time-line figures indicated that the effects were sustained over time compared to both Placebo + Placebo and baseline values.

There were no significant differences in outcomes between Varenicline + Bupropion and Varenicline + Placebo. However, and in accordance with our hypothesis, effect sizes were generally larger for the combination, especially in the PP analyses. Taken together with the failure of Varenicline + Placebo and Placebo + Bupropion to alter the % abstinent days and craving for alcohol, respectively, in non-corrected analyses, combined treatment appears to add treatment value. This was illustrated in heat maps (Appendix) that also suggest that the effects are more consistent across variables with combination treatment.

Both drugs were well tolerated, and no serious adverse events were considered to have a high probability of being related to treatment. Adverse events were evenly distributed across treatment groups, except nausea and fatigue, which occurred most frequently in the Varenicline + Placebo group. There was no increased risk for adverse events when combining the drugs, although a hypothetical, increased risk when used over longer periods of time (>12 weeks) cannot be excluded. However, the present study showed evidence to the contrary since both the incidence of nausea and the median number of days with nausea were significantly reduced in participants receiving Varenicline + Bupropion compared to Varenicline + Placebo, and the duration of nausea in the combination group did not differ significantly from in the Placebo + Placebo group. This nausea-reducing effect of Bupropion when added to Varenicline may be advantageous not only when treating AUD, but also when using Varenicline and Bupropion for smoking cessation, a currently approved indication. Varenicline-induced nausea is a frequent and troublesome adverse event in smoking cessation studies.^22^ The mechanism underlying this beneficial effect of adding Bupropion is unknown, but may involve antagonistic effects at nicotinic (e.g., alpha-3 beta-4 [α3β4] subtypes) or 5-HT3A receptors exerted by Bupropion and its metabolite OH-bupropion^23^ that counteract nicotine- and/or 5-HT3A-receptor-induced nausea by Varenicline.^24^

The findings support our hypothesis that drugs that elevate brain dopamine levels reduce alcohol intake, and thereby indirectly also the dopamine deficiency hypothesis of AUD. In the three active arms that used drugs known to increase brain dopamine levels, beneficial effects on one or more of the primary and/or secondary alcohol-related outcomes were found. Furthermore, theoretically—and according to our recent rat study^18^—the Varenicline + Bupropion combination should raise dopamine levels more than the respective drugs alone. Accordingly, this combination displayed more consistent effects across outcome variables and larger effect sizes, especially in the PP analyses. The PP analysis for B-PEth arguably is the most relevant when comparing to the rat study.^18^ Firstly, the PP population was exposed to the drugs to a higher degree than the mITT population, as were the rats. Secondly, B-PEth is an objective measure of ethanol intake and ethanol intake was measured objectively in the rats. Indeed, the PP results for B-PEth largely mirror the outcome of our rat study.^18^ On the other hand, no significant differences were found between the combination and Varenicline or Bupropion alone, which would argue against the dopamine deficiency hypothesis. However, this result could also be explained by a lack of power to detect such a difference and/or by the doses being near the peak of the respective dose–response curves, where the additive dopamine effect may be smaller, as indicated in the aforementioned rat study.^18^

There could also be another dopamine-related explanation for the present results. Since nAChRs are involved in the dopamine-releasing and positive reinforcing effects of ethanol,^22^ the partial antagonistic effects of Varenicline on nAChRs may have contributed to the alcohol-intake reducing effects we observed. Experimental studies in humans support this theory for Varenicline,^25^ while animal studies do not,^26^ most likely because the nAChR subtypes involved in these actions of ethanol in rats differ from those affected by Varenicline.^22^ Finally, it should be noted that both drugs interfere also with other than dopaminergic mechanisms, e.g., noradrenergic, and thus the above explanations to the observed effects at this stage remain speculative.

Based on epidemiological estimates on the relationship between daily alcohol intake in grams and the risk of dying from an alcohol-related cause,^27^ and if maintained over time, the mean reduction of daily alcohol intake subjectively reported in the Varenicline + Bupropion PP group would represent a reduction of risk of death from alcohol-related causes from approximately 25%–6%, i.e., a risk reduction of 76%. Similarly, according to the objective measure B-PEth, the risk would be reduced from approximately 25%–7·5%, a 70% risk reduction. Based on our previous study,^17^ the baseline B-PEth levels observed here would correspond to a total consumption of alcohol of approximately 105 g/day that corresponds well with baseline values in the present study.

In our previous study, Varenicline reduced B-PEth, but did not significantly alter %HDD vs placebo, presumably due to a strong placebo effect on the latter, but not the former measure.^16^ Here, we observed significant effects of Varenicline both on B-PEth and self-reported %HDD. Methodological differences between the two studies likely contribute to the greater concordance of objective and self-reported drinking reductions in the current study. Here, we used a validated TLFB interview^20^ to retrieve alcohol consumption data, while the previous study used a simpler diary methodology.^16^ Furthermore, the current study employed inclusion criteria that resulted in a study population with a higher severity of AUD, and a higher alcohol intake. Accordingly, the B-PEth values at baseline were higher in the current study than in the previous study (1·2 vs 0·8 μmol/L).^16^

The dopamine-elevating properties of Varenicline and Bupropion have important clinical advantages compared to those of, for example, central stimulants. Both drugs have ceiling effects since Varenicline is a partial agonist at nAChRs,^14^ while Bupropion occupies only a minority of available dopamine reuptake proteins.^28^ This limits dose-dependent increases in dopamine elevations, thereby minimizing abuse liability. Indeed, neither Varenicline nor Bupropion has been associated with significant misuse. Both are effective and safe for smoking cessation across various populations and have been recommended as treatments for psychiatric or addictive comorbidity.^29^ A further advantage of the Varenicline + Bupropion combination is its effectiveness for smoking cessation since individuals with AUD are known to have a higher risk of smoking than the general population. Here, we found that 49% of participants were daily nicotine users compared to 29% of the Swedish population having used any nicotine product over the last 30 days.^30^ Unfortunately, the objective test for nicotine use (cotinine test) was withdrawn from the market during this study, precluding analysis of treatment effects on nicotine use and on a tentative relationship between this effect and that on alcohol intake. Previous studies of Varenicline on AUD, however, have indicated that the alcohol intake-reducing effect is independent of smoking status.^15^^,^^16^

Amongst the strengths of this proof-of-concept, hypothesis-testing study is the population homogeneity across sites based on time to inclusion criteria of the recruitment protocol. Because we analysed medication effects on alcohol use without preceding medically-assisted withdrawal treatment (“detoxification”), our findings are likely generalizable to the majority of individuals with AUD, most of whom do not require this. Compliance to the study protocol and treatment compliance was high, despite high drop-out rates typically being seen in AUD treatment. The study included B-PEth as one of two primary outcomes, thus minimising recall bias and other reasons for misreporting alcohol consumption. As part of the inclusion criteria, B-PEth levels also enabled objective assessment of baseline alcohol levels and inclusion of participants with assured high alcohol consumption.

Our study also has limitations. The lack of initial detoxification, while improving generalizability, also precludes an analysis of the extent to which the study medications maintain abstinence. Study population distribution between sites was uneven, although this was addressed in the statistical analysis. Due to unforeseen events, no analyses on secondary outcomes nicotine use and Continuous Performance Test + Activity test were possible. Another limitation may be the sequential hierarchical statistical method used as this requires a predetermined order of tests that reduces the likelihood of a significant outcome of the last tests compared to the first ones. Thus, lack of significance for Placebo + Bupropion, for example, may be false due to the hierarchical analysis position. However, effect sizes vs Placebo + Placebo were also generally smaller for Placebo + Bupropion compared to the other groups. The decision not to conduct imputations could also represent a limitation, although this was a response to the small proportion of missing data, whereas the groups with missing values were sufficiently large. With large sample sizes, a small amount of missing data is unlikely to significantly affect the overall statistical properties, ensuring that the estimates remain unbiased and robust. Furthermore, although participants with moderate to severe AUD were included, recruitment by advertising in combination with the exclusion criteria used (e.g., excluding patients that had received alcohol withdrawal treatment within 30 days of study initiation, or any pharmacological/non-pharmacological treatment affecting alcohol consumption 3 months pre-screening) may have biased the population towards socially stable and medically healthy participants. A study strength, however, was that this population selection facilitated adherence. Finally, this study was performed in Sweden, and healthcare systems and other relevant conditions, e.g., ethnic diversity, may be different in other countries. However, both the study setting and the characteristics of the study population at a minimum are likely to make the results generalizable to other industrialized countries.

In conclusion, our results suggest that treatments that enhance central dopamine neurotransmission reduce alcohol consumption measured by B-PEth or %HDD in moderate-to-severe AUD, a finding lending indirect pharmacological support to the dopamine deficiency hypothesis of AUD. The results of this hypothesis-driven study also indicate that the combination of Varenicline + Bupropion is a promising, well-tolerated treatment for AUD, with an effect size approximately double that of available pharmacological treatments for AUD. Our findings also suggest that Bupropion significantly counteracts Varenicline-induced nausea, a common side effect. Finally, our findings support the utility of the biomarker B-PEth as a primary outcome measure in RCTs on AUD.

## Contributors

All authors had full access to all study data, contributed to the reported study, and are accountable for all aspects of the work and its publication. AdB, BS were involved in the visualisation, conceptualization and design of the study. BA, AdB, BS, CW-N were involved in study data curation, including access and verification. AdB, HL, BS were involved in the investigation of the study. BA, AdB, JF, JG, AH, MH, HL, DL, MS, BS, CW-N were involved in the study methodology. BA, AdB, HL, BS, CW-N were involved in the supervision of the study and project administration. AdB, JF, AH, MH, HL, BS were involved in the funding acquisition. AdB, JF, AH, MH, HL, DL, BS were involved in acquiring resources for the study. AdB, BS were involved in writing the original draft of the manuscript, and all authors (BA, AdB, JF, JG, AH, MH, HL, DL, MS, BS, CW-N) were involved in writing, critically reviewing, and revising all subsequent drafts of the manuscript and provided approval for the submission/publication of the final manuscript. Non-author contributors include Dr Grażyna Söderbom (writing and editorial support for manuscript development) and Prof. Mia Ericson (preparation of the figures for the manuscript), as well as additional contributors named in the acknowledgements.

## Data sharing statement

De-identified data on a group level and underlying analyses in the result section will be made available by the PI (Bo Söderpalm) after approval of the proposal and data management agreement.

## Declaration of interests

Andrea de Bejczy and Bo Söderpalm are the founders and co-owners of Sobrera Pharma AB. Helga Lidö, Barbro Askerup, and Cecilia Nilsson-Wallmark are co-owners of Sobrera Pharma AB. Bo Söderpalm has received honoraria for lectures from Lundbeck, Takeda, and Evolan. Markus Heilig has received research funding or consulting fees in the past 5 years from Aelis Farma, Brainsway Technologies, Camurus, Indivior, Janssen, Molteni, Nordic Drugs, and Pfizer. Daniel Lindqvist has received research grants from Biogaia AB and honoraria for lectures from Janssen-Cilag AB and H. Lundbeck AB. Johan Franck, Anders Håkansson, Joar Guterstam and Markus Samuelson report no conflicts of interest. This study is an academic study and all decisions concerning the design, execution, analysis, and publications have been and are made by the academic research group/sponsor team with PI/sponsor Bo Söderpalm. Sobrera Pharma has not produced or supplied any of the study drugs or had any involvement in the statistical analysis or data interpretation.

## Figures and Tables

**Figure S1:**
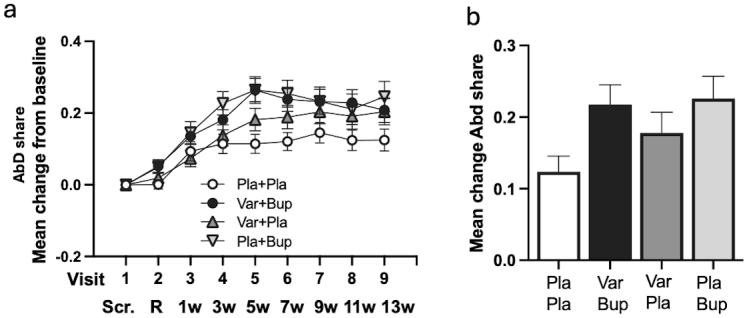


**Figure S2:**
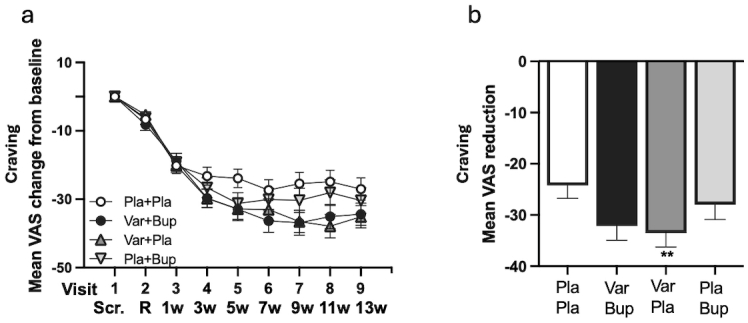


## Appendix A Supplementary data

Renamed 54850

Renamed af0a8
