# Supplementary material for: Efficacy and safety of varenicline and bupropion, in combination and alone, for alcohol use disorder: a randomized, double-blind, placebo-controlled multicentre trial
Source: Lancet Reg Health Eur. 2025 May 13;54:101310. doi: 10.1016/j.lanepe.2025.101310 (PMC12142338; doi:10.1016/j.lanepe.2025.101310)
Supplement: Renamed 54850 [file mmc1.docx]

**Table of Content**

METHODS

Study setting and design …….2

Data management plan and statistical analysis plan, and study protocol …….2

Statistical analysis …….2

RESULTS

Table S1: Demographic characteristics of study participants in the PP B-PEth population …… 3

Table S2: Demographic characteristics of study participants in the PP HDD population …….5

Secondary outcomes, uncorrected results …….6

Table S3. Secondary outcomes, mITT. …….7

Table S4. Secondary outcomes, PP (HDD population). …….8

Figure S1: Mean change from baseline (± SEM) of secondary outcome variable

abstaining days share according to the modified intention-to-treat analysis …….9

Figure S2: Mean change from baseline (± SEM) of secondary outcome variable

alcohol craving according to the modified intention-to-treat analysis …….9

Figure S3: Heat maps summarizing the uncorrected significance levels (p-values)

and effect sizes (Cohen’s d) for primary and secondary outcome variables

according to the modified intention-to-treat and per-protocol analyses ……10

Table S5. Nausea, defined as at least one report of nausea, n (%), mITT ……11

Table S6. Nausea, duration of nausea in days, in participants with at least one report of nausea,

n (%), mITT ……11

References ……11

**METHODS**

## **Study setting and design**

The study was conducted at four alcohol clinic research sites across Sweden:

- Gothenburg (Sponsor and Coordinating Centre): Addiction Biology Unit, Psychiatry and Neurochemistry Section, Institute of Neuroscience and Physiology, Sahlgrenska Academy, University of Gothenburg, Sweden & Department of Addiction and Dependency, Sahlgrenska University Hospital; PI Bo Söderpalm.
- Stockholm: Department of Clinical Neuroscience, Centre for Psychiatry Research, Karolinska Institute & Stockholm Health Care Services, Stockholm County Council; PI Johan Franck.
- Linköping: Centre for Social and Affective Neuroscience, BKV, Linköping University; PI Markus Heilig.
- Region Skåne: Lund University, Faculty of Medicine, Department of Clinical Sciences Lund, Psychiatry, Lund, Sweden & Office for Psychiatry and Habilitation, Malmö Addiction Centre, Malmö; PI Anders Håkansson.

The primary outcome and eligibility criteria B-PEth samples were analysed by a central laboratory in Sweden: Clinical Chemistry (Klinisk Kemi), Region Skåne.

The study was performed in accordance with the study protocol, the WMA Declaration of Helsinki (October, 2013), GCP principles [i.e., ICH-GCP E6(R2)], all applicable regulatory requirements (“Läkemedelsverkets föreskrifter”, LVFS 2011:9), and the EU General Data Protection Regulation (GDPR) 2016/679 privacy legislation. Ethical approval was granted by the Swedish Ethical Review Authority (EPM; D.nr. 431-18, 2018-06-18, amendment 2019-07-05/2019-11-26 D.nr. 2019-03559, amendment 2020-10-21 D.nr. 202004924, amendment 2022-01-14 D.nr 2021-06785-02). Approval has been obtained from the Swedish Medical Product Agency (MPA; EudraCT 2018–000048-24, amendment 2019-07-31 D.nr.5.1-2019-50751, amendment 2022-01-31 D.nr. 5.1-2021-101134). All necessary informed consent was provided in accordance with legal requirements.

## **Data management plan (DMP) statistical analysis plan (SAP), and study protocol**

Both the DMP and SAP are available upon request. The study protocol^1^ for the COMB Study (COMB-BO8, EudraCT 2018–000048–24; Version 3.2, Lidö & deBejczy, 2020-06-16; https://clinicaltrials.gov identifier NCT04167306) has been published and can be accessed via this link: doi: 10.1371/journal.pone.0296118. eCollection 2024.

## **Statistical analyses**

The sequential hierarchical method is used to optimize significance in hypothesis testing, where multiple tests are performed. The method evaluates the different hypotheses in a predefined sequence order, starting with the first hypothesis. If a significant difference is identified, the analysis will proceed to testing the next hypothesis in the sequence. The testing sequence is stopped at the first, non-significant test.

The sequential hierarchical method ensures that the total probability of a type 1 error is distributed over the different hypotheses tested so as to ensure that the more important hypotheses are tested first. The alternative, testing all factors and their interactions simultaneously as opposed to testing the specific contrasts of interest, would result in a lower power.

Model diagnostics were performed, and the results indicated that the statistical models met the normality assumptions.

The Cohen’s d (d) effect size was estimated for each test as per Cohen, 1988^2^ using the following equation:

 
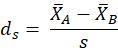
 where 
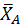
 and 
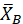
=the two-sample means, and s=the pooled within sample estimate of the population standard deviation


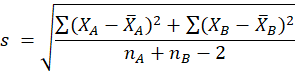


# **RESULTS**

## **Table S1: Demographic characteristics of study participants in the PP B-PEth population**

|  | **Pla+Pla (n=67)** | **Var+Bup (n=64)** | **Var+Pla (n=65)** | **Pla+Bup (n=61)** | **Total (n=257)** |
| --- | --- | --- | --- | --- | --- |
| **Baseline B-PEth (μmol/L)** |  |  |  |  |  |
| Mean (SD) | 1·2 (0·53) | 1·2 (0.71) | 1.2 (0·78) | 1·4 (0·54) | 1·2 (0·65) |
| Median [Min, Max] | 1·1 [0·50, 2·6] | 1.1 [0·52, 4·1] | 0·96 [0·52, 5·0] | 1·3 [0·50, 3·0] | 1·1 [0·50, 5·0] |
| **Baseline proportion of HDD** |  |  |  |  |  |
| Mean (SD) | 0·76 (0·25) | 0·77 (0·29) | 0·75 (0·25) | 0·77 (0·25) | 0·76 (0·26) |
| Median [Min, Max] | 0·86 [0, 1·0] | 0·93 [0, 1·0] | 0·79 [0·14, 1·0] | 0·86 [0, 1·0] | 0·86 [0, 1·0] |
| **Sex, n (%)** |  |  |  |  |  |
| Male | 44 (65·7%) | 48 (75·0%) | 49 (75·4%) | 49 (80·3%) | 190 (73·9%) |
| Female | 23 (34·3%) | 16 (25·0%) | 16 (24·6%) | 12 (19·7%) | 67 (26·1%) |
| **Age (years)** |  |  |  |  |  |
| Mean (SD) | 57 (10) | 56 (8·7) | 55 (9·6) | 58 (8·2) | 56 (9·3) |
| Median [Min, Max] | 59 [31, 70] | 58 [33, 68] | 55 [32, 70] | 58 [35, 70] | 57 [31, 70] |
| **Marital status, n (%)** |  |  |  |  |  |
| Single | 12 (18.2%) | 13 (20.3%) | 7 (10.8%) | 6 (9.8%) | 38 (14.8%) |
| Married | 33 (50.0%) | 35 (54.7%) | 37 (56.9%) | 29 (47.5%) | 134 (52.3%) |
| Partner, cohabiting | 9 (13·6%) | 8 (12·5%) | 11 (16·9%) | 12 (19·7%) | 40 (15·6%) |
| Partner, non-cohabiting | 4 (6·1%) | 2 (3·1%) | 4 (6·2%) | 6 (9·8%) | 16 (6·3%) |
| Divorced | 6 (9·1%) | 4 (6·3%) | 6 (9·2%) | 5 (8·2%) | 21 (8·2%) |
| Separated | 0 (0%) | 1 (1·6%) | 0 (0%) | 2 (3·3%) | 3 (1·2%) |
| Widowed | 2 (3·0%) | 1 (1·6%) | 0 (0%) | 1 (1·6%) | 4 (1·6%) |
| **Education, n (%)** |  |  |  |  |  |
| Preparatory high school | 28 (42·4%) | 27 (42·2%) | 30 (46·2%) | 26 (42·6%) | 111 (43·4%) |
| High school | 6 (9·1%) | 5 (7·8%) | 4 (6·2%) | 7 (11·5%) | 22 (8·6%) |
| Higher education ≤ 3 years | 15 (22·7%) | 16 (25·0%) | 19 (29·2%) | 16 (26·2%) | 66 (25·8%) |
| Higher education > 3 years | 17 (25·8%) | 16 (25·0%) | 12 (18·5%) | 12 (19·7%) | 57 (22·3%) |
| **Weight (kg)** |  |  |  |  |  |
| Mean (SD) | 86 (16) | 91 (17) | 87 (16) | 86 (18) | 87 (17) |
| Median [Min, Max] | 86 [52, 120] | 90 [51, 130] | 88 [50, 120] | 85 [53, 130] | 87 [50, 130] |
| **Age at alcohol debut (years)** |  |  |  |  |  |
| Mean (SD) | 16 (6.2) | 15 (3·4) | 15 (2·2) | 15 (3·0) | 15 (4·0) |
| Median [Min, Max] | 15 [7·0, 48] | 15 [12, 35] | 15 [7·0, 20] | 15 [11, 30] | 15 [7·0, 48] |
| **Heredity for alcohol problems, n (%)** |  |  |  |  |  |
| Yes | 10 (15·2%) | 21 (32·8%) | 7 (10·8%) | 10 (16·4%) | 48 (18·8%) |
| No | 48 (72·7%) | 43 (67·2%) | 50 (76·9%) | 47 (77·0%) | 188 (73·4%) |
| Don’t know | 8 (12·1%) | 0 (0%) | 8 (12·3%) | 4 (6·6%) | 20 (7·8%) |
| **Nicotine daily, n (%)** |  |  |  |  |  |
| Yes | 31 (46·3%) | 29 (45·3%) | 25 (38·5%) | 31 (50·8%) | 116 (45·1%) |
| No | 36 (53·7%) | 35 (54·7%) | 40 (61·5%) | 30 (49·2%) | 141 (54·9%) |

Abbreviations; B-PEth, phosphatidylethanol in blood; Bup, bupropion; HDD, heavy drinking days; Pla, placebo; PP, per protocol; SD, standard deviation; Var, varenicline.

## **Table S2: Demographic characteristics of study participants in the PP HDD population**

|  | **Pla+Pla (n=69)** | **Var+Bup (n=64)** | **Var+Pla (n=68)** | **Pla+Bup (n=63)** | **Total (n=264)** |  |
| --- | --- | --- | --- | --- | --- | --- |
| **Baseline B-PEth (μmol/L)** |  |  |  |  |  |  |
| Mean (SD) | 1·2 (0·54) | 1·2 (0·71) | 1·2 (0·77) | 1·3 (0·54) | 1·2 (0·65) |  |
| Median [Min, Max] | 1·1 [0·50, 2·6] | 1·1 [0·52, 4·1] | 0·96 [0·52, 5·0] | 1·3 [0·50, 3·0] | 1·1 [0·50, 5·0] |  |
| **Baseline proportion of HDD** |  |  |  |  |  |  |
| Mean (SD) | 0·76 (0·25) | 0·77 (0·29) | 0·76 (0·25) | 0·77 (0·26) | 0·76 (0·26) |  |
| Median [Min, Max] | 0·86 [0, 1·0] | 0·93 [0, 1·0] | 0·79 [0·14, 1·0] | 0·86 [0, 1·0] | 0·86 [0, 1·0] |  |
| **Baseline grams alcohol/day** |  |  |  |  |  |  |
| Mean (SD) | 99·1 (37·6) | 107 (55·2) | 99·3 (41·1) | 106 (40·3) | 103 (43·9) |  |
| Median [Min, Max] | [35·2, 205] | [34·1, 362] | [21·0, 210] | [363·, 243] | [21·0, 362] |  |
| **Sex, n (%)** |  |  |  |  |  |  |
| Male | 45 (65·2%) | 48 (75·0%) | 50 (73·5%) | 51 (81·0%) | 194 (73·5%) |  |
| Female | 24 (34·8%) | 16 (25·0%) | 18 (26·5%) | 12 (19·0%) | 70 (26·5%) |  |
| **Age (years)** |  |  |  |  |  |  |
| Mean (SD) | 57 (10) | 56 (8·7) | 55 (9·5) | 58 (8·2) | 56 (9·2) |  |
| Median [Min, Max] | 58 [31, 70] | 58 [33, 68] | 55 [32, 70] | 57 [35, 70] | 57 [31, 70] |  |
| **Marital status, n (%)** |  |  |  |  |  |  |
| Single | 13 (19·1%) | 13 (20·3%) | 7 (10·3%) | 7 (11·1%) | 40 (15·2%) |  |
| Married | | 33 (48·5%) | 35 (54·7%) | 39 (57·4%) | 30 (47·6%) | 137 (52·1%) |
| Partner, cohabiting | 10 (14·7%) | 8 (12·5%) | 11 (16·2%) | 12 (19·0%) | 41 (15·6%) |  |
| Partner, non-cohabiting | 4 (5·9%) | 2 (3·1%) | 4 (5·9%) | 6 (9·5%) | 16 (6·1%) |  |
| Divorced | 6 (8·8%) | 4 (6·3%) | 7 (10·3%) | 5 (7·9%) | 22 (8·4%) |  |
| Separated | 0 (0%) | 1 (1·6%) | 0 (0%) | 2 (3·2%) | 3 (1·1%) |  |
| Widowed | 2 (2·9%) | 1 (1·6%) | 0 (0%) | 1 (1·6%) | 4 (1·5%) |  |
| **Education, n (%)** |  |  |  |  |  |  |
| Preparatory high school | 6 (8·8%) | 5 (7·8%) | 4 (5·9%) | 8 (12·7%) | 23 (8·7%) |  |
| High school | 29 (42·6%) | 27 (42·2%) | 31 (45·6%) | 27 (42·9%) | 114 (43·3%) |  |
| Higher education ≤ 3 years | 15 (22·1%) | 16 (25·0%) | 21 (30·9%) | 16 (25·4%) | 68 (25·9%) |  |
| Higher education >3 years | 18 (26·5%) | 16 (25·0%) | 12 (17·6%) | 12 (19·0%) | 58 (22·1%) |  |
| **Weight (kg)** |  |  |  |  |  |  |
| Mean (SD) | 86 (17) | 91 (17) | 87 (17) | 85 (18) | 87 (17) |  |
| Median [Min, Max] | 86 [52, 120] | 90 [51, 130] | 88 [50, 120] | 85 [53, 130] | 87 [50, 130] |  |
| **Age at alcohol debut (years)** |  |  |  |  |  |  |
| Mean (SD) | 16 (6·1) | 15 (3·4) | 15 (3·3) | 15 (2·9) | 16 (4·2) |  |
| Median [Min, Max] | 15 [7·0, 48] | 15 [12, 35] | 15 [7·0, 35] | 15 [11, 30] | 15 [7·0, 48] |  |
| **Heredity for alcohol problems, n (%)** |  |  |  |  |  |  |
| Yes | 10 (14·7%) | 21 (32·8%) | 7 (10·3%) | 11 (17·5%) | 49 (18·6%) |  |
| No | 50 (73·5%) | 43 (67·2%) | 53 (77·9%) | 48 (76·2%) | 194 (73·8%) |  |
| Don’t know | 8 (11·8%) | 0 (0%) | 8 (11·8%) | 4 (6·3%) | 20 (7·6%) |  |
| **Nicotine, daily, n (%)** |  |  |  |  |  |  |
| Yes | 32 (46·4%) | 29 (45·3%) | 27 (39·7%) | 33 (52·4%) | 121 (45·8%) |  |
| No | 37 (53·6%) | 35 (54·7%) | 41 (60·3%) | 30 (47·6%) | 143 (54·2%) |  |

Abbreviations; B-PEth, phosphatidylethanol in blood; Bup, bupropion; HDD, heavy drinking days; Pla, placebo; PP, per protocol; SD, standard deviation; Var, varenicline.

**Secondary outcomes, uncorrected results**

With no correction made for multiple testing, total consumption of alcohol (g/day) was significantly reduced in the Varenicline+Placebo group vs. the Placebo+Placebo group, both in the mITT (d=0·41, p=0·012) and PP (d=0·40, p=0·030) analyses. It was also reduced by Placebo+Bupropion vs. Placebo+Placebo in the mITT (d=0·30, p=0·041) and PP (d=0·38, p=0·033) analyses (tables S3 and S4).

With no correction for multiple testing, the share of abstinent days was significantly increased in the Varenicline+Bupropion group vs. Placebo+Placebo in both the mITT (d=0·39, p=0·017) (figure 1a,b) and the PP (d=0·42, p=0·019) analyses. This was the case also for the Placebo+Bupropion group vs. Placebo+Placebo in both analyses (mITT d=0·41, p=0·024; PP d=0·46, p=0·019) (figure 1a,b), whereas no significant difference was shown between Varenicline+Placebo and Placebo+Placebo in either analysis (mITT d=0·23, p=0·317; PP d=0·22, p=0·277) (figure 1a,b) (tables S3 and S4).

Alcohol craving was significantly reduced in the Varenicline+Bupropion group vs. Placebo+Placebo in both analyses (mITT d=0·31, p=0·020 (figure 2a,b); PP d=0·34, p=0·027) when no correction for multiple testing was applied. This was also the case for the Varenicline+Placebo group vs. Placebo+Placebo (mITT d=0·37, p=0·008 (figure 2a,b); PP d=0·43, p=0·005), whereas this measure did not differ significantly between Placebo+Bupropion and Placebo+Placebo (mITT d=0·15, p=0·340; PP d=0·26, p=0·102) (figure 2a,b) (tables S3 and S4).

**Table S3. Secondary outcomes, mITT.**

| **Treatments** |  | **CI (2.5%)** | **CI (97.5%)** | **No adjusted method p-value** | **Holm-Bonferroni p-value** | **Cohen’s d for difference score** |
| --- | --- | --- | --- | --- | --- | --- |
| **Mean grams of alcohol per day, mITT** | | | | | | |
|  | **T-value** |  |  |  |  |  |
| Var+Bup vs Pla+Pla | -2·981 | -31·348 | -6·373 | 0·002 | Sig | 0·443 |
| Var+Pla vs Pla+Pla | -2·779 | -26·195 | -4·442 | 0·01 | NS | 0·412 |
| Var+Pla vs Var+Bup |  |  |  | 0·57 | NS | 0·087 |
| Pla+Bup vs Var+Bup |  |  |  | 0·30 | NS | 0·144 |
| Pla+Bup vs Pla+Pla | -2·032 | -24·655 | -0·361 | 0·04 | NS | 0·304 |
| **% Abstaining days, mITT** | | | | | | |
|  | **Estimate** |  |  |  |  |  |
| Var+Bup vs Pla+Pla | 0·070 | 0·00001 | 0·147 | 0·02 | NS | 0·395 |
| Var+Pla vs Pla+Pla | - | - | - | 0·32 | NS | 0·225 |
| Var+Pla vs Var+Bup | - | - | - | 0·26 | NS | 0·15 |
| Pla+Bup vs Var+Bup | - | - | - | 0·91 | NS | 0·031 |
| Pla+Bup vs Pla+Pla | 0·06 | 0·00005 | 0·129 | 0·02 | NS | 0·406 |
| **Alcohol craving, mITT** | | | | | | |
|  | **Estimate** |  |  |  |  |  |
| Var+Bup vs Pla+Pla | -8·000 | -15·400 | -1·400 | 0·01 | NS | 0·311 |
| Var+Pla vs Pla+Pla | -9·000 | -16·2 | -2·2 | 0·01 | NS | 0·374 |
| Var+Pla vs Var+Bup | - | - | - | 0·79 | NS | 0·054 |
| Pla+Bup vs Var+Bup | - | - | - | 0·22 | NS | 0·158 |
| Pla+Bup vs Pla+Pla | - | - | - | 0·34 | NS | 0·148 |

Abbreviations; CI, confidence interval; Bup, bupropion; mITT, modified Intention to Treat; NS, not significant; Pla, placebo; Sig, significant; Var, varenicline.

**Table S4. Secondary outcomes, PP (HDD population).**

| **Treatments** |  | **CI (2.5%)** | **CI (97.5%)** | **No adjusted method p-value** | **Holm-Bonferroni p-value** | **Cohen’s d for difference score** |
| --- | --- | --- | --- | --- | --- | --- |
| **Mean grams of alcohol per day, PP** | | | | | | |
|  | **T-value** |  |  |  |  |  |
| Var+Bup vs Pla+Pla | -2·718 | -33·700 | -5·301 | 0·005 | Sig. | 0·474 |
| Var+Pla vs Pla+Pla | -2·345 | -27·415 | -2·328 | 0·03 | NS | 0·401 |
| Var+Pla vs Var+Bup | - | - | - | 0·51 | NS | 0·115 |
| Pla+Bup vs Var+Bup | - | - | - | 0·52 | NS | 0·107 |
| Pla+Bup vs Pla+Pla | -2·166 | -28·569 | -1·288 | 0·03 | NS | 0·379 |
| **% Abstaining days, PP** | | | | | | |
|  | **Estimate** |  |  |  |  |  |
| Var+Bup vs Pla+Pla | 0·085 | 0·00002 | 0·171 | 0·02 | NS | 0·417 |
| Var+Pla vs Pla+Pla | - | - | - | 0·28 | NS | 0·219 |
| Var+Pla vs Var+Bup | - | - | - | 0·26 | NS | 0·178 |
| Pla+Bup vs Var+Bup | - | - | - | 0·73 | NS | 0·064 |
| Pla+Bup vs Pla+Pla | 0·082 | 0·0004 | 0·177 | 0·02 | NS | 0·456 |
| **Alcohol craving, PP** | | | | | | |
|  | **Estimate** |  |  |  |  |  |
| Var+Bup vs Pla+Pla | -9·4 | -19·000 | -1·2 | 0·03 | NS | 0·344 |
| Var+Pla vs Pla+Pla | -11·000 | -19·2 | -3·5 | 0·005 | Sig. | 0·431 |
| Var+Pla vs Var+Bup | - | - | - | 0·79 | NS | 0·061 |
| Pla+Bup vs Var+Bup | - | - | - | 0·48 | NS | 0·096 |
| Pla+Bup vs Pla+Pla | - | - | - | 0·10 | NS | 0·255 |

Abbreviation: Bup, bupropion; CI, confidence interval; NS, not significant; Pla=placebo; PP, per protocol for HDD, Heavy drinking days population; Sig, significant; Var, varenicline.

## **Figure S1:** **TITLE: Mean change from baseline (± SEM) of secondary outcome variable abstaining days share according to the modified intention-to-treat analysis**

##

**AbD share**

a

b

**Visit**

**Scr. R 1w 3w 5w 7w 9w 11w 13w**

**LEGEND:** (a) Mean change at the respective visit (± SEM). (b) Histograms show the mean increase (± SEM) over visits 4–8, the pre-determined, steady-state period over which statistics were performed. Significance levels and effect sizes (Cohen’s d) are presented in the Table S3. AbD=abstaining days. Bup=bupropion. Pla=placebo. SEM=standard error mean. Var=varenicline. Scr.=screening. R=randomisation. w=week.

##

## **Figure S2: TITLE: Mean change from baseline (± SEM) of secondary outcome variable alcohol craving according to the modified intention-to-treat analysis**

**Craving**

a

**Craving**

**Visit**

**Scr. R 1w 3w 5w 7w 9w 11w 13w**

b

**LEGEND:** (a) Mean change at the respective visit (± SEM). (b) Histograms show the mean decrease (± SEM) over visits 4–8, the pre-determined, steady-state period over which statistics were performed. Significance levels and effect sizes (Cohen’s d) are presented in Table S3. Bup=bupropion. Pla=placebo. SEM=standard error mean. Var=varenicline. VAS=visual analogue scale. Scr.=screening. R=randomisation. w=week.

**Figure S3: TITLE: Heat maps summarizing the uncorrected significance levels (p-values) (left) and effect sizes (Cohen’s d) (right) for primary and secondary outcome variables according to the modified intention-to-treat and per-protocol analyses**

# ****

# **LEGEND:** The proportional colour scales for p-values (left) and Cohen’s d (right) are shown. Corresponding numbers are presented in Tables 2 and 3 in the main manuscript and Tables S3 and S4 in the Appendix. Black areas=non-significant. B-PEth=phosphatidylethanol in blood. Bup=bupropion. g/day=total alcohol consumption. HDD=heavy drinking days. mITT=modified intention-to-treat. %AbD=percent abstinent days. Pla=placebo. PP=per-protocol. Var=varenicline.

**Table S5. Nausea, defined as at least one report of nausea,**

**n (%), mITT**

| **Treatment** | **No nausea**  **n (%, 95%CI)** | **Nausea**  **n (%, 95%CI)** |
| --- | --- | --- |
| Pla+Pla | 86 (88·7, 6·3) | 11 (11·3, 6·3) |
| Var+Bup | 64 (64·0, 9·4) | 36 (36·0, 9·4) |
| Var+Pla | 47 (49·0, 10·0) | 49 (51·0, 10·0) |
| Pla+Bup | 80 (87·9, 6·7) | 11 (12·1, 6·7) |
| Total | 277 (72·1, 4.5) | 107 (27·9, 4·5) |

Abbreviations: Bup, bupropion; CI, confidence interval; mITT, modified intention to treat; n, number; Pla, placebo; Var, varenicline.

**Table S6. Nausea, duration of nausea in days, in participants with at least one report of nausea,**

**n (%), mITT**

| **Treatment** | **n** | **Mean (CI)** | **Median** | **SD** |
| --- | --- | --- | --- | --- |
| Pla+Pla | 11 | 15·3 (16·4) | 10·0 | 20·7 |
| Var+Bup | 36 | 28·9 (11·6) | 16·5 | 29·6 |
| Var+Pla | 49 | 46·9 (11·4) | 45·0 | 34·4 |
| Pla+Bup | 11 | 24·0 (19·6) | 10·0 | 24·7 |
| Total | 107 | 35·2 (7·1) | 25·0 | 32·5 |

Abbreviations: Bup, bupropion; CI, confidence interval; mITT, modified intention to treat; n, number; Pla, placebo; SD, standard deviation; Var, varenicline.

# **REFERENCES**

1 de Bejczy A, Lidö H, Söderpalm B. A randomized, double-blind, placebo-controlled, multicentre trial on the efficacy of varenicline and bupropion in combination and alone for treatment of alcohol use disorder: Protocol for the COMB study. *PLoS One*. 2024; **19**: e0296118.

2 Cohen J. Statistical power analysis for the behavioral sciences Second edition. Lawrence Erlbaum Associates, Hillsdale, NJ, USA; 1988.
