# Supplementary material for: Efficacy and safety of varenicline and bupropion, in combination and alone, for alcohol use disorder: a randomized, double-blind, placebo-controlled multicentre trial
Source: Lancet Reg Health Eur. 2025 May 13;54:101310. doi: 10.1016/j.lanepe.2025.101310 (PMC12142338; doi:10.1016/j.lanepe.2025.101310)
Supplement: Renamed af0a8 [file mmc2.docx]

##### Clinical Study Protocol

| A randomized, double-blind, placebo-controlled multicenter trial on the efficacy of varenicline and bupropion in combination and alone, for treatment of alcohol use disorder | |
| --- | --- |
|  |  |
| Study Code: | COMB-BO8 |
| EudraCT Number: | 2018–000048-24 |
| Version Number: | 3.2, Lidö & deBejczy |
| Date: | 2020-06-16 |
| Sponsor: | Bo Söderpalm, Beroendekliniken, Sahlgrenska University Hospital, Västra Götalandsregion, Sweden |
| Participating Investigators: | Principal Investigator – Center 1: Bo Söderpalm, MD, PhD, Psychiatrist, Professor of Psychiatry, Beroendekliniken, Sahlgrenska University Hospital, Västra Götalandsregionen  Investigator Center 2. Markus Heilig, MD, PhD, Psychiatrist, Professor of Psychiatry, Linköping University Hospital, Region Östergötland  Investigator Center 3. Johan Franck MD, PhD, Psychiatrist, Professor of Psychiatry, Stockholm Centre for Dependency Disorders, Stockholms Läns Sjukvårdområde  Investigator Center 4. Anders C Håkansson, MD, Psychiatrist, Professor, Psychiatry Malmö, Region Skåne  Investigator Center 5. Hans Ackerot, MD., Psyhiatrist, Psychiatry Halland, Region Halland |
|  |  |
|  |  |

##### Synopsis

**Title**: A randomized double-blind placebo-controlled four-armed multicenter trial on the efficacy of varenicline and bupropion, in combination and alone, for treatment of alcohol use disorder (AUD).

**Overall Rationale**: Varenicline (Champix®) and bupropion (Zyban®, patent time expired) are approved and marketed in Europe and US for smoking cessation in nicotine use disorder, and for treatment of major depression (bupropion). There is clinical evidence of an additive effect of the drug combination of varenicline and bupropion on smoking cessation. Varenicline has been shown to reduce also alcohol intake in subjects with AUD, probably due to an effect on basal dopamine levels. It is hypothesized that bupropion will enhance the effect of varenicline and that the combined effect size will be greater than that of approved therapies for AUD. The trial uses the alcohol specific biomarker for alcohol intake, phosphatidylethanol in blood (B-PEth), as efficacy endpoint. This will be the first trial using the biomarker B-PEth as primary outcome variable. Outcome will also be measured by self-reported alcohol consumption, the standard effect measure in AUD trials. The use of a specific objective marker is expected to increase chances for detecting treatment effects.

**Primary objective**: To assess the effects of varenicline and bupropion in combination and alone for reducing alcohol consumption in individuals with AUD.

Two primary efficacy end-points will be used.

1. Alcohol consumption as measured by the objective alcohol marker B-PEth
2. Alcohol consumption as measured by heavy drinking days (HDD*) by using the Time Line Follow Back (TLFB) procedure

*HDD is defined as ≥60 grams for men and ≥40 for women according to EMA’s guideline and as ≥70 grams for men and ≥56 grams for women according to FDA’s guideline on the development of medicinal products for the treatment of alcohol dependence.

**Secondary objectives**:

1. To assess the effects of varenicline and bupropion in combination and alone for reducing alcohol consumption in individuals with AUD by using

- The indirect alcohol marker carbohydrate deficient transferrin (CDT)
- The indirect alcohol marker gamma glutamyltransferase (GGT)
- Self-reported alcohol consumption as measured by TLFB:
  - Mean grams of alcohol per day
  - Number of drinking days
  - Number of drinks per drinking day
  - Number of abstaining days

1. Total score of Alcohol Use Identification Test (AUDIT)
2. Alcohol craving as measured by a Visual Analogue Scale (VAS)
3. Nicotine use measured by the nicotine saliva marker cotinine
4. Temporal Experience of Pleasure Scale (TEPS)
5. The Continous Performance Test + Activity test (CPTA)
6. To assess the relationships between the above described outcome measures and plasma drug concentrations of bupropion and varenicline

**Study design overview**: A 13-weeks (91 days) multicenter, placebo-controlled, randomized, double-blind clinical trial with four parallel groups.

**Development phase:** II

**Number of randomized subjects**: 380 subjects with AUD. 95 subjects per treatment arm will be randomized into the study.

**Number of sites**: 5 sites in Sweden

**Inclusion criteria**

1) Signed informed consent

2) Blood alcohol level below <0.1‰ (0.1 g/L) at signing informed consent

3) 25-70 years of age at screening

4) Moderate and severe AUD according to DSM-V (meeting ≥4 out of 11 criteria)

5) B-PEth levels of ≥0.5 µmol/L at screening visit (visit 1)

6) Continuous high alcohol consumption over the last 3 months prior to screening as defined by at least 2 HDD per week on a typical week.

7) Available phone number for contact

8) Ability to speak and write in Swedish

**Exclusion criteria**

1) Total abstinence between screening and randomization visit

2) Treatment of alcohol withdrawal within 30 days of study initiation

3) Pharmacological treatment within 3 months of study initiation and during the study period that may affect alcohol consumption, including but not exclusive to, varenicline, bupropion, disulfiram, acamprosate, naltrexone, nalmefene, baclofen, topiramate, ondansetron, mirtazapine, methylphenidate, dexamphetamine, atomoxetine, pregabalin, buprenorphine and methadone

4) Non-pharmacological treatment within 3 months of study initiation and during the study

period that may affect alcohol consumption

5) Current continuous use of antidepressants, opioid analgesics, benzodiazepines, zopiclone, zolpidem, hydroxyzine, alimemazine, propiomazine or other sedatives. (The sporadic use of these compounds is accepted.)

6) Any concurrent medication that may affect the results of the trial or is considered to

compromise the safety of the participants in the trial. (See SmPCs for possible interactions.)

7) Laboratory hepatic values of >3 times the upper limit of the normal range, creatinine clearence <30 ml/min, or other clinically significant abnormalities in the screening laboratory values

8) Blood pressure ≥180/110 at screening

9) Pregnancy, breast-feeding and for premenopausal women, not using one of the contraceptive methods oral contraceptive, intrauterine contraceptive device (copper or hormonal) or subcutaneous inplant.

10) Diabetes mellitus type 1 and diabetes mellitus type 2 in need of insulin treatment

11) Any current psychiatric or somatic disorder or condition that may affect assessments or compromise participant’s safety during the trial

12) ASRS- v1.1, part A score ≥4 in the marked cut-off section

13) MADRS score ≥ 20

14) Current depression that is not mild (mild depression is accepted)

15) Suicidality

16) Current illicit drug use based on urine-toxicity test and DUDIT

17) History of delirium tremens or abstinence-induced seizures within 5 years of study initiation

18) Epilepsia or seizures other than alcohol-induced, lifetime

19) Severe sleep disturbances

20) Need of alcohol detoxification

21) Living conditions not appropriate to fulfill study requirements

22) Use of herbal drugs/tea and supplementations possibly affecting outcome or safety

23) Previous randomization in this trial or participation in another trial within 3 months of enrollment into this trial.

24) Additional factors that render the participant unable to complete the study, as judged by the investigator

**Investigational medicinal products, dosages and administration**:

There will be two separate study kits for study drug 1 and 2

*Investigational medicinal product 1 (IMP1) and dosing: Varenicline 1 mg x 2 p.o. daily*

The pharmaceutical formulation will be encapsulated tablets for oral use.

Each patient kit will contain blister packed varenicline, packed in secondary packaging.

Varenicline will be escalated from 0.5 to 2 mg daily during the first week.

Day 1-3: 0.5 mg or corresponding placebo capsules once daily

Day 4-7: 0.5 mg or corresponding placebo capsules twice daily

Day 8-91: 1 mg or corresponding placebo capsules twice daily

No dose adjustment of varenicline is allowed in the study.

*Investigational medicinal product 2 (IMP 2) and dosing: Bupropion SR 150 mg x 2 p.o. daily*

The pharmaceutical formulation will be encapsulated sustained release (SR) tablets for oral use.

Each patient kit will contain blister packed bupropion, packed in secondary packaging.

Bupropion will be escalated from 150 to 300 mg daily during the first week.

Day 1-7: 150 mg or corresponding placebo capsules once daily

Day 8-91: 150 mg or corresponding placebo capsules twice daily

No dose adjustment of bupropion is allowed in the study.

*Dosages*: The IMPs will be distributed at 7 occasions: Day 0, Day 7, Day 21, Day 35, Day 49, Day 63 and Day 77. The doses and route of administration for varenicline and bupropion are those approved and recommended as oral formulations for smoking cessation. (Bupropion is registered for major depression in two dose levels, 300 mg daily, as used in the present study and in a lower dose of 150 mg daily.)

**Double blind treatment phase (Week 1-Week 13)**

The trial comprises 9 study visits over 91 days: Screening visit, Day 0 (randomization), Day 7, Day 21, Day 35, Day 49, Day 63, Day 77 and Day 91. The total duration of the study for one participant will be 13 weeks (91 days). Week 1 is dose escalation. Steady state dose period is Week 2 - Week 13.

**Randomization**

Randomization will be carried out according to block randomization.

Eligible subjects will be randomized in a 1:1:1:1 ratio to one of the following treatments:

1. Varenicline + Bupropion SR
2. Varenicline + Placebo
3. Bupropion SR + Placebo
4. Placebo + Placebo

**Stratifiction**

No partitioning other than the intervention will be used.

**Primary outcome variables and examinations**

- Alcohol consumption as measured by blood levels (µmol/L) of the objective alcohol marker Phosphatidylethanol (B-PEth). Venous blood will be collected at every study visit.
- Alcohol consumption as measured by heavy drinking days (HDD), by using the TLFB procedure. Self-report data will be collected at every study visit.

**Study period**

Active trial period is estimated to 3 years

First-Subject-In: March 2019.

Last-Subject-Out: June 2022.

**Table 1**. Time schedule and summary of study assessments in COMB-BO8

| Accepted range |  | **Max**  **14 days** | **Max 10**  **days** | **+/- 3 days** | **+/- 3 days** | **+/- 3 days** | **+/- 3 days** | **+/- 3 days** | **+/- 3 days** | **+/- 3 days** |  |  |
| --- | --- | --- | --- | --- | --- | --- | --- | --- | --- | --- | --- | --- |
| Total number of study visits: 9 | **Ph Scr** | **Visit**  **1** | **Visit 2** | **Visit**  **3** | **Visit**  **4** | **Visit**  **5** | **Visit**  **6** | **Visit**  **7** | **Visit**  **8** | **Visit**  **9** | **Ph**  **call** | **Ph**  **call** |
| Total on-site time:12 hrs. |  | 3 hrs | 1.5 hr | 45  min | 45  min | 45  min | 1.5 hr | 45  min | 1.5 hr | 1.5 hr | 15  min | 15  min |
| Total physician time 2 (2.5*) hrs. |  | 1 hr | 30  min |  |  |  | 30*  min |  |  | 30  min |  |  |
| **Eligibility assessments** |  | Scr | Day  0 | Day 7 | Day  21 | Day 35 | Day 49 | Day  63 | Day 77 | Day 91 | FU  +7 | FU  +30 |
| Informed consent |  | **√** |  |  |  |  |  |  |  |  |  |  |
| ID Control |  | **√** |  |  |  |  |  |  |  |  |  |  |
| Breath alcohol concentration |  | **√** |  |  |  |  |  |  | **√** |  |  |  |
| Subject eligibility criteria | **√** | **√** | **√** |  |  |  |  |  |  |  |  |  |
| AUD diagnosis |  | **√** |  |  |  |  |  |  |  |  |  |  |
| Randomization |  |  | **√** |  |  |  |  |  |  |  |  |  |
| Patient trial card distribution |  |  | **√** |  |  |  |  |  |  |  |  |  |
| Patient trial card collection |  |  |  |  |  |  |  |  |  | **√** |  |  |
| Demographics |  | **√** |  |  |  |  |  |  |  |  |  |  |
| Medical history | **√** | **√** |  |  |  |  |  |  |  |  |  |  |
| Concomitant medication | **√** | **√** | **√** | **√** | **√** | **√** | **√** | **√** | **√** | **√** |  |  |
| Physical examination * |  | **√** |  |  |  |  | **√*** |  |  | **√** |  |  |
| Vital signs |  | **√** | **√** | **√** | **√** | **√** | **√** | **√** | **√** | **√** |  |  |
| ASRS |  | **√** |  |  |  |  |  |  |  |  |  |  |
| MADRS (also safety measure) |  | **√** |  |  | **√** |  |  |  |  | **√** |  |  |
| DUDIT (also safety measure) |  | **√** |  |  |  |  |  |  |  | **√** |  |  |

| **Outcome specific measurements** |  | Scr  **Visit**  **1** | Day 0  **Visit**  **2** | Day 7  **Visit**  **3** | Day 21  **Visit**  **4** | Day 35  **Visit**  **5** | Day 49  **Visit**  **6** | Day  63  **Visit**  **7** | Day 77  **Visit**  **8** | Day 91  **Visit**  **9** | FU+7 | FU+30 |
| --- | --- | --- | --- | --- | --- | --- | --- | --- | --- | --- | --- | --- |
| TLFB alcohol |  | √ | √ | √ | √ | √ | √ | √ | √ | √ | √ | √ |
| VAS Craving |  | √ | √ | √ | √ | √ | √ | √ | √ | √ |  |  |
| B-PEth |  | √ | √ | √ | √ | √ | √ | √ | √ | √ |  |  |
| CDT |  | √ | √ | √ | √ | √ | √ | √ | √ | √ |  |  |
| GGT |  | √ | √ | √ | √ | √ | √ | √ | √ | √ |  |  |
| Alcohol QF | √ | √ |  |  |  |  |  |  |  |  |  |  |
| Nicotine QF |  | √ |  |  |  |  |  |  |  |  |  |  |
| Alcohol history |  | √ |  |  |  |  |  |  |  |  |  |  |
| Nicotine history |  | √ |  |  |  |  |  |  |  |  |  |  |
| AUDIT |  | √ |  |  |  |  |  |  | √ |  |  |  |
| Cotinine |  |  | √ |  |  |  |  |  | √ |  |  |  |
| CPTA test |  | √ |  |  |  |  |  |  | √ |  |  |  |
| TEPS scale |  | √ |  |  |  |  |  |  | √ |  |  |  |

| **Safety measurements** |  | Visit 1 | Visit 2 | Visit 3 | Visit 4 | Visit 5 | Visit 6 | Visit 7 | Visit 8 | Visit 9 |  |  |
| --- | --- | --- | --- | --- | --- | --- | --- | --- | --- | --- | --- | --- |
| AE/SAE |  | √ | √ | √ | √ | √ | √ | √ | √ | √ | √ | √ |
| AST |  | √ |  |  | √ |  |  |  |  | √ |  |  |
| ALT |  | √ |  |  | √ |  |  |  |  | √ |  |  |
| PC (Prothrombin complex) |  | √ |  |  | √ |  |  |  |  | √ |  |  |
| Creatinine |  | √ |  |  | √ |  |  |  |  | √ |  |  |
| Glucose |  | √ |  |  |  |  |  |  |  | √ |  |  |
| Na+/K+ |  | √ |  |  |  |  |  |  |  | √ |  |  |
| Hb |  | √ |  |  |  |  |  |  |  | √ |  |  |
| LPK |  | √ |  |  |  |  |  |  |  | √ |  |  |
| TPK |  | √ |  |  |  |  |  |  |  | √ |  |  |
| hsCRP (also outcome measure) |  | √ |  |  |  |  |  |  |  | √ |  |  |
| U-hCG |  | √ | √ |  |  |  |  |  |  | √ |  |  |
| U-tox |  | √ | √ |  |  |  |  |  |  | √ |  |  |
| MADRS |  | √ |  |  | √ |  |  |  |  | √ |  |  |

| **Study medication distribution**  **and accountability** |  | Visit 1 | Visit 2 | Visit 3 | Visit 4 | Visit 5 | Visit 6 | Visit 7 | Visit 8 | Visit 9 | FU  +7 | FU  +30 |
| --- | --- | --- | --- | --- | --- | --- | --- | --- | --- | --- | --- | --- |
| Study medication distribution |  |  | √ | √ | √ | √ | √ | √ | √ |  |  |  |
| Drug accountability by pill count |  |  |  | √ | √ | √ | √ | √ | √ | √ |  |  |
| Drug plasma concentration BUP |  |  |  |  | √ |  | √ |  |  |  |  |  |
| Drug plasma concentration VAR |  |  |  |  | √ |  | √ |  |  |  |  |  |

**Table 1.** Study schedule of events. MD=medical doctor/physician; BL = baseline; FU = follow-up; Scr=screening; Ph= phone; DUDIT=Drug Use Disorder Identification Test; ASRS=Adult ADHD self-report scale; MADRS= Montgomery Åsberg depression rating scale; QF=quantity frequency; AUDIT=Alcohol Use Disorder Identification Test; TLFB=Time Line Follow Back; VAS=Visual analogue scale; B-PEth=Blood Phosphatidylethanol; CDT=Carbohydrate Deficient Transferrin ; GGT= gamma Glutamyltransferase; AE= Adverse Event; SAE= Serious Adverse Event; AST= Aspartate Aminotransferase; ALT= Alanine Aminotransferase; Hb= Haemoglobin; PC =Prothrombin complex; LPK= leukocyte plasma count; TPK= trombocyte particle concentration; h; U-hCG= Urine human chorionic gonadotropin; hsCRP= high sensitive C-reactive protein; U-tox= urine toxicology; CPTA= Continuous Performance Test + Activity; TEPS=Temporal Experience of Pleasure Scale. *If judged as necessary by medical doctor or study participant.

##### DECLATARION BY THE INVESTIGATOR

##### signature page

SPONSOR

|  |  |  |
| --- | --- | --- |
| Signature Sponsor |  | Date (yyyy-mm-dd) |

I will initiate, implement and take full responsibility for the clinical investigation. I also assume the role of the Sponsor and thus identify the role as Sponsor-Investigator.

| Bo Söderpalm |
| --- |
| Printed name of Sponsor |

INVESTIGATOR CENTER 1- COORDINATING CENTER- GOTHENBURG

I confirm that I have read and understood this protocol and that I will work according to the protocol. By my signature, I agree to personally supervise the conduct of this study in my affiliation and to ensure its conduct in compliance with the protocol, informed consent, IRB/EC procedures, the Declaration of Helsinki, ICH Good Clinical Practices guideline, the current EU directive Good Clinical Practice and local regulations governing the conduct of clinical studies. I will accept the monitor overseeing of the study.

|  |  |  |
| --- | --- | --- |
| Signature Principal Investigator |  | Date (yyyy-mm-dd) |

| Bo Söderpalm |
| --- |
| Printed name of Principal Investigator |

INVESTIGATOR CENTER 2- LINKÖPING

I confirm that I have read and understood this protocol and that I will work according to the protocol. By my signature, I agree to personally supervise the conduct of this study in my affiliation and to ensure its conduct in compliance with the protocol, informed consent, IRB/EC procedures, the Declaration of Helsinki, ICH Good Clinical Practices guideline, the current EU directive Good Clinical Practice and local regulations governing the conduct of clinical studies. I will accept the monitor overseeing of the study.

|  |  |  |
| --- | --- | --- |
| Signature Investigator |  | Date (yyyy-mm-dd) |

| Markus Heilig |
| --- |
| Printed name of Investigator |

INVESTIGATOR CENTER 3- STOCKHOLM

I confirm that I have read and understood this protocol and that I will work according to the protocol. By my signature, I agree to personally supervise the conduct of this study in my affiliation and to ensure its conduct in compliance with the protocol, informed consent, IRB/EC procedures, the Declaration of Helsinki, ICH Good Clinical Practices guideline, the current EU directive Good Clinical Practice and local regulations governing the conduct of clinical studies. I will accept the monitor overseeing of the study.

|  |  |  |
| --- | --- | --- |
| Signature Investigator |  | Date (yyyy-mm-dd) |

| Johan Franck |
| --- |
| Printed name of Investigator |

INVESTIGATOR CENTER 4- SKÅNE

I confirm that I have read and understood this protocol and that I will work according to the protocol. By my signature, I agree to personally supervise the conduct of this study in my affiliation and to ensure its conduct in compliance with the protocol, informed consent, IRB/EC procedures, the Declaration of Helsinki, ICH Good Clinical Practices guideline, the current EU directive Good Clinical Practice and local regulations governing the conduct of clinical studies. I will accept the monitor overseeing of the study.

|  |  |  |
| --- | --- | --- |
| Signature Investigator |  | Date (yyyy-mm-dd) |

| Anders C Håkansson |
| --- |
|  |

INVESTIGATOR CENTER 5 - HALLAND

I confirm that I have read and understood this protocol and that I will work according to the protocol. By my signature, I agree to personally supervise the conduct of this study in my affiliation and to ensure its conduct in compliance with the protocol, informed consent, IRB/EC procedures, the Declaration of Helsinki, ICH Good Clinical Practices guideline, the current EU directive Good Clinical Practice and local regulations governing the conduct of clinical studies. I will accept the monitor overseeing of the study.

|  |  |  |
| --- | --- | --- |
| Signature Investigator |  | Date (yyyy-mm-dd) |

| Hans Ackerot |
| --- |
| Printed name of Investigator |

**NON-ESSENTIAL CHANGES SINCE LAST PROTOCOL VERSION**

List of non-essential changes made in Amendment Protocol Version 3.2, 2020-06-16 compared to Version 3.1, 2019-10-25.

- Breath alcohol concentration is removed from time schedule Table 1.
- Dr. Tägnfors Ekman in Borås (Center 5) is removed as investigator.
- Dr. A.C Håkansson in Region Skåne (now Center 4) is included as investigator.
- Section 3.3: Phone call 30 days after EoT: “Follow up AE if any” is changed to “Collect/follow up AEs if any”.
- Section 3.3; Follow-up phone calls 7 and 30 days after EoT: “Record concomitant medication if any” is added
- Section 5.5: Residual capsules will be returned to the “coordinating center for destruction at the designated hospital pharmacy” is changed to “designated pharmacy for destruction”.
- Section 14 Study Timetable: Active trial period is now estimated to 3 years instead of 4 years.

Contact details

| Role | Name | Address | Phone | E-mail |
| --- | --- | --- | --- | --- |
| **Investigator Site 1**  Principal Investigator  Sponsor | Bo Söderpalm  *(MD, Psychiatrist,*  *Professor)* | Beroendekliniken SU, Blå Stråket 15, Sahlgrenska University Hospital,  Gothenburg | 076-836  85 94 | [bo.soderpalm@gu.se](mailto:Bo.soderpalm@gu.se) |
| Co-investigator  Sponsor delegate | Andrea deBejczy  *(MD, PhD)* | *-As above* | 073-593  05 01 | andrea.debejczy@gu.se |
| Clinical Trial Manager  Sponsor delegate | Helga Lidö  *(Pharm, PhD)* | *-As above* | 070-917 93 11 | [helga.lido@gu.se](mailto:Helga.lido@gu.se) |
| Clinical Trial Monitor | Christina Harthon | *-As above* | 0709670004 | ch@treputt.com |
| **Investigator Site 2**  Investigator | Markus Heilig  *(MD, Psychiatrist,*  *Professor)* | Linköping University Hospital, Entrance 27, floor 9, 58183 Linköping | 070-085  07 69 | markus.heilig@liu.se |
| **Investigator Site 3**  Investigator | Johan Franck  *(MD, Psychiatrist,*  *Professor)* | Beroendecentrum Stockholm  Friskvårdsvägen 4, floor 2  112 81 Stockholm | 073-966 07 36 | johan.franck@ki.se |
| **Investigator Site 4**  Investigator | Anders C Håkansson  *(MD; Psychiatrist,*  *Professor)* | Beroendecentrum Malmö, Södra Förstadsgatan 35, 20502 Malmö | 0703135-677 | anders_c.hakansson@med.lu.se |
| **Investigator Site 5**  Investigator | Hans Ackerot *(MD, Psychiatrist)* | Psykiatrin Halland,  Träslövsvägen, Ingång M, 43281 Varberg | 070-6680019 | Hans.ackerot@regionhalland.se |
| IMP manufacturer | Katrine Jelksäter  *Project manager* | Apotek, Produktion och Laboratorier AB (APL) | 072-1434152 | katrine.jelksater@apl.se |

##### Table of Contents

[1. Background 15](#_Toc518025805)

[1.1. Rationale for conducting this study 16](#_Toc518025806)

[1.2. Risk/Benefit evaluation 18](#_Toc518025807)

[2. Study Objectives and Endpoints 20](#_Toc518025808)

[2.1. Primary objective 20](#_Toc518025809)

[2.2. Secondary objective(s) 20](#_Toc518025810)

[3. Study Design and Procedures 20](#_Toc518025811)

[3.1. Overall study design and flow chart 20](#_Toc518025812)

[3.2. Rationale for study design 22](#_Toc518025813)

[3.3. Study Visits 22](#_Toc518025814)

[3.4. Study assessments 27](#_Toc518025815)

[4. Study Population 32](#_Toc518025816)

[4.1. Inclusion criteria 32](#_Toc518025817)

[4.2. Exclusion criteria 32](#_Toc518025818)

[4.3. Subject enrolment and randomization 33](#_Toc518025819)

[4.4. Discontinuation and withdrawal of subjects 33](#_Toc518025820)

[4.5. Premature termination of the study 34](#_Toc518025821)

[5. Study Treatments 34](#_Toc518025822)

[5.1. Identity of investigational medicinal products 34](#_Toc518025823)

[5.1.1. Doses and treatment regimens 35](#_Toc518025824)

[5.1.2. Labelling 36](#_Toc518025825)

[5.1.3. Storage and handling 37](#_Toc518025826)

[5.1.4. Drug accountability and treatment compliance 37](#_Toc518025827)

[5.1.5. Destruction 37](#_Toc518025828)

[5.2. Identity of non-investigational medicinal products 37](#_Toc518025829)

[5.3. Blinding, randomization and breaking codes 37](#_Toc518025830)

[5.4. Concomitant and prohibited medication 38](#_Toc518025831)

[6. Study Measurements and Variables 39](#_Toc518025832)

[6.1. Safety variable(s) 39](#_Toc518025833)

[6.2. Biological sampling procedures 39](#_Toc518025834)

[6.2.1. Handling, storage and destruction of biological samples 39](#_Toc518025835)

[6.2.2. Total volume of blood per patient 40](#_Toc518025836)

[6.2.3. Biobank 41](#_Toc518025837)

[7. Safety 41](#_Toc518025838)

[7.1. Reference Safety Information 41](#_Toc518025839)

[7.2. Definitions 52](#_Toc518025840)

[7.2.1. Adverse Event (AE) 52](#_Toc518025841)

[7.2.2. Adverse Drug Reaction (ADR) 53](#_Toc518025842)

[7.2.3. Serious Adverse Event (SAE) 53](#_Toc518025843)

[7.2.4. Suspected unexpected serious adverse reaction (SUSAR) 53](#_Toc518025844)

[7.3. Reporting 53](#_Toc518025845)

[7.3.1. Adverse Event (AE) 53](#_Toc518025846)

[7.3.2. Adverse Drug Reaction (ADR) 54](#_Toc518025847)

[7.3.3. Serious Adverse Event (SAE) 54](#_Toc518025848)

[7.3.4. Suspected Unexpected Serious Adverse Reaction (SUSAR) 54](#_Toc518025849)

[7.3.5. Annual Safety Report 54](#_Toc518025850)

[7.4. Procedures in case of emergency, overdose or pregnancy 54](#_Toc518025851)

[8. Statistics 55](#_Toc518025852)

[8.1. Sample size calculation 55](#_Toc518025853)

[8.2. Statistical analysis 55](#_Toc518025854)

[9. Data Management 58](#_Toc518025855)

[9.1. Recording of data 58](#_Toc518025856)

[9.1.1. Source data and source documents 58](#_Toc518025857)

[9.2. Data storage and management 59](#_Toc518025858)

[9.3. Study compliance 60](#_Toc518025859)

[10. Quality Control and Quality Assurance 60](#_Toc518025860)

[10.1. Monitoring 60](#_Toc518025861)

[10.2. Audits and inspections 60](#_Toc518025862)

[11. Ethics 60](#_Toc518025863)

[11.1. Ethics committee 61](#_Toc518025864)

[11.2. Informed consent 61](#_Toc518025865)

[11.3. Subject data protection 61](#_Toc518025866)

[11.4. Insurances 61](#_Toc518025867)

[12. Protocol Deviations and Amendments 61](#_Toc518025868)

[13. Report and publications 62](#_Toc518025869)

[14. Study Timetable 62](#_Toc518025870)

[14.1. Definition of “End of study” 62](#_Toc518025871)

[15. List of References 63](#_Toc518025872)

16. Appendix……………………………………………………………………………………………67

##### Appendices

1. Labeling text for IMP1 and IMP2 (in Swedish)
2. Emergency Card (in Swedish and English)

##### List of ATTACHMENTS

1. SmPC Champix Varenicline (in Swedish)
2. SmPC Bupropion Sandoz (in Swedish)

*Validated Swedish versions of study scales:*

1. Adult Self-Report Scale (ASRS)
2. Alcohol use disorder identification test (AUDIT)
3. Drug Use Disorder Identification Test (DUDIT)
4. Montgomery Åsberg depression rating scale (MADRS)
5. Temporal experience of pleasure scale (TEPS)
6. Time line follow back (TLFB)
7. Visual Analogue Scale for Craving (VAS Craving)

##### List of Abbreviations

| Abbreviation | Explanation |
| --- | --- |
| AE | Adverse event |
| ADR | Adverse drug reaction |
| Alcohol | Alcohol (used throughout SP) refers to ethanol |
| ASRS | Adult Self-Report Scale (Attention Deficit Hyperactivity Disorder) |
| ALT | Alanine aminotransferase |
| AST | Aspartate aminotransferase |
| AUD | Alcohol use disorder |
| AUDIT | Alcohol use disorder identification test |
| B-PEth | Phosphatidylethanol in blood |
| BrAC | Breath alcohol concentration |
| BUP | Bupropion |
| CA | Competent Authority |
| CDT | Carbohydrate deficient transferrin |
| CRA | Clinical Research Associate |
| CRF | Case Report Form |
| CPTA | Continuous Performance test + Activity |
| DSM-V | Diagnostic and statistical manual of mental disorders, 5th edition |
| DUDIT | Drug Use Disorder Identification Test |
| EoT | End of Treatment |
| Hb | Hemoglobin |
| hsCRP | High sensitive C-reactive protein |
| ICF | Informed Consent Form |
| IMP | Investigational medicinal product |
| NNT | Number needed to treat |
| DA | Dopamine |
| DUDIT | Drug disorder identification test |
| GGT | Gamma glutamyl transferase |
| LPK | Leukocyte plasma count |
| MADRS | Montgomery Åsberg depression rating scale |
| MD | Medical Doctor |
| nAChRs | Nicotinic acethylcholine receptors |
| PI | Principal Investigator |
| PC | Prothrombin complex |
| SAE | Serious adverse event |
| SmPC | Summary of Product Characteristics |
| SR bupropion | Sustained release bupropion |
| SUSAR | Suspected Unexpected Serious Drug Reaction |
| TEPS | Temporal experience of pleasure scale |
| TPK | Thrombocyte particle concentration |
| TLFB | Time line follow back |
| U-hCG | Urine human chorionic gonadotropin |
| U-tox | Urine Toxicology |
| VAR | Varenicline |
| VAS | Visual analogue scale |
| QF | Quantity frequency |

# Background

Alcohol is among the top contributors to global burden of disease (WHO, 2018) and the lifetime expectancy of individuals with alcohol use disorder (AUD) is estimated to be shortened by 23 years (Lesch et al., 1986). Four medications are available - disulfiram, acamprosate, naltrexone and nalmefene, of which the latter is non-reimbursed. Disulfiram has a good effect short-term, but only for patients accepting the abstinence goal, since its effect builds on the aversive and potentially harmful somatic reactions due to acetaldehyde intoxication. The other three drugs act by reducing alcohol’s reinforcing effects and/or craving for the drug and can be used for both the abstinence and harm reduction goals (Socialstyrelsen, 2010). The effect sizes are, however, small (Cohen’s d = 0.2) (Rosner et al., 2010;Soyka and Rosner, 2010;Jonas et al., 2014). European general practitioners, psychiatrists and internists prescribe these drugs in small amounts and the treatment gap has been estimated to over 90% (Kohn et al., 2004) maybe because numbers needed to treat (NNT) of 8-14 are perceived as useless. New treatment options with larger effect sizes and lower NNTs would have a better chance to gain acceptance among practitioners and be in fact used for the benefit of this much at risk population.

This trial (COMB-BO8) will assess the combination of two established and well-tolerated medications, varenicline (VAR) and bupropion (BUP) on a new indication, i.e. AUD. The trial is funded mainly by the Swedish Research Council under the call Clinical Therapy Research. A previous long-term basic research program financed i.a. by the Swedish Research Council ended up in knowledge indicating that VAR and BUP may be suitable to repurpose for AUD. According to ClinicalTrials.gov there is currently no ongoing study similar to the present trial.

Varenicline (VAR) is a registered smoking cessation medication that by its partial agonistic effect at brain nicotinic acetylcholine receptors (nAChRs) slightly elevates brain dopamine (DA) levels. VAR has been shown to reduce alcohol intake in two randomized controlled trials (RCTs). One was performed by the Sponsor/PI with the Swedish Network for Treatment Trials in the Addiction Field (de Bejczy et al., 2015) and one was performed in the US, in a National Institute on Alcohol Abuse and Alcoholism (NIAAA) program for repurposing existent drugs for use in the treatment of AUD (Litten et al., 2013). The effect size was approx. 0.35 (Cohen’s d) in the Swedish study and 0.4 (Cohen’s d) in the American study. If confirmed in further trials this compound may thus outperform the drugs currently available for treatment of AUD.

Bupropion (BUP) is a centrally active, weak norepinephrine-dopamine reuptake inhibitor (NDRI) with minimal affinity to the serotonin transporter (Jorenby et al., 1999). It was registered for major depression in the 1980s (Voxra®, patent time expired), and based on clinical observations and a series of positive RCTs, BUP received approval as smoking cessation medication in 1997 (Zyban®, patent time expired). BUP is also being used off-label as treatment for attention deficit hyperactivity disorder (Kornfield et al., 2013) and the combination bupropion and naltrexone (Mysimba®) was registered September 2014 for the treatment of obesity. The hypothesis of the present study is that BUP will show a similar effect size as VAR for AUD, and that an effect size of 0.5-0.6 (Cohen’s d) might be reached by combining VAR and BUP.

B-PEth is a validated biomarker for alcohol consumption and has superior sensitivity and specificity (theoretically 100%) compared to CDT and GGT (See Section 3.4 Study Assessments). It has been shown that B-PEth has high enough fidelity in AUD studies to pick up a treatment effect that CDT, GGT and self-report in a diary fail to disclose (de Bejczy et al., 2015). This will be the first study using the biomarker B-PEth for alcohol intake as primary outcome variable. The aim of using a specific objective marker is to increase chances for detecting treatment effects and pave the way for future study guidelines in AUD. Self-reported heavy drinking days, the conventinal effect measure for clinical trials in AUD, will be used as a separate primary outcome variable.

## Rationale for conducting this study

Theoretical rationale for conducting this study involve:

*Alcohol’s interference with the dopaminergic brain reward system***.** The rewarding and reinforcing effects of alcohol involve glycine receptors in the nucleus accumbens (nAc) and nAChRs in the midbrain, resulting in increased neuronal firing of mesolimbic dopamine DA neurons and eventually DA release in the nAc (ventral striatum), for reviews, see (Soderpalm et al., 2009;Soderpalm and Ericson, 2013;Soderpalm et al., 2017). This insight sparked animal studies (Steensland et al., 2007), human experimental studies (McKee et al., 2009) and so far two RCTs on the concept of using VAR for AUD (Litten et al., 2013;de Bejczy et al., 2015). This compound slightly raises DA levels in nAc and simultaneously prevents further DA activation by nAChR stimulation (Einhorn et al., 1988). VAR is available for smoking cessation (Cahill et al., 2012) and has been shown to be effective also for reducing alcohol intake in AUD regardless of the afflicted being nicotine user or not (Litten et al., 2013;de Bejczy et al., 2015). BUP also interferes with DA transmission in nAc and may be expected to increase the DA releasing effect of VAR when combined with this medication (see below).

*The DA deficiency theory of substance addiction (Koob and Volkow, 2016).* Human studies indicate that both pre- and postsynaptic aspects of basal DA neurotransmission are reduced in AUD (Heinz et al., 2005;Volkow et al., 2007). Further, signs of reduced DA neurotransmission have been associated with increased alcohol intake and increased craving and alcohol-cue reactivity, in animal (Weiss et al., 1996;Feltmann et al., 2016) and human studies (Heinz et al., 2004), respectively. In other words, the compromised DA system in AUD, which may be genetically determined and/or develop as an adaptation to chronic alcohol (Volkow et al., 2006), may drive alcohol intake. This suggests that by increasing DA levels alcohol intake may be reduced. The effects of VAR on AUD now observed in two RCTs may derive from the DA elevating effect and as such represent proof-of-principle that elevating central DA levels reduces alcohol intake. Further support for this contention comes from a study where the opposite was tried, i.e. chronic i.m. administration of flupentixole, a neuroleptic blocking postsynaptic DA D2 receptors. This treatment worsened the condition by producing earlier and heavier relapses to drinking, as compared to placebo (Wiesbeck et al., 2001).

*The evidence of an additive effect of VAR and BUP.* The combined use of VAR and BUP has been investigated for smoking cessation with results showing that the combined treatment is superior to VAR monotherapy, with odds ratios for abstinence rate up to 3.51 (Rose and Behm, 2014;Hall et al., 2015;Vogeler et al., 2016;Rose and Behm, 2017). Given the DA hypothesis of AUD outlined above, a superiority of the combination therapy in the treatment of AUD is assumed. This combination is also attractive for other theoretical reasons. BUP inhibits reuptake of the catecholamines DA and norepinephrine (NA) from the synaptic cleft, thus raising also extracellular NA levels in the brain. It has been reported that drugs raising endogenous NA levels may reduce alcohol consumption (Wilens et al., 2008;de Bejczy and Soderpalm, 2015). Recent preclinical data show that VAR and BUP produce an additive effect on DA release in the rat ventral striatum and that the combination abolishes the alcohol deprivation effect in alcohol high-consuming rats (Fig. 1a, b). The latter measure is considered to have a high predictive value for effects in humans (Spanagel and Holter, 2000). Both drugs have been found effective and safe across various populations and are recommended in patients with psychiatric or addictive comorbidity (Aubin et al., 2011) despite their interaction with DA mechanisms.


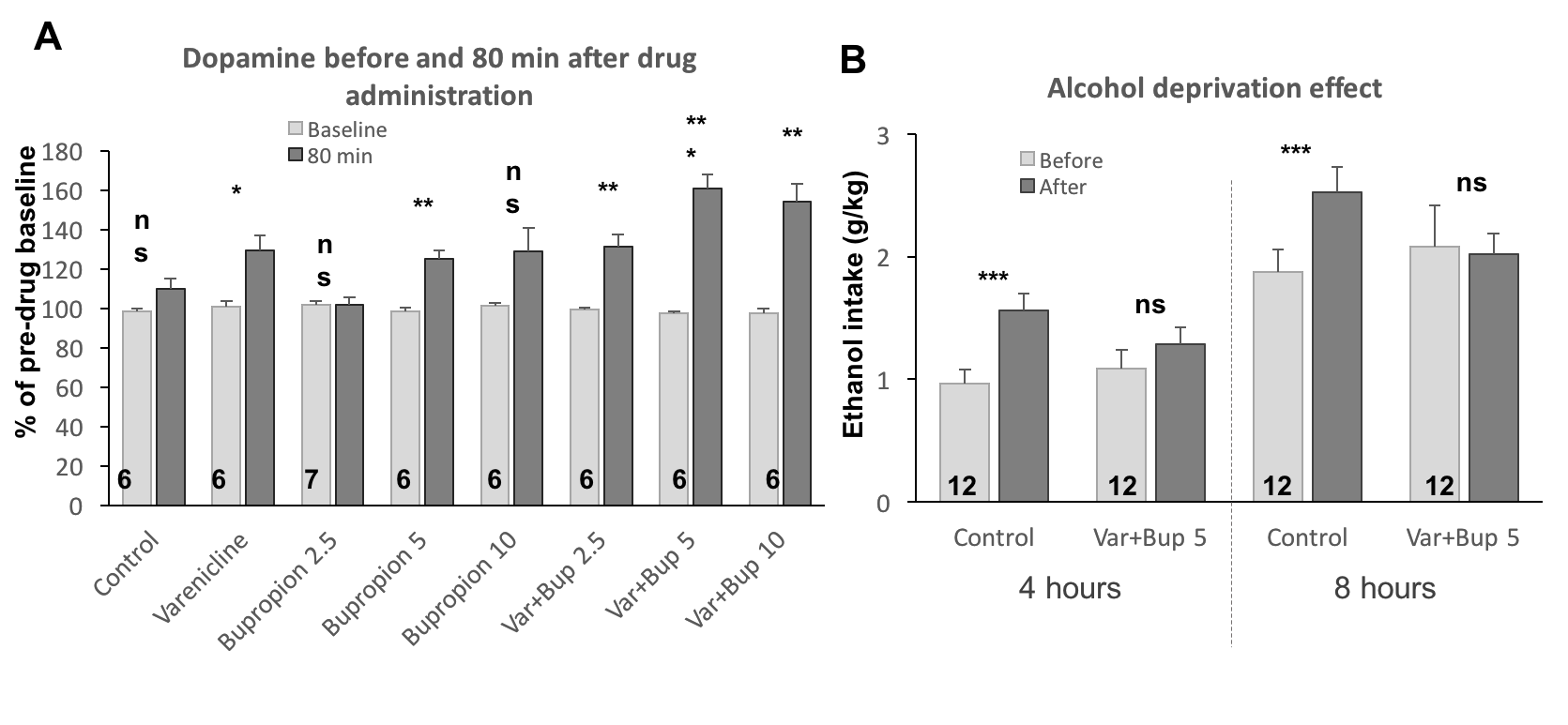


**Figure 1**. Effects of VAR and BUP alone and in combination on DA levels in rat nucleus accumbens (A), and effect of the combination on the alcohol deprivation effect (B). The BUP doses 2.5, 5.0 and 10.0 indicate mg drug/kg rat weight administered i.p., and the dose of VAR was 1.5 mg/kg.

*The evidence of the importance of combating both alcohol and nicotine intake in co-abusers***.** Nicotine use, mainly in the form of smoking, is significantly more common among individuals with AUDs (Bien and Burge, 1990), and the severity of nicotine dependence is associated with higher craving in alcohol dependent patients (Hillemacher et al., 2006). This co-abuse imposes severe health problems. There is animal evidence indicating that nicotine administration as such by a pharmacological action increases alcohol intake (Blomqvist et al., 1996) (Potthoff et al., 1983) (Smith and Kieval, 2000). Therefore treatment effects on nicotine intake could potentially reduce also alcohol consumption (Prochaska et al., 2004). Also for this reason it is of considerable interest to explore the impact of smoking cessation treatments on alcohol consumption in individuals with AUD, as well as whether the expected effect is related or unrelated to the reduction of nicotine intake.

*B-PEth is superior to other biomarkers and self-report for detecting real treatment effects in alcohol trials* (de Bejczy et al., 2015;Walther et al., 2015). At present time, the established standard by authorities for efficacy measurement in studies of AUD is abstinence days and proportion of heavy drinking days; self-report measures often collected by the The Time-Line-Follow-Back (TLFB) method. The reliability of self-report may be questioned, but the assumption has been that mis-report is similar across treatment groups and that the fault imposed therefore is equal and part of the noise in the data set. The study comparing placebo and VAR for the treatment of AUD, that lead to the present trial, used self-report in a diary but included also Carbohydrate Deficient Transferinn (CDT), Gamma-Glutamyl Transferase (GGT) and B-PEth in order to estimate alcohol consumption (de Bejczy et al., 2015;Walther et al., 2015). Here, B-PEth was the marker correlating the best to self-report and the best correlation was found between the most specific markers of alcohol intake, i.e. B-PEth and CDT. Interestingly, the self-report in the VAR group correlated better to all biomarkers than that in the placebo group. Further, if the study had used self-report only, the VAR effect would not have been revealed (effect size Cohen’s d 0.35 on B-PEth levels). Also, when using B-PEth as outcome variable the large placebo response observed in the self-report data disappeared. These data indicate that B-PEth is the most sensitive biomarker and has high enough fidelity in AUD studies to pick up a true treatment effect that CDT, GGT and self-report in a diary fail to disclose. Based on this, the Sponsor’s judgement is that self-report is too blunt of a measure that will overestimate placebo effects and thereby underestimate true treatment effects, and that as of today B-PEth levels should be used as the primary effect variable in AUD treatment studies.

## Risk-Benefit Evaluation

This section gives risk-benefit evaluation for the conductance of the trial and for the use of VAR in the dose of 1 mg x 2 daily and BUP in the dose of 150 mg x 2 daily, in combination and alone.

**Clinical and ethical standards**

To give the best conditions possible for the safety of the study subject, the trial will be conducted in compliance with the ethical principles that have their origin in the Declaration of Helsinki and with the ICH Good Clinical Practice (GCP) guidelines.

**Anticipated clinical benefits with VAR and BUP and B-PEth as used in the study**

- The main benefit of the present study would be showing a superior treatment effect of the combination of drugs VAR and BUP, compared to VAR alone in AUD, which would be a first time finding. Further, if the effect size is 0.5-0.6 (Cohen’s d), as hypothesized, this would represent the largest effect size observed for any available treatment of AUD.
- A second benefit would be confirming the treatment effect of VAR on alcohol consumption in AUD patients, which would represent the third study showing this effect.
- A third benefit would be showing a significant treatment effect of BUP on alcohol consumption in AUD patients, which would be a first time ever finding.
- A fourth benefit would be to demonstrate the superiority of B-PEth over self-report (in this case TLFB) for disclosing treatment effects in a second alcohol treatment trial. Such a finding may contribute to changing and improving the standards regarding primary outcome variables in alcohol treatment trials.
- A fifth benefit would be for the health care. Assessing the utility of B-PEth as biomarker will help clinicians screen for alcohol problems in a reliable fashion.

**Health economic benefits**

Consequences of high alcohol consumption impose tremendous suffering to the afflicted as well as burden on health and social care systems, and the annual costs to Swedish society have been estimated to at least 26 billion crowns (Socialstyrelsen, 2010). It is estimated that alcohol sequelae, i.e. consequences of alcohol, reduce life expectance by 23 years (Lesch et al., 1986).The life-time risk of dying from alcohol-related causes (accidents and diseases) increases exponentially with alcohol consumption (Rehm, 2011). Not only abstinence but also reduction of alcohol intake profoundly lowers the risk of death in the heaviest consumers (Rehm and Roerecke, 2013). Many patients with AUD are not motivated for the abstinence goal but can accept the low risk drinking level goal (Hodgins et al., 1997;Heather et al., 2010). Focus has lately shifted from the abstinence goal in alcohol treatment trials to a goal of reducing alcohol consumption to lower risk levels (Mann et al., 2013;Nutt and Rehm, 2014). The present combination treatment, with expected effect size as described above, could lead to huge health economic benefits.

**General benefits for the individual**

By participating in the trial, the subject will:

- Get access to tentative new pharmacological treatments for AUD
- Help support medical science and help the development of treatments for AUD that can benefit people in the future
- Improve his/her own knowledge of the risks with excessive alcohol consumption and the nature of the disease
- Receive health status checked at no cost

**Anticipated adverse drug reactions in the study**

VAR and BUP are established and well-used prescription medication on the European and the US markets. The risks versus benefits regarding their approved indications nicotine dependence have previously been evaluated and are continuously being under evaluation. For all **Reference safety information** and full list on anticipated adverse drug reactions, go to Safety Section 7.1 and the SmPCs for VAR and BUP (Attachment 1 and 2).

**Risk-Benefit Conclusion**

Based on the clinical data on VAR and BUP in combination in the reference studies 1-7 (see Reference Safety Information, Section 7.1), the main expected AEs with VAR and BUP used in combination are:

- Sleep disturbance/insomina
- Nausea
- Dizziness
- Vivid dreams

Based on the clinical data on VAR and BUP in combination in the reference studies 1-7 (see Reference Safety Information, Section 7.1), the main expected medical concerns are:

- An increased risk of seizures
- An increased risk of acute hypertension

**Risk Benefit Conclusion**

Based on the anticipated benefits of conducting the study as stated above, and the Reference Safety Information in Section 7.1, the Sponsor’s judgment of the risk/benefit balance is that the benefits of conducting the trial justify the risks associated with the trial. The studies described above and clinical experience point to a good safety and tolerability profile of VAR and BUP, when used alone and when used in combination. Considering the frequent study visits and the exclusion of at-risk subjects for experiencing seizures we believe that the risks associated seizures will be mitigated or minimized. We judge that depressive side effects of VAR (previous a black box warning but not anymore) will be minimized by exclusion of depressive subjects and close follow-up. It is the Sponsor’s judgment that the individual subject will benefit from the treatments and that the potential use of the study by far exceeds its tentative risks. Apart from the medical and psychosocial benefits for each alcohol dependent individual, a reduction in alcohol – and/or nicotine consumption may produce huge socioeconomic benefits to society in general, enhancing the significance of this trial. The Sponor’s conclusion is that VAR and BUP will be safe and well tolerated in the present trial and states that the anticipated benefits justify the risks.

# STudy Objectives and Endpoints

## Primary objective

To assess the effects of VAR and BUP in combination and alone for reducing alcohol consumption in individuals with AUD.

Two primary efficacy end-points will be used.

1. Alcohol consumption as measured by the objective alcohol marker B-PEth
2. Alcohol consumption as measured by heavy drinking days (HDD*) by using the Time Line Follow Back (TLFB) procedure

*HDD is defined as ≥60 grams for men and ≥40 for women according to EMA’s guideline and as ≥70 grams for men and ≥56 grams for women according to FDA’s guideline on the development of medicinal products for the treatment of alcohol dependence.

## Secondary objective(s)

1. To assess the effects of varenicline and bupropion in combination and alone for reducing alcohol consumption in individuals with AUD by using

- The indirect alcohol marker carbohydrate deficient transferrin (CDT)
- The indirect alcohol marker gamma glutamyltransferase (GGT)
- Self-reported alcohol consumption as measured by TLFB:
  - Mean grams of alcohol per day
  - Number of drinking days
  - Number of drinks per drinking days
  - Number of abstaining days

1. Total score of Alcohol Use Identification Test (AUDIT)
2. Alcohol craving as measured by a Visual Analogue Scale (VAS)
3. Nicotine use measured by the nicotine saliva marker cotinine
4. Temporal Experience of Pleasure Scale (TEPS)
5. The Continous Performance Test + Activity test (CPTA)
6. To assess the relationships between the above described outcome measures and plasma drug concentrations of bupropion and varenicline (measured at visits 4 and 6)

# Study Design and Procedures

## Overall study design and flow chart

Design: The COMB-BO8 study is a Phase II, randomized, multicentre, double-blind, placebo-controlled trial with 4 parallel groups.

**
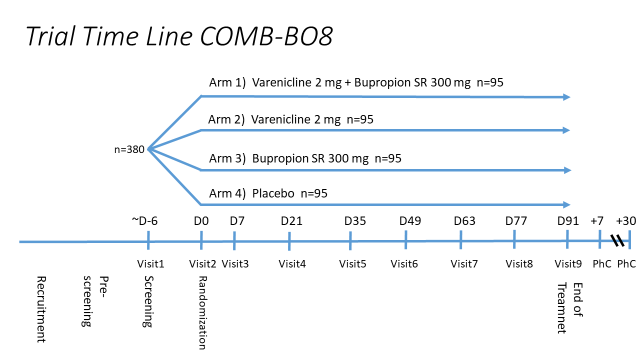
**

**Figure 2**. Schematic time line for study visits. The study comprises a double-blind phase with 9 visits in total over

13 weeks (91 days). Day 0-7 is titration of dosing.

The required time for study participation is shown in table 2.

| **Visits Day Estimated time for visits** | | | |
| --- | --- | --- | --- |
| Visit 1 | Baseline | Medical visit/ Scr | 3hrs |
| Visit 2 | Day 0 | Medical visit/Rand | 1.5 hr |
| Visit 3 | Day 7 | Core battery visit | 45 min |
| Visit 4 | Day 21 | Core battery visit | 45 min |
| Visit 5 | Day 35 | Core battery visit | 45 min |
| Visit 6 | Day 49 | Medical visit | 1.5 hr |
| Visit 7 | Day 63 | Core battery visit | 45 min |
| Visit 8 | Day 77 | Core battery visit | 1.5 hr |
| Visit 9 | Day 91 | Medical visit/EoT | 1.5 hr |
| All | - | Total time at Center | 12 hrs |

**Table 2**. Summary of time required for the study participant.

Follow-up phone calls are scheduled at Day 7 and Day 30 after End-of-Treatment

Phone call Day+ 7: 15 min.

Phone call Day+ 30: 15 min

The total time required from the trial subject, including the phone calls, is 12.5 hrs.

## Rationale for study design

This trial assesses the efficacy of the established smoking cessation agensts, VAR and BUP, on the new indication, AUD. The study assesses VAR and BUP alone and in combination, and is designed with 4 parallel groups to address the following hypotheses:

- Combination treatment with VAR and BUP reduces B-PEth levels and HDD from baseline and significantly more than placebo. The effect size, as measured by B-PEth, for the combined treatment is hypothesized to be 0.5-0.6 (Cohen’s d).
- BUP reduces B-PEth levels from baseline and HDD from baseline and significantly more than placebo. The effect size, as measured by B-PEth, is hypothesized to be approximately 0.35 (Cohen’s d).
- VAR reduces B-PEth levels from baseline and HDD significantly more than placebo. The effect size, as measured by B-PEth, is expected to be approximately 0.35 (Cohen’s d).

**Rationale for two primary End Points**

Regulatory agencies will consider only one specific primary objective. The two primary endpoints fulfill regulatory requests independently and separately. HDD fulfill requirements for application of marketing approval. The biological marker is used to assess the reliability of PEth as End Point measure.

**Rationale for the study design**

A parallel group design will be used. A crossover design is not an option due to the risk that the beneficial effects of the first treatment will carry over into the second treatment period. The duration of a parallel-group trial is often shorter because only one treatment period is involved (yet a larger study group is needed). As in deBejczy el al. (de Bejczy et al., 2015) and Litten et al.(Litten et al., 2013) it is estimated that 12 weeks steady state duration will be adequate for detecting tentative effects, as measured by B-PEth and HDD, and at the same time be manageable for the participants, thus optimizing study compliance.

## Study Visits

A full summary of the study visits in time is given in Table 1 in the Synopsis.

**Recruitment**

There are two modes of recruitment:

- Recruitment by advertising

Potential participants will be reached through a media campaign consisting of announcements in newspapers and/or an e-recruitment approach (a custom-made web-campaign outreach).

- Recruitment within the psychiatric/dependency clinics at the different study centers.

**Pre-screening**

There are two modes of pre-screening:

- In the advertisement, either web-based or in newspaper, the subjects will be asked to call the nearest study center for a telephone interview with staff trained to collect information about current drug and alcohol use pattern, current treatment regimens, demographics and health status. If eligibility criteria are met, an in-person screening visit is scheduled.
- Dissemination of information to interested participants will be done by medical staff at the study center clinics. Interested and eligible study subjects will undergo 30-minute telephone or face-to-face interview with trained staff to collect information about their current drug and alcohol use pattern, current treatment regimens, demographics and health status. If eligibility criteria are met, an in-person screening visit is scheduled.

**Visit 1. Screening Visit. Accepted range: Maximum 14 days from prescreening**

ID control is performed and breath alcohol level is determined to ensure sobriety (defined as blood conc. <0.1‰). The participant may be offered a new screening visit, e.g. the following day, if sobriety is not fulfilled. Before any trial-related examination is performed, informed consent will be obtained. The subject will be informed orally of the aim of the trial, what participation entails and will be supplied with written information summarizing the details of the trial to read. Ample time will be given to the subjects to consider their participation. Agreement to participate in the trial will be confirmed by the subject signing and dating the Informed Consent Form (ICF). Documentation of the procedure is confirmed by delegated site personnel signing and dating the ICF. Medical history and alcohol anamnesis are collected and a physical examination is performed. Vital signs, safety and outcome specific blood samples and urine samples for pregnancy and toxicology will be collected. Alcohol consumption 30 days back in time will be documented by using the TLFB procedure. Baseline information is collected; demographics, alcohol and nicotine history, depression, and ADHD. The AUDIT and DUDIT will be administered. The Temporal Experience of Pleasure scale (TEPS) will be administered, and the Continuous Performance Test + Activity (CPTA) attention test will be performed via iPod. The eligibility criteria (except B-Peth criteria) will be evaluated by the medical doctor. If non-eligible and if required by medical condition the study seeking subject may be referred to appropriate clinic. If eligibility criteria are fullfilled, the subjects will be invited to a second visit within 8 days for randomization.

- Perform BrAC test by alcometer measuring alcohol concentration in exhaled air.
- ID control
- Perform the informed consent procedure, written and orally, confirmed by the subject signing and dating the ICF.
- Collection of medical history
- Collection of alcohol anamnesis and confirmation of alcohol diagnosis.
- Complete TLFB Alcohol calendar for 30 days preceding screening visit
- Collect AEs, if any
- Collect concomitant medication, if any
- Perform physical examination, which includes examination of armpits (lymph nodes), examination of mouth and throat, auscultation of heart and lungs, and palpitation of abdomen.
- Collect vital signs: Blood presssure and heart rate (after 10 min rest), length (at visit 1 only) and weight (at visit 1 and visit 9)
- Draw blood for safety parameters, AST, ALT, PC, glucose, creatinine, Na+/K+, Hb, LPK, TPK and hsCRP
- Draw blood for efficacy paramters B-PEth, CDT and GGT
- Perform dip stick tests for U-tox and U-hCG (pregnancy status, females only)
- Collect demographic data
- Complete questionairres: VAS craving, AUDIT, DUDIT, ASRS, MADRS, Alcohol quantity frequency,nicotine quantity frequency and TEPS
- Administer CPTA test via iPod.
- Control and confirm (except B-Peth criteria) eligibility

**Visit 2. Randomization Visit.** $\mathbf{Accepted range:Maximum}$**10 days from screening visit**

The eligibility criteria based on blood sample analysis are checked and signed by medical doctor. If non-eligible and if required by medical condition the study seeking subject may be referred to appropriate clinic. The eligibility criteria are again confirmed. Medical status and concomitant medication is checked if case status has changed since screening. Data on alcohol consumption since last visit is collected by TLFB. Vital signs and blood samples for outcome specific measurements are collected. Urine sample for pregnancy and toxicology are collected. The subject is randomized to the trial and patient emergency card is handed out. Saliva sample is collected for cotinine (nicotine metabolite) determination. The IMP is distributed according to randomization list. Patient emergency card is handed out. The study participants will be instructed to start taking the study drug on the following morning, the day after the randomization visit.

*Before randomization*

- Perform dip stick tests for U-tox, and pregnancy status (if applicable)
- Collect vital signs: Blood pressure, heart rate (after 10 min rest)
- Complete TLFB alcohol calendar since last visit
- Administer VAS craving questionairre
- Check and confirm that medical status is unchanged since screening visit
- Check and confirm eligibility criteria in eCRF system (in order to do this, all above measures must be entered in eCRF)
- Randomization
- Distribute patient emergency card
- Collect concomitant medications if any
- Collect AEs if any
- IMP distribution
- Perfrom saliva dip stick test for cotinine determination
- Collect blood for B-PEth, CDT and GGT analyses

**Visit 3. Core battery visit.** $\mathbf{Accepted range}$**+/- 3 days from scheduled visit date**

AEs, data on concomitant medication, vital signs, and TLFB (data on alcohol consumption since last visit) are collected. Blood samples for alcohol specific outcome measures are drawn. IMP distribution.

*Core battery*

- Complete TLFB alcohol calendar since last visit
- Administer VAS craving questionairre
- Collect vital signs
- Collect AEs if any
- Record concomitant medication if any
- Collect blood for laboratory tests: B-PEth, CDT and GGT analyses
- IMP distribution, collection of IMP kits from last visit and drug accountability by pill count

**Visit 4. Core battery and Safety visit**$\boldsymbol{.}\mathbf{Accepted range}$**+/- 3 days from scheduled visit date**

AEs, concomitant medication, vital signs, and TLFB (data on alcohol consumption) are collected. Blood samples for alcohol specific outcome measures are drawn. Blood sampling for determination of IMP concentrations in blood will be performed before any dose is taken that day. The morning IMP dose will be given on site. The investigator will note in the CRF the time of the evening dose the day before was taken.

*Core battery*

- Complete TLFB alcohol calendar since last visit
- Administer VAS craving questionairre
- Collect vital signs
- Collect AEs if any
- Record concomitant medication if any
- Collect blood for laboratory tests: B-PEth, CDT and GGT analyses
- IMP distribution, collection of IMP kits from last visit and drug accountability by pill count.

*Additional assessments*

- Collect blood for safety tests: AST, ALT, PC and creatinine (Draw 10 ml blood)
- Amdinister MADRS (safety end point)
- Before the IMP dose is taken that day, collect blood for determination of VAR and BUP plasma concentrations (Draw 10 ml blood). The morning IMP dose will then be administered on site.

**Visit 5. Core battery visit.**$\mathbf{Accepted range}$**+/- 3 days from scheduled visit date**

Adverse events, concomitant medication, vital signs, and TLFB (data on alcohol consumption) are collected. Blood samples for alcohol specific outcome measures are drawn. IMP distribution. Estimated time for visit is 1 hr.

*Core battery*

- Complete TLFB alcohol calendar since last visit
- Administer VAS craving questionairre
- Collect vital signs
- Collect AEs if any
- Record concomitant medication if any
- Collect blood for laboratory tests: B-PEth, CDT and GGT analyses
- IMP distribution, collection of IMP kits from last visit and drug accountability by pill count.

**Visit 6. Optional Medical Visit**$\mathbf{Accepted range}$**+/- 3 days from scheduled visit date**

If judged as necessary by medical doctor or study participant, the subject is scheduled for a physician visit. Adverse events, concomitant medication, vital signs and TLFB (data on alcohol and nicotine consumption) are collected. Blood samples for alcohol specific outcome measures will be drawn. Blood sampling for determination of IMP concentrations in blood will be performed at Visit 6 before any dose is taken that day. The morning IMP dose will be given on site. The investigator will note in the CRF the time of when the evening dose the day before was taken. IMP distribution.

*Core battery*

- Complete TLFB alcohol calendar since last visit
- Administer VAS craving questionairre
- Collect vital signs
- Collect AEs if any
- Record concomitant medication if any
- Collect blood for laboratory tests: B-PEth, CDT and GGT analyses (Draw 15 ml blood)
- IMP distribution, accountability and check the returned medication kit from the last visit
- Consultation with medical doctor if requested

*Additional assessment*

- Before the IMP dose is taken that day, collect blood for determination of VAR and BUP plasma concentrations (Draw 10ml blood). The morning IMP dose will then be administered on site.

**Visit 7. Core battery visit.** $\mathbf{Accepted range}$**+/- 3 days from scheduled visit date**

Adverse events, concomitant medication, vital signs, and TLFB are collected. Blood samples for alcohol specific outcome measures are drawn. IMP distribution.

*Core battery*

- Complete TLFB alcohol calendar since last visit
- Administer VAS craving questionairre
- Collect vital signs
- Collect AEs if any
- Record concomitant medication if any
- Collect blood for laboratory tests: B-PEth, CDT and GGT analyses (Draw 15 ml blood)
- IMP distribution, collection of IMP kits from last visit and drug accountability by pill count

**Visit 8.** $\mathbf{Accepted range}$**+/- 3 days from scheduled visit date**

Adverse events, BrAC, concomitant medication, vital signs and TLFB (data on alcohol consumption) are collected. Blood samples for alcohol specific outcome measures are drawn. IMP distribution. Saliva sample is collected for cotinine determination. BrAC is determined. The TEPS will be administered and the CPTA test will be administered via iPod. AUDIT is administered.

*Core battery*

- Complete TLFB alcohol calendar since last visit
- Administer VAS craving questionairre
- Collect vital signs
- Collect AEs if any
- Record concomitant medication if any
- Collect blood for laboratory tests: B-PEth, CDT and GGT analyses (Draw 15 ml blood)
- IMP distribution, collection of IMP kits from last visit and drug accountability by pill count

*End of Analysis assessments*

- Adminsiter CPTA test via iPod
- Administer TEPS questionnaire
- Complete AUDIT
- Perform saliva dip stick test for cotinine analysis

**Visit 9. End of Treatment/Medical visit.** $\mathbf{Accepted range}$**+/- 3 days from scheduels visit date**

Medical examination by physician (30 min). AEs are collected as well as concomitant medication, vital signs and TLFB (data on alcohol consumption). Blood samples for alcohol specific outcome measures are drawn.

*Core battery*

- Complete TLFB alcohol calendar since last visit
- Administer VAS craving questionairre
- Collect vital signs
- Collect AEs if any
- Record concomitant medication if any
- Collect blood for laboratory tests: B-PEth, CDT and GGT analyses (Draw 15 ml blood)
- Collection of IMP kits from last visit and drug accountability by pill count

*End of Treatment and safety assessments*

- Patient trial card collection
- Perform physical examination, which includes examination of armpits (lymph nodes), examination of mouth and throat, auscultation of heart and lungs, and palpitation of abdomen.
- Collect vital signs: Blood pressure, heart rate after 10 min rest, and weight (at visit 1 and visit 9)
- Draw blood for safety parameters, AST, ALT, PK, glucose, PK, creatinine, Na+/K+, Hb, LPK, TPK and hsCRP
- Perform drug of abuse test (U-tox)
- Administration of MADRS (safety endpoint)
- Complete DUDIT questionnaire (safety endpoint)

**Follow-up Phone call (7 days after EoT)**

Estimated time for phone call is 15 min.

- Collect/follow up AEs if any
- Record concomitant mediation if any
- Complete TLFB alcohol calendar data

**Follow-up Phone call (30 days after EoT)**

Estimated time for phone call is 15 min.

- Collect/follow up AEs if any
- Record concomitant mediation if any
- Complete TLFB alcohol calendar data for 7 days back in time

**Extra study visits**

If judged as required by the investigator, an extra study visit may be scheduled in addition to the ordinary study visits. The date, the reason for and the action taken at the visit must be documented in the CRF in a similar manner as for the pre-scheduled study visits. Additional blood (20 ml) will be drawn id judged as necessary.

**Premature discontinuation**

In the case of premature study discontinuation, an End-of-Treatment visit will be performed. In addition, any otcome measures at the ordinary visit that are not covered by the End-of- treatment visit will be collected (applicable for visit 4,6 and 8) . Discontinued subjects will also complete an AE report by phone call Day 7+ and, if AEs persist, at Day +30 (see Section 4.4).

## Study assessments

A summary of the study assessments in time is given in Table 1 in the Synopsis.

**Investigator’s training**

The investigators and delegated qualified personnel will be trained in the study procedures, assessments and administration of scales required during the study. The training will be documented by a training certificate.

The required scales and procedures for training are:

- Phone pre-screening
- The informed consent procedure
- Structured diagnostic interviewing
- The TLFB Alcohol procedure including interviewing technique
- Collection of AE and reporting of SAE
- Administration of study scales
- Handling of study specific medical device
- CPTA test administration on electronic device
- The electronic CRF system
- Study drug and randomization procedure
- Sampling, storing and sending procedure for blood analyses
- Procedure for study protocol violations and study drug non-compliance

**Informed consent**

Before enrolling the patient in the trial, i.e. before any trial-related examination is performed, Informed Consent must be obtained. Each potential participant will be informed, orally and in text, of the aim of the trial and what participation entails. They will be supplied with an Information Sheet summarizing the details of the trial. Ample time should be given to the patients to consider their participation. Agreement to participate in the trial is to be confirmed by the patient signing and dating the ICF. Documentation of this procedure is required. Documentation of the procedure is confirmed by the person who conducted the informed consent discussion signing and personally dating the dating the ICF.

**Breath alcohol concentration (BrAc)**

BrAc will be measured at visit 1, to ensure sobriety during the informed consent and when perfoming the cognitive test (CPTA) and at visit 8, where CPTA is once again performed. BrAc is measured in exhaled air with a hand-held device, an Alcometer, and will provide an accurate estimate of the blood alcohol level. The Alcometer must be calibrated and documented, and used according the manufacturer’s directions.

**Demography**

The demographic data that will be collected are: Gender and date of birth, civil status, number of children, and education. The age will be calculated in the eCRF.

**AUD diagnosis**

Documented diagnosis meeting ≥4 criteria according DSM-V by using structured interviewing.

**Medical history**

- History of previous psychiatric disorders, special attention to depression, previous suicidal attempts and other substance use disorders
- Previous and current medical conditions.
- History of alcohol dependence: familial cases of alcohol dependence, age of onset, mode(s) of alcoholisation, list of alcohol-use, CNS and somatic disorders, liver function tests, previous attempts of detoxification, alcohol consumption during the last month prior to inclusion. History of delirium tremens or abstinence-induced seizures
- Seizures, life time, alcohol-induced and non-alcohol-induced (see medical conderns section 1.3)
- Previous and current medication

**Physical examination**

A general physical examination including weight, examination of throat, armpits (lymph nodes), examination of mouth and throat, auscultation of heart and lungs, and palpitation of abdomen will be performed at the time points indicated above. Attention will be paid to abnormalities, which may have a bearing on the conduct of the trial (e.g., hypertension).

**Vital Signs**

The vital signs taken are blood pressure (systolic and diastolic) and heart rate and will be measured after 10 minutes of rest.The weight will be measured in kilograms with the patient lightly clothed but without shoes.

*Clinical Laboratory parameters*

**Phosphatidylethanol in blood (B-PEth)**

The direct alcohol marker PEth is found in red blood cells in humans in the presence of alcohol (Varga et al., 2000). The sensitivity of PEth is 94.5% and the specificity 100% (Hartmann et al., 2007) ,which is higher than for the commonly used alcohol markers CDT and GGT (Aradottir et al., 2006;Stewart et al., 2010;Hahn et al., 2012). The half-life of PEeth is approx. one week, and the detection window days to several weeks (Wurst et al., 2010;Gnann et al., 2012;Hill-Kapturczak et al., 2018). There is no gender difference (Stewart et al., 2009) and liver disease has no influence on PEth levels (Stewart et al., 2009;Stewart et al., 2014). The positive correlation between PEth and alcohol intake has been established (Gnann et al., 2012;Stewart et al., 2014;Kechagias et al., 2015). PEth as a primary variable is shown to be superior to alcohol markers CDT and GGT (Walther et al., 2015) as well as self-reported alcohol intake (de Bejczy et al., 2015).

**Carbohydrate-deficient transferrin (CDT)**

CDT is a sensitive and specific indirect alcohol marker for recent moderate to heavy drinking. The iron-transporting protein transferrin is synthesized and secreted by the liver (Hashimoto et al. 2013) and the amount of the deficient isoforms of transferrin increases in response to heavy drinking. The exact amounts of alcohol required to elevate CDT are not known, although an intake of 50-80 g per day during 2-3 weeks probably is sufficient, at least in alcohol dependent individuals (Stibler 1991; Mikkelsen et al. 1998; Winkler et al. 2013). With a half-life reported in-between 7-16 days, the most reliable time frame of CDT is between 7-10 days (Hashimoto et al. 2013). As the methodology to measure CDT has not been standardized, the exact sensitivity and specificity of CDT is still not fully established (Helander et al. 1997). The sensitivity seems to be dose-dependent, with discrepancies in reported sensitivity as wide as 26 to 83 % (Hashimoto et al. 2013).

**Gamma-glutamyl transferase (GGT)**

GGT is a membrane-bound glycoprotein enzyme which increases in serum with chronic alcohol intake. Although widely used, the specificity and sensitivity of GGT vary in different studies. As in the case of CDT, the sensitivity of GGT seems to be dose-related (Aradottir et al. 2006). A daily alcohol intake of 80-200 g for several weeks is needed to detect activity of GGT in blood (Winkler et al. 2013). GGT has a half-life of approx. 4 weeks (Hashimoto et al. 2013) and increased activity returns to normal in 2-5 weeks after discontinued alcohol intake (Niemela 2007; Winkler et al. 2013).

*Scales and questionairres*

**Adult ADHD self-report scale (ASRS)**

*Eligibility measurement.* The ASRS is a symptom checklist to help screen for ADHD in adult patients (Kessler et al., 2005). The questions in the ASRS v1.1 are consistent with DSM-IV criteria and address the manifestations of ADHD symptoms during the previous six months in adults. The checklist takes about five minutes to complete. For Swedish validated version of the ASRS scale, see Attachment 1.

**Alcohol Use Disorders Identification Test (AUDIT)**

*Outcome measurement*. AUDIT is a validated 10-item self-report scale developed by the World Health Organization to assess alcohol consumtion, drinking behaviors and alcohol-realated problems (Bohn et al., 1995;Babor TF, 2001). AUDIT was developed as a simple method of screening for excessive drinking and to assist in brief assessment. The AUDIT has been validated across genders and in a wide range of racial/ethnic groups. For Swedish validated version of the AUDIT scale, see Attachment 2.

**Drug Use Disorder Identification Test (DUDIT)**

*Eligibility and safety measurement.* DUDIT is a psychometric 11-item self-report questionnaire focusing on current drug-related problems (Berman et al., 2005). The DUDIT screens effectively for drug-related problems. The final 11 items in the DUDIT were chosen to give information on the level of drug intake and fulfilment of selected criteria for substance abuse/harmful use and dependence according to the ICD-10 and DSM-4 diagnostic systems. The DUDIT was developed and tested in Sweden, with the intention of expanding trials in Europe and elsewhere. For Swedish validated version of the DUDIT scale, see Attachments.

**Montgomery-Asberg Depression Scale (MADRS)**

*Eligibility and safety measurement.* MADRS is a validated 10-item self-rating scale for depressive symptoms and will be used as safety measure to monitor tentative changes in depressive status (Montgomery and Asberg, 1979). The included items are apparent sadness, reported sadness, inner tension, reduced sleep, reduced appetite, concentration difficulties, lassitude, inability to feel, pessimistic thoughts and suicidal thoughts. For Swedish validated version of the MADRS scale, see Attachments.

**Temporal experience of pleasure scale (TEPS)**

*Outcome measurement.* Anhedonia is a key feature of depression and negative symptoms of schizophrenia and is associated with social functioning in these patients. Anhedonia refers to the loss of or a reduction of the ability to experience pleasure. The temporal experience of pleasure scale (TEPS) is a 17-item scale with anticipatory and consummatory components of the experience of pleasure (Li et al., 2018) and is validated in a german population (Martinotti; et al.;Simon et al., 2017). TEPS will be used in the trial as a proxy to assess the hypodopaminergic (ventral striatum) hypothesies of AUD. For Swedish validated version of the TEPS scale, see Attachments.

**Timeline Followback (TLFB)**

*Outcome measurement.* The TLFB is a validated technique for assessing self-reported alcohol consumption. The drinking assessment method has been evaluated with clinical and non-clinical populations (Sobell and Sobell, 1992). People provide retrospective estimates of their daily drinking over a specified time period that can vary up to 12 months from the interview date. Several memory aids can be used to enhance recall e.g., calendar; key dates serve as anchors for reporting drinking; standard drink conversion. At screening, the subject will, together with the site staff, complete TLFB retrospectively for a period of 30 days before the visit. At every visit, the subject will complete the TLFB calendar covering the period between two following visits. The records will be checked and documented by the Investigator at each visit. The alcohol consumption will be quantified by collecting type of alcoholic beverage, volume percentage (% v/v) of alcohol and amount (e.g cl or ml) of beverage and converted into of pure alcohol in grams. For Swedish validated version of the TLFB calender, see Attachments.

**VAS Craving**

*Outcome measurement*. This is a single item instrument used to assess craving for alcohol and has previously been used in clinical trials. The alcohol craving is quantified via the Visual Analogue Scale, or VAS (Aitken, 1969). The VAS Craving Scale will not be administered via eCRF as the VAS scale is developed as a pencil-and-paper self-administration tool. A paper copy will be the source document. The Craving scale entails one question only. For Swedish version of the VAS Craving scale, see Attachments.

**Continuous Performance Test + Activity (CPTA)**

*Outcome measurement.* Opatus® CPTA is a neuropsychiatric tool that addresses the three entities inattention, impulsivity and activity (www.opatus.se). The test is presented on a mobile unit. Various symbols are flashed on to the screen and the task is to respond, by tapping the screen, to certain symbols (targets), and to not respond to other symbols (non-targets), as quick and accurate as possible. Although a simple task, its limited amusement factor makes it difficult for some to keep up the attention causing erratic responses to be more frequent. Hyperactivity is one of the core symptoms of ADHD. The CPTA utilizes built-in accelerometers and gyros in the smartphones to assess motor activity level. *Attention*: Opatus CPTA monitors how often presented targets are not responded on and how consistent correct responses are in time. *Impulsivity*: Opatus CPTA monitors how often responses are registered when no targets are present, how often responses are so quick (<150 ms) they are considered guesses rather than responses, or multiple responses to one symbol*. Activity*:  Opatus CPTA records how the mobile has been moved around in the 3D space during the test. The test is administrated via a mobile unit (iPod). Test time: 20 minutes. Opatus CPTA will be used in the trial as a proxy to assess the hypofrontality/hypodopaminergic (frontal cortex) hypothesis of AUD.

**Clincal Laboratory parameters**

*Eligibility, safety and outcome measurements.* Appointed by the coordinating center, a central laboratory will analyze B-PEth from all study sites. Safety parametes and secondary outcome measures will be analyzed at the Investigator’s site according to common practice. The Investigator at each study center will review and evaluate the results of the blood and urine safety analysis samples obtained at the screening visit to determine the patient‘s eligibility. The laboratory variables that will be determined are listed in Table 3. Before the patient is allowed to enter the study, clinical laboratory blood and urine tests will be performed and the absence of clinically significant abnormalities will be confirmed by the Investigator.The time of blood sampling will be recorded in the laboratory requisition form and transferred to the eCRF. A laboratory manual with detailed information regarding sample collection, handling, and labeling will be available at each stiudy site.

| **Alcohol markers** | **Hematology** | **Biochemistry** | **Urinalysis** | **Saliva analysis** |
| --- | --- | --- | --- | --- |
| Phosphatidylethanol  (At each visit) Carbohydrate deficient transferrin  (At each visit)  Gammaglutamyl-  Transferas  (At each visit)  *In exhaled air*  BrAC ( v1, v8) | Hemogloboin  (v1, v9)  Leukocyte plasma count (v1,v9)  Thrombocyte particle concentration  (v1, v9) | Aspartate Aminotransferase, Alanine Aminotransferase  (v1, v4, v9)  Glucose  (v1 and v9)  Na+/K+ (v1 and v9)  High sensitive C-reactive protein  (v1, v9)  Creatinine (v1, v4, v9)  Prothrombin Complex  (v1, v4, v9) | *Women* Human chorionic gonadotropin (v1, v2, v9)  *All*  Drug of abuse  (toxicology)  (v1,v2 v9) | Cotinine  (v2, v8) |
|  |  |  |  | **Pharmaco.**  **kinetic**  Drug (BUP, VAR) plasma concentrations (v4, v6) ** |

**Table 3.** List of Clinical laboratory parametes that will be analyzed in the trial

* Amphetamines/Methamphetmine, Barbiturates, Benzodiazepines, Cannabinoids, Cocaine, Ecstasy, Heroin, Opiates, Methadone, Tricyclic Antidepressants ** To be determined/analysed after study database closure to maintain double-blind condition

# Study Population

## Inclusion criteria

1) Signed informed consent

2) Blood alcohol level below <0.1‰ (0.1 g/L) at signing informed consent

3) 25-70 years of age at screening

4) Moderate and severe AUD according to DSM-V (meeting ≥4 out of 11 criteria)

5) B-PEth levels of ≥0.5 µmol/L at screening visit (visit 1) as defined by at least 2 HDD per week on a typical week.

6) Continuous high alcohol consumption over the last 3 months prior to screening

7) Available phone number for contact

8) Ability to speak and write in Swedish

## Exclusion criteria

Subjects must NOT enter the study if any of the following criteria are fullfilled:

1) Total abstinence between screening and randomization visit

2) Treatment of alcohol withdrawal within 30 days of study initiation

3) Pharmacological treatment within 3 months of study initiation and during the study period that may affect alcohol consumption, including but not exclusive to, varenicline, bupropion, disulfiram, acamprosate, naltrexone, nalmefene, baclofen, topiramate, ondansetron, mirtazapine, methylphenidate, dexamphetamine, atomoxetine, pregabalin, buprenorphine and methadone

4) Non-pharmacological treatment within 3 months of study initiation and during the study

period that may affect alcohol consumption

5) Current continuous use of antidepressants, opioid analgesics, benzodiazepines, zopiclone, zolpidem, hydroxyzine, alimemazine, propiomazine or other sedatives. (The sporadic use of these compounds is accepted.)

6) Any concurrent medication that may affect the results of the trial or is considered to

compromise the safety of the participants in the trial. (See SmPCs for possible interactions.)

7) Laboratory hepatic values of >3 times the upper limit of the normal range, creatinine clearence <30 ml/min, or other clinically significant abnormalities in the screening laboratory values

8) Blood pressure of 180/110 or higher at screening

9) Pregnancy, breast-feeding and for premenopausal women, not using one of the contraceptive methods; oral contraceptive, intrauterine contraceptive device (copper or hormonal) or subcutaneous inplant.

10) Diabetes mellitus type 1 and diabetes mellitus type 2 in need of insulin treatment

11) Any current psychiatric or somatic disorder or condition that may affect assessments or compromise participant’s safety during the trial

12) ASRS- v1.1, part A score ≥4 in the marked cut-off section

13) MADRS score ≥ 20

14) Current depression that is not mild (mild depression is accepted)

15) Suicidality

16) Current illicit drug use based on urine-toxicity test and DUDIT

17) History of Delirium Tremens or abstinence-induced seizures within 5 years of study initiation

18) Epilepsia or seizures other than alcohol-induced, lifetime

19) Severe sleep disturbances

20) Need of alcohol detoxification

21) Living conditions not appropriate to fulfill study requirements

22) Use of herbal drugs/tea and supplementations possibly affecting outcome or safety

23) Previous participation in this trial or in another trial within 3 months of enrollment into this trial

24) Additional factors that render the participant unable to complete the study, as judged by the investigator

## Subject enrolment and randomization

There will be a continuous enrolment of subjects from March 2019 until the PI stops the enrolment phase after decision. Subject eligibility (Visit 1) will be established before treatment randomization (Visit 2). Subjects will be randomized strictly sequentially, as subjects are eligible for randomization. If a subject discontinues from the study, the subject number will not be reused, and the subject will not be allowed to re-enter the study.

## Discontinuation and withdrawal of subjects

The participation in the clinical trial is voluntary and the subjects can decline further participation at any time without providing any motive for the discontinuation. They can also without any given reason at any time demand that their blood and urine samples are destroyed and that their randomization number is removed. If a subject discontinues or is withdrawn from the trial, EoT visit will be performed. In addition, any otcome measures at the ordinary visit that are not covered by the EoT visit will be collected (applicable for visit 4, 6 and 8). AEs will be followed by one (day +7), or if the AEs persists, by two phone calls (day +30).

Withdrawal of subject may take place due to:

- **Incorrect enrolment**

The exclusion criteria (Section 4.2.) apply during the entire study period. Based on the exclusion criteria, subjects will be excluded from the trial before the start of the trial and subjects may also be excluded from the trial and/or study drug during the whole study period

- **Protocol deviations**

The subjects may be withdrawn from the study at the discretion of the investigator if judged non-compliant with study procedures. Subjects will be excluded from the analysis set, if they are no longer judged to fulfill the eligility criteria; e g. if the subject has initiated a prohibited medication or other medication not listed or a non-pharmacological treatment considered to compromise the outcome measures in the study. A list of important protocol deviations will be finalized prior to study un-blinding. The number and percentages of patients with at least one important protocol deviation will be summarized by treatment arm and overall. In addition, the number and percentages of patients within each type of protocol deviation will be provided as well, and a listing of all patients with any important protocol violations/deviations will be provided for the ITT population.

- **Safety Concerns**

If the subject’s safety may be compromised, the subject may be withdrawn from the study and/or study drug at the discretion of the Investigator. Subjects must be excluded from treatment in the case of AE where continued treatment, as judged by the Investigator, may compromise the health of the trial subjects. The treatment code must not be broken except in medical emergencies when the appropriate management of the subject necessitates knowledge of the treatment randomization. In case of reporting of AE that may affect the benefit-risk balance, subjects may be unblinded for decision on an abortion of the trial. Subjects will be excluded from the analysis set if an AE considered to affect the assessments and/or the result of the trial is reported, even though subject safety is not compromised. In either case, serious adverse events (SAE) will be followed up.

- **Other reasons for discontinuation**

Subjects lost to follow-up.

## Premature termination of the study

The sponsor or competent authority (CA) may decide to stop the trial prematurely, if, for example the perception of the benefit/risk ratio becomes unfavorable for the continuation of the trial. If the trial is prematurely terminated or suspended, the investigator should promptly inform the subjects and ensure appropriate therapy and follow-up. Furthermore, the investigator should promptly inform the Ethics Committee and provide a detailed written explanation. The CA should be informed according to national regulations.

# STudy Treatments

## Identity of investigational medicinal products


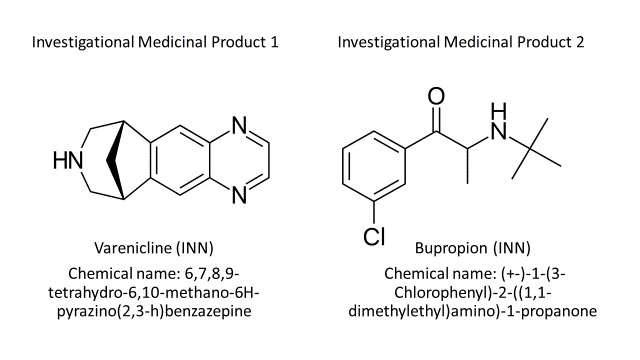


**Figure 2**. Molecular structure of varenicline and bupropion.

**IMP 1 Varenicline**

The chemical substance is varenicline tartrate. The salt is a mixture of possible conformations.

Molecular formula C_17_H_19_N_3_O_6_. Molecular weight 361.354 g/mol.

Brand names Champix, Chantix

ATC code N07BA03

VAR is a highly selective partial agonist of the nAChR of subtype conformation α_4_β_2_ and a full agonist on α_7_ nAChRs. [Nicotine](https://pubchem.ncbi.nlm.nih.gov/compound/Nicotine) stimulation of central α_4_β_2_ nAChRs located on dopaminergic cell bodies in the ventral tegmental area and on presynaptic dopaminergic terminals in the nAc causes the release of the neurotransmitter DA in nAc (see Section 1.1. for mechanistic rationale). As a nAChR partial agonist, VAR slightly elevates brain DA levels and attenuates the craving and withdrawal symptoms that occur with abstinence from [nicotine](https://pubchem.ncbi.nlm.nih.gov/compound/nicotine) but is not habit-forming itself. As a partial DA stimulator, VAR also prevents a full stimulation if nicotine is co-administered. VAR is the most effective monotherapy of smoking cessation according to meta-analyses (Mills et al., 2012) and the largest RCT for nicotine dependence to date (Anthenelli et al., 2016). For additional information regarding the investigational product, please see SmPC for VAR (Attachment 1).

Varenicline tartrate is manufactured by Pfizer Innovations, New York and registered as smoking cessation drug in Europe and the US with brand names Champix® and Chantix®, respectively. In Sweden, Champix® was registered by Pfizer 2006-09-26. VAR is prescription only medication.

**IMP 2 Bupropion**

The chemical substance is bupropion hydrocholoride in a sustained release formula

Molecular formula C_13_H_18_ClNO. Molecular weight 239.743 g/mol.

Brand name Zyban, Wellbutrin

ATC code N06AX12

BUP is classed as a noradrenaline-dopamine reuptake inhibitor (NDRI) as BUP elevates brain levels of NA and DA by blocking the action of the NA transporter and the DA transporter, respectively. BUP is a non-[nicotine](https://pubchem.ncbi.nlm.nih.gov/compound/nicotine) aid to smoking cessation and an antidepressant of the aminoketone class, thus chemically unrelated to tricyclic, tetracyclic, selective [serotonin](https://pubchem.ncbi.nlm.nih.gov/compound/serotonin) re-uptake inhibitor, or other known antidepressant agents. BUP does not inhibit monoamine oxidase. Pre-clinical studies suggest that bupropion may also act as a non-competitive nicotine antagonist. In smoking cessation, BUP is more effective that placebo, and similarly effective to naltrexone (Anthenelli et al., 2016).

Bupropion Sandoz (Sandoz AS) will be used in the manufacturing of IMP in the present trial. The rationale lies in a beneficial tablet size-best prize ratio. For additional information regarding the IMPs please see Appendix 2; SmPC BUP. BUP is registered in Sweden for smoking cessation (Zyban®) and for major depression (Voxra®) by GlaxoSmithKline and several forms of generic substitution medications that are regarded ‘essentially similar’ by the EU commission are available.

## Doses and treatment regimens

APL will manufacture, by using a procedure for encapsulation of tablets, package and label the study drugs according to Good Manufacturing Practice (GMP). The IMPs will be packed into unit-dose blister packs. The formulation of the IMPs and placebo will be indistinguishable with respect to appearance, shape, smell and taste. An appointed company will distribute the study drugs according to Good Distribution Practice (GMP) standard to the appointed Hospital Pharmacies/Study Centers.

*Investigational medicinal product (IMP) 1: Varenicline*

IMP Dosing 2 mg p.o. daily, ordinated as 1 capsule morning (1 mg) and 1 capsule in the evening (1 mg)

The pharmaceutical IMP formulation will be encapsulated tablets for oral use.

Each patient kit will contain VAR blister packed in secondary packaging and labeling.

VAR will be escalated from 0.5 to 2 mg daily during the first week as follows:

Day 1-3: 0.5 mg or corresponding placebo once daily

Day 4-7: 0.5 mg or corresponding placebo twice daily

Day 8-91: 1 mg or corresponding placebo twice daily

*Investigational medicinal product (IMP) 2: Bupropion SR*

IMP Dosing: 300 mg p.o daily ordinated as 1 capsule morning (150 mg or placebo) and 1 capsule in the evening (150 mg or placebo)

The pharmaceutical IMP formulation will be encapsulated tablets for oral use.

Each patient kit will contain VAR blister packed in secondary packaging and labeling.

BUP will be escalated from 150 to 300 mg daily during the first week as follows:

Day 1-7: 150 mg or corresponding placebo once daily

Day 8-91: 150 mg twice or corresponding placebo twice daily

No dose adjustments are allowed in the study.

*Double blind treatment phase (Week 0-Week 13)*

The IMPs will be distributed to the study subject at 7 occasions: Day 0, Day 7, Day 21, Day 35, Day 99, Day 63 and Day 77. The IMPs will be distributed at every visit to cover the next 14-days treatment period. If regarded necessary, IMPs for more than one treatment period may be distributed. The route of administration and the doses are chosen to evaluate the efficacy of the compounds on alcohol consumption in alcohol dependent individuals. Both compounds are previously registered as an oral medication for smoking cessation with the same dosing regimen and bupropion is also registered for depression.

*Randomization*

380 subjects will be randomized in the study in a 1:1:1:1 fashion, i.e. 95 subjects to each treatment arm

1. Varenicline + Bupropion SR
2. Varenicline + Placebo
3. Bupropion SR + Placebo
4. Placebo + Placebo

No treatment of AUD with the IMPs will be allowed beyond visit 9

## Labelling

The labeling text for IMP1 and IMP2 is described in Appendix 1. APL, the appointed manufacturer of the IMPs, will produce, pack and label VAR and placebo, and BUP and placebo, respectively, in two separate series. This is a combination trial with two study drugs and in order to maintain adequate double-blind condition, the name of the active ingredient will not be revealed in the labelling text. The treamtment kits containing VAR/Placebo and BUP/Placebo will be labelled Study drug 1/ Placebo and Study drug 2 Placebo, respectively. Note that the first study drug kits (for week 1) contain a separate labeling with instructions for dose escalation, as described in Appendix 1. The packages for the remaining treatment weeks contain study drug for 14-day use and 7 extra capsules to allow the accepted range between study visits (35 capsules in total) and will have identical labeling and dose instructions. Appendix 1 gives detailed information on the labeling text for the blister packs (inner packaging) and the secondary boxes (outer packaging) for IMP1/Placebo series and IMP2/Placebo series.

## Storage and handling

The IMPs will be stored in a lockable locker. The required storage conditions for IMP 1 and IMP 2 is room temperature, i.e. not above 25 C°, and to be kept in a dry place with no exposure to direct sun light. A thermometer will be placed in each locker to assure that appropriate temperature is maintained. There will be a temperature log at each site for documentation. The temperature log will be filled in every day during workdays.

## Drug accountability and treatment compliance

The study will ensure adequate drug accountability procedures for regulatory compliance. The compliance to study medication will be assessed by 1) pill count and 2) by determination of drug plasma concentrations at two time points during the trial (visits 4 and 6).

- *Pill Count*. Study personell will check the returned study drug kits at every visit (except for the randomization visit were there is no return) and count if the used medication corresponds to the required administration days. If not, the reason needs to be clarified, together with the study subject, and then documented by study personell.
- *Pharmacokinetic monitoring*. Blood will be drawn at visit 4 and 6 for determination of plasma drug concentrations. In order to obtain meaningful results, the study subjects will be instructed to not take the morning dose before coming to visits 4 and 6.

## Destruction

Before the IMPs can be returned for destruction, the investigator must keep documentation that include dates, quantities, patient numbers, batch number, expiry date and the ability to identify to whom the study drug was given. Residual capsules will be returned to the designated pharmacy for destruction. Destruction will be documented according to internal procedures.

## Identity of non-investigational medicinal products

There is no standard background therapy in the trial. However, the study subject may be allowed to use medication that does not affect alcohol consumption or compromise the safety of the study subject, as judged by the investigator. See Section 5.9 for allowed medication during the trial.

## Blinding, randomization and breaking codes

All data will be treated in accordance with the European Union's General Data Protection Regulation (GDPR) Act of Maj 2018. The identity of the study subjects will be coded and data will be analyzed coded and based on group level.

**Blinding**

APL, the appointed manufacturer of the IMPs, will produce, pack and label VAR and placebo and BUP and placebo, respectively, in two separate IMP kits. APL will use a blinding procedure accordance wirh internal standard operating procedure. Two associated treatment kits, one for IMP 1 and one for IMP 2, will have an unique number, a Randomization Number, generated randomly. For each randomization number, a sealed emergency code envelope will follow the shipment. The coding will be revealed only after “Last patient out” and after a blind analysis.

**Randomization**

Following the screening visit, the subjects who meet the eligibility criterias will be randomly assigned to 1 of 4 treatment groups in a ratio of 1:1:1:1 to each of the four groups, in a blind manner. Each randomized subject will be allocated chronologically according to the Randomization lists, to a randomization number. The randomization number defines the IMP1 and IMP 2 and is therefore equal for series 1 and series 2. Subject data will be filed with its Randomization Number in the eCRF. Only by the Investigator delegated staff will have access to the eCRF.

**Breaking the code**

The Investigator has the right to unblind the given IMP in case of emergency and/or patients’s interest and if knowing the identification of the treatment arm would lead to the Investigator treating the patient/subject differently. In addition, within drug safety, the code may be broken on demand of regulatory reporting or on request of the person responsible for pharmacovigilance. One setup of emergency code envelopes will exist and will be kept by the Sponsor at the Coordinating center in a locked cabinet. The code will be broken only in case of Serious Adverse Events of or other serious incidents. Only the Investigator or the Sponsor is permitted to demand the unblinding.

**Procedure för Code breaking**

The Investigators at respective study center will call the 24 hours/day emergency telephone number to the Sponsor/Coordinating Investigator. Then, etiher the Coordinating Investigator or Sponsor’s delegate will open the envelope to reveal the treatment and directly communicate this by telephone to the Investigator.

For every patient whose blind has been broken the following information must be documented in the eCRF: The reason for, the date, and the time of unblinding and the person(s) informed of the treatment allocation must be identified. In order to maintain the double-blind nature of the trial, the allocation of IMP(s) for the patient must not be communicated further unless required for the surveillance of the patient or if necessary for urgent risk to benefit re-evaluation and/or measures for urgent risk minimization.

## Concomitant and prohibited medication

**Prohibited concomitant medication**

1. Pharmacological treatment within 3 months of study initiation and during the study period that may affect alcohol consumption, including but not exclusive to, varenicline, bupropion, acamprosate, naltrexone, nalmefene, disulfiram, ondansetron, topiramate, mirtazapine, methylphenidate, dexamphetamine, atomoxetine, pregabalin, buprenorfin or methadone (see Exclusion criteria)
2. Group-based (N06A*) Antidepressants
3. Group-based (N02A*) Opioids
4. Group-based (N05CD*). Sedatives including benzodiazepines. Note that the sporadic use of these compounds is accepted.
5. Any concurrent medication that may affect the results of the trial or is judged by the responsible medical doctor to compromise the safety of the participant in the trial. This section cover attention to all CNS active drugs, that is, section N1-N7* in the ATC register in FASS (www.fass.se)

*Classified according to the ATC-system (Anatomical, Therapeutic, Chemical Classification)

The Sponsor will provide a list over prohibited medication. See separate document.”

**Allowed concomitant medication**

Concomitant medication will be allowed except such that might interfere, in the best opinion of the Investigator, with the CNS function or assessment of study drug safety and/or efficacy. The allowed medications must be considered necessary for the patient’s safety and well-being and must be regarded by the Investigator to be harmless in combination with VAR and BUP. Concomitant medication will be recorded in the Case Report Form at every study visit.

# Study Measurements and Variables

## Safety variable(s)

The safety variables are summarized in Table 4. A laboratory manual with detailed information regarding sample collection, handling, and labeling of the safety varialbes will be available at each stiudy site.

| SAFETY VARIABLES |
| --- |
| AE/SAE-Adverse Events, Serious Adverse Events |
| AST- Aspartate Aminotransferase |
| ALT -Alanine Aminotransferase |
| Creatinine |
| Glucose |
| PC -Prothrombin complex |
| Na+/K+ |
| Hb -Hemoglobin |
| LPK - Leukocyte plasma count |
| TPK - Thrombocyte particle concentration |
| hsCRP-High sensitive C-reactive protein |
| Urine toxicology |
| Drug accountability by pill count |
| Drug plasma concentrations of VAR and BUP |
| MADRS self report scale for depression |
| DUDIT self report scale for drug abuse |

**Table 4**. Safety variables in the COMB-BO8 study

## Biological sampling procedures

### Handling, storage and destruction of biological samples

The investigational staff at every study site will receive thorough oral and written information regarding the handling of biological samples before its onset. There will be a check-list available regarding laboratory measurements during each visit.

**Blood samples**

Venous blood will be collected at every visit. i.e. at 9 occasions according to Table 5 below. At every visit, blood will be collected and analyzed for alcohol-related outcome measurements. The blood samples for safety outcome measures are taken at Vists 1, 4 and 9. Blood will be drawn at Visits 4 and 6 for determination of BUP and VAR through concentrations in plasma.

The primary alcohol specific outcome measurement B-PEth will be analysed at the coordinating site, by an appointed clinical chemistry laboratory that will serve as the central lab. Blood samples will be transported to the central lab at Center 1 by appointed biological material transfer agency. All other blood sampels will be analyzed att the local clinic according to internal procedure and destroyed after analysis. To maintain blinding throughout the study analysis of BUP and VAR concentrations will be performed after completion and deblinding of the study.

**Urine dip sticks**

Urine samples will be collected and analyzed to screen for pregnancy at Visits 1, 2 and 9 according to Table 3. Urine samples will be collected and analyzed to screen for illicit drugs at Visits 1, 2 and 9. Urine dip sticks (U-hCG and toxicology) will be analyzed at the local study site and destroyed after completion of data managment in the eCRF/ paper version of CRF.

**Saliva stick**

Saliva will be collected at visits 2 and 8 according to Table 3. The saliva stick wil be analyzed at the local study site and destroyed after completion of data managment in the eCRF/ paper version of CRF.

### Total volume of blood per patient

| **Visit** | **Blood Volume** | **Purpose** |
| --- | --- | --- |
| 1 | 45 ml | 15 ml for B-PEth, CDT and GGT analyses  20 ml for safety biochemical assessments (glucose, Hb, LPK, TPK, hsCRP)  5 ml blood are drawn for determination of PC  5 ml blood are drawn for AST, ALT Na+/K+, Creatinine |
| 2 | 15 ml | 15 ml for B-PEth, CDT and GGT analyses |
| 3 | 15 ml | 15 ml for B-PEth, CDT and GGT analyses |
| 4 | 35 ml | 15 ml for B-PEth, CDT and GGT analyses  5 ml blood are drawn for determination of PC  5 ml blood are drawn for AST, ALT, Creatinine  10 ml blood are drawn for plasma drug concentrations |
| 5 | 15 ml | 15 ml for B-PEth, CDT and GGT analyses |
| 6 | 25 ml | 15 ml for B-PEth, CDT and GGT analyses  10 ml blood are drawn for plasma drug concentrations |
| 7 | 15 ml | 15 ml for B-PEth, CDT and GGT analyses |
| 8 | 15 ml | 15 ml for B-PEth, CDT and GGT analyses |
| 9 | 45 ml | 15 ml for B-PEth, CDT and GGT analyses  5 ml blood are drawn for determination of PC  5 ml blood are drawn for AST, ALT, Creatinine  20 ml for other safety biochemical assessments (glucose, Na+/K+, Hb, LPK, TPK, hsCRP) |
| **Total** | **225 ml** | **Volume of blood drawn from each subject during the study** |

**Table 5**. Amount of blood drawn during the trial. Visit 1: 45 ml, Visit 2: 15 ml, Visit 3: 15 ml, Visit 4: 45 ml, Visit 5: 15 ml, Visit 6: 35 ml, Visit 7:15 ml, Visit 8:15 ml, Visit 9: 45 ml. The total volume of blood drawn from each subject during the study is 225 ml.

If required due to safety concerns or technical errors in analysis, additional blood up to 20 ml may be drawn. The maximum blood drawn per subject will then be 225 + 20 ml = 245 ml.

### Biobank

All biological samples collected in the study, which includes blood, plasma, serum, saliva and urine are classified as biobank material and will be stored in a regulatory qualified biobank and handled according to the multicenter principle* and the biobank act, Biobankslagen SFS 2002:297. The appointed biobank will be Biobank Väst. All biological samples collected in the study will be destroyed after analysis. The study participant can at any time demand the biological sample to be removed and destroyed. For detailed description of handling of biological data and instruction for demanding destruction, see ICF, Appendix 2.

*The multicenter principle refers to research that includes tests from several healthcare principals. In these studies, samples from several primary sample collections are handed out to a secondary sample collection in a biobank at the research head or with which the research chief (=Sponsor/Coordinating Site) has an agreement. ([www.biobanksverige.org](http://www.biobanksverige.org)). The approach means that the county councils that so desire establish a so-called e-biobank, i.e. an electronic biobank, in which sample is only recorded as a primary sample collection to ensure traceability under the biobanks.

# Safety

## Reference Safety Information

This section gives a reference safety information for the conductance of the trial and for the use of VAR in the dose of 1 mg x 2 daily and BUP in the dose of 150 mg x 2 daily, in combination and alone.

A comment regarding reported side effects of VAR and BUP in studies in nicotine dependence is that smoking cessation with or without treatment is associated with nicotine withdrawal symptoms and thus possible exacerbation of underlying psychiatric illness. Not all patients in these reports had known pre-existing psychiatric illness and not all had discontinued smoking. The exact role of VAR and BUP in these reported AEs is not known. Note that depressed mood, which in rare cases may include suicidal ideation or suicidal attempt, can be a symptom of discontinuation of nicotine use.

**IMP 1: Varenicline Reference Safety Information**

**Varenicline in AUD population**

VAR in the dose of 1 mg + 1 mg daily for 12 weeks has previously been assessed in subjects with AUD in clinical trials and reported to be safe and well tolerated (see sections 1.1 and 1.2). The adverse events (AEs) were generally of mild character and in concordance with the expected side effects (from the indication nicotine use disorder). With a prevalence of 30%, the most frequent reported AE was **nausea**, followed by **abnormal dreams, constipation, and dysgeusia**. The nausea was generally mild or moderate in intensity and seldom resulted in discontinuation. Nausea was most often experienced during the first week of treatment and did not constitute a tolerability concern (Litten et al., 2013;de Bejczy et al., 2015).

*Possible action to take to alleviate nausea side effects.* If a nausea judged to be possibly related to IMP and judged to be clinical significant by medical doctor, meclozine oral tablets of 25 mg (Postafen®) may be prescribed during the first 14 days of the treatment period to alleviate the initial side effect.

**Medical risks associated with varenicline**

*Depressive symptoms.* There have been previous reports of psychiatric AEs, including depressed mood, agitation, changes in behaviour and suicidal ideation in patients attempting to quit smoking while taking VAR. However, VAR is today judged not to influence suicidal ideation (Hong et al., 2015). In an observational prospective cohort of 3415 patients that collected real-life post marketing data on psychiatrivc adverse events, 3% of the study participants experienced symptoms of depression (Harrison-Woolrych and Ashton, 2011). Suicide, suicidal ideation and psychotic reactions were also identified, but these were less frequently reported. In a real-life study among 56 851 new VAR users, VAR appeared to be associated with an increased risk of cardiovascular but not neuropsychiatric events (Gershon et al., 2018) .

*Action taken to minimize risk of worsening tentative depressive symptoms by VAR:* Study subjects will be screened for depressive symptoms when entering the trial. The study does not inclue depresssive subjects, other than those with mild depression, which often is a part of AUD. Subjects will be screened for current depression and any history of depression during diagnostic interviewing, and any current depression that is not well-treated or mild and MADRS score > 20 are criteria for exclusion. Depressive symptoms will monitored by MADRS and collection of AE/SAE before, during (Visit 4) and after the treamtent period at (End-of-Treamtent, visit 9). If any indication of a worsening of such symptoms, the trial subjects will be examined by the Investigator who will decide whether drug treatment should be terminated and whether the subject will be refeered to receive adequate treatment.

**Expected Side effects**

The most commonly reported AE is nausea (28.6%) in patients treated with the recommended dose of 1 mg VAR twice daily following an initial titration period. In the majority of cases nausea occurred early in the treatment period, was mild to moderate in severity and seldom resulted in discontinuation.

The expected side effects of VAR are summarized in Table 6. All adverse reactions, which have occurred at an incidence greater than placebo are listed by system organ class and frequency (very common (≥ 1/10), common (≥ 1/100 to < 1/10), uncommon (≥ 1/1,000 to < 1/100) and rare (≥ 1/10,000 to < 1/1,000)). Within each frequency grouping, undesirable effects are presented in order of decreasing seriousness.

| **System Organ Class** | **Adverse Drug Reactions** |
| --- | --- |
| **Infections and infestations** | |
| **Very common** | **Nasopharyngitis** |
| Common | Bronchitis, sinusitis |
| Uncommon | Fungal infection, viral infection |
| **Blood and lymphatic system disorders** | |
| Rare | Platelet count decreased |
| **Metabolism and nutrition disorders** | |
| Common | Weight increased, decreased appetite, increased appetite |
| Uncommon | Hyperglycaemia |
| Rare | Diabetes mellitus, polydipsia |
| **Psychiatric disorders** | |
| **Very common** | **Abnormal dreams, insomnia** |
| Uncommon | Suicidal ideation, aggression, panic reaction, thinking abnormal, restlessness, mood swings, depression*, anxiety*, hallucinations*, libido increased, libido decreased |
|  |  |
|  |  |
| Rare | Psychosis, somnambulism, abnormal behaviour, dysphoria, bradyphrenia |
| **Nervous system disorders** | |
| **Very common** | **Headache** |
| Common | Somnolence, dizziness, dysgeusia |
| Uncommon | Seizure, tremor, lethargy, hypoaesthesia |
| Rare | Cerebrovascular accident, hypertonia, dysarthria, coordination abnormal, hypogeusia, circadian rhythm sleep disorder |
| **Eye disorders** | |
| Uncommon | Conjunctivitis, eye pain |
| Rare | Scotoma, scleral discolouration, mydriasis, photophobia, myopia, lacrimation increased |
| **Ear and labyrinth disorders** | |
| Uncommon | Tinnitus |
| **Cardiac disorders** | |
| Uncommon | Myocardial infarction, angina pectoris, tachycardia, palpitations, heart rate increased |
| Rare | Atrial fibrillation, electrocardiogram ST segment depression, electrocardiogram T wave amplitude decreased |
|  |  |
| **Vascular disorders** | |
| Uncommon | Blood pressure increased, hot flush |
| **Respiratory, thoracic and mediastinal disorders** | |
| Common | Dyspnoea, cough |
| Uncommon | Upper respiratory tract inflammation, respiratory tract congestion, dysphonia, rhinitis allergic, throat irritation, sinus congestion, upper- airway cough syndrome, rhinorrhea |
| Rare | Laryngeal pain, snoring |
| **Gastrointestinal disorders** | |
| **Very common** | **Nausea** |
| Common | Gastrooesophageal reflux disease, vomiting, constipation, diarrhoea, abdominal distension, abdominal pain, toothache, dyspepsia, flatulence, dry mouth |
| Uncommon | Haematochezia, gastritis, change of bowel habit, eructation, aphthous stomatitis, gingival pain |
| Rare | Haematemesis, abnormal faeces, tongue coated |
| **Skin and subcutaneous tissue disorders** | |
| Common | Rash, pruritus |
| Uncommon | Erythema, acne, hyperhidrosis, night sweats |
| Rare | Severe cutaneous reactions, including Stevens Johnson Syndrome and Erythema Multiforme, angioedema |
| **Musculoskeletal and connective tissue disorders** | |
| Common | Arthralgia, myalgia, back pain |
| Uncommon | Muscle spasms, musculoskeletal chest pain |
| Rare | Joint stiffness, costochondritis |
| **Renal and urinary disorders** | |
| Uncommon | Pollakiuria, nocturia |
| Rare | Glycosuria, polyuria |
| **Reproductive system and breast disorders** | |
| Uncommon | Menorrhagia |
| Rare | Vaginal discharge, sexual dysfunction |
| **General disorders and administration site conditions** | |
| Common | Chest pain, fatigue |
| Uncommon | Chest discomfort, influenza like illness, pyrexia, asthenia, malaise |
| Rare | Feeling cold, cyst |
| **Investigations** | |
| Common | Liver function test abnormal |
| Rare | Semen analysis abnormal, C-reactive protein increased, blood calcium decreased |
| * Frequencies are estimated from a post-marketing, observational cohort study | |

**Table 6**. Adverse Drug Reactions of Varenicline

**Reported adrevers events of varenicline in subjects with AUD**

The most prevalent AEs in the varenicline group were nausea (48.1% vs 12.0%), headache (22.1% vs.15.7%) and fatigue (22.1 vs. 10.8%) (de Bejczy et al., 2015). Nausea and abnormal dreams (16.9% vs. 3.6%) were significantly greater in the VAR group compared to placebo (de Bejczy et al., 2015). The AEs that differed significantly between VAR and placebo were nausea (37.1 vs 17.8%), abnormal dreams (27.8% vs. 11.9%) and constipation (9.3% vs 2.0%) (Litten et al., 2013). Compared with placebo, varenicline did not increase suicidal ideation, mood changes or agitation in either study. In sum, the AEs among subjects with AUD are consistent with the side-effects listed in the table above (and ad in SmPC).

**Varenicline overdose**

No specific antidote for VAR is known. In the event of overdose, hospitalization is advised and management should be as clinically indicated. Hemodialysis may be considered in cases of overdose. No clinically meaningful changes in laboratory test results were observed in the previous clinical trials, nor were there noteworthy changes in the QT/QTc interval or any other ECG parameter in either preclinical or clinical studies.

**Varenicline contraindications**

Varenicline is contraindicated in pregnant or nursing women and in patients less than 18 years of age. These are exlusion criteria.

**IMP 2: Bupropion Safety Reference Information**

**Bupropion in AUD population**

No controlled clinical trials have assessed BUP in alcohol dependent subjects. However, in the clinical trials assessing combination therapy with VAR and BUP for smoking cessation, alchohol consumption has not been excluded.

**Medical risks associated with bupropion**

The medical risks associated with BUP in this trial are regarded to be the same as those listed in the registration documents for BUP used in smoking cessation (SmPC for BUP, Attachment 2). In 2003, the five years post-marketing report concluded that BUP is efficacious, safe and well tolerated for treatment of nicotine dependence (Ferry and Johnston, 2003). Further, the report identified two medical risks associated with BUP: major motor seizure (incidence is appr. 0.10%) and hypersensitivity reaction (incidence is appr. 0.12%). The combined risk of experiencing one of the two potentially serious adverse events during treatment is 0.22% (Ferry and Johnston, 2003). In nicotine use disorder, the most frequent reported AE/side effect in clinical trials and clinical practice is **insomnia**. Note that this can also be a symptom of nicotine withdrawal as such. Other common and expected AEs are **decreased appetite, gastrointestinal disturbance, dry mouth, headache and dizziness**. The same AEs are expected in the present trial population. See Section 7, Safety for the full list on expected side effects.

*Seizures.* BUP has been shown to increase the risk of seizures. The seizure threshold decreases on abrupt cessation of excessive drinking, thus seizures may occur during withdrawal (6-48 hours after the cessation of drinking). The relationship of seizures to alcohol use is likely to be dose dependent and causal (Hillbom et al., 2003). Available clinical data suggest that excessive alcohol drinking is contraindicated for BUP (SmPC BUP, Attachment 2). In this trial, there will be no abrupt cessation of alcohol intake and subjects with a previous history of seizures are exluded. It is therefore the Sponsor’s judgment that there will be an equal or even lower risk of seizures in this trial compared to the approved indication. If however, a seizure occurs, it will be considered and reported as SAE and the subject will immediately be withdrawn from the study and referred to appropriate medical care. The Investigator will follow up the subject until treatment termination and notify the outcome to the Sponsor. *Action taken to mitigate or minimize the risks of eliciting seizures during BUP administration*: Subjects will be screened for lifetime incidence of seizures, which is a criterium for exclusion. The absence of a history of seizures will be confirmed through collection of medical history during prescreening and during screening. To mitigate or minimize alcohol withdrawal that may predispose for seizures, subjects will be screened for a periodic pattern of alcohol consumption that is a criterium for exclusion. If a seizure occurs during the trial the study subject will immediately be excluded from the trial (see exclusion criteria).

*Hypertension.* A rare side effect of BUP is acute hypertension, observed in patients both with and without previous hypertonia. *Action taken to mitigate or minimize the risks of worsening of cardiovascular symptoms during BUP adminstration:* Blood pressure will be monitored before treatment with study drug and at every study vist during the trial (9 visits in total). If a sudden increase in blood pressure is observed and judged by the Invesigator as clinically significant, the study subject will immediately be referred to its primary care physician or adequate medical care and will be instructed not to take the next dose of study drug. Adequate instructions regarding actions to be taken when a hypertension, defined as 160/95 or higher, is observed will be provided by the Sponor and given to each Investigator.The Investigator will make a judgement whether it is a SAE and whether the study drug should be discontinued.

*Insomnia*. A common side effect of BUP is insomnia. Note that smoking cessation with or without treatment is associated with nicotine withdrawal symptoms and the exacerbation of underlying psychiatric illness. *Action taken to mitigate or minimize the risks of sleep disturbance during BUP administration*: subjects experiencing insomnia will be advised to take the second dose of IMP earlier (e.g. 15 pm) instead of at bedtime. If the sleep disturbance is still judged clinically significant by medical doctor, zolpidem oral tablets of 5 mg may be prescribed during the first 14 days of the treatment period.

*Depressive symptoms*. A common reported side effect of BUP is depressive symptoms.

*Action taken to minimize the risk of worsening of depressive symptoms by BUP:* The study does not include depresssive subjects, other than subjects afflicted with mild depression, which often is a part of AUD. Subjects will be screened for current depression and any history of depression during diagnostic interviewing, and any current depression that is not well-treated or mild and with a MADRS score > 20 is criterium for exclusion. Depressive symptoms will be monitored by MADRS and collection of AE/SAE before, during and after the treatment period. If any indication of a worsening of such symptom, the trial subjects will be examined by the Investigaotr, who will decide whether drug treatment should be terminated and whether the subject will be refered to receive adequate treatment.

The expected side effects of BUP are summarized in table 7. All adverse reactions, which have occurred at an incidence greater than placebo are listed by system organ class and frequency (very common (≥ 1/10), common (≥ 1/100 to < 1/10), uncommon (≥ 1/1,000 to < 1/100) and rare (≥ 1/10,000 to < 1/1,000)). Within each frequency grouping, undesirable effects are presented in order of decreasing seriousness.

| Blood and lymphatic system disorders | Not known | Anaemia, leucopenia and thrombocytopenia |
| --- | --- | --- |
| Immune system disorders* | Common | Hypersensitivity reactions such as urticaria |
|  | Rare | More severe hypersensitivity reactions including angioedema, dyspnoea/bronchospasm and anaphylactic shock. Arthralgia, myalgia and fever have also been reported in association with rash and other symptoms suggestive of delayed hypersensitivity. These symptoms may resemble serum sickness |
| Metabolism and nutrition disorders | Uncommon | Anorexia |
|  | Rare | Blood glucose disturbances |
|  | Not known | Hyponatraemia |
| Psychiatric disorders | **Very common** | Insomnia |
|  | Common | Agitation, anxiety |
|  | Uncommon | Depression, confusion |
|  | Rare | Irritability, hostility, hallucinations, depersonalisation, abnormal dreams including nightmares |
|  | Very rare | Delusions, paranoid ideation, restlessness, aggression |
|  | Not known | Suicidal ideation and suicidal behaviour***, psychosis |
| Nervous system disorders | Common | Tremor, concentration disturbance, headache, dizziness, taste disorders |
|  | Rare | Seizures (see below)**, dystonia, ataxia, Parkinsonism, incoordination, memory impairment, paraesthesia, syncope |
| Eye disorders | Uncommon | Visual disturbance |
| Ear and labyrinth disorders | Uncommon | Tinnitus |
| Cardiac disorders | Uncommon | Tachycardia |
|  | Rare | Palpitations |
| Vascular disorders | Uncommon | Increased blood pressure (sometimes severe), flushing |
|  | Rare | Vasodilation, postural hypotension |
| Gastrointestinal disorders | Common | Dry mouth, gastrointestinal disturbance including nausea and vomiting, abdominal pain, constipation |
| Hepatobiliary disorders | Rare | Elevated liver enzymes, jaundice, hepatitis |
| Skin and subcutaneous tissue disorders* | Common | Rash, pruritus, sweating. |
|  | Rare | Erythema multiforme and Stevens Johnson syndrome have also been reported. Exacerbation of psoriasis |
| Musculoskeletal and connective tissue disorders | Rare | Twitching |
| Renal and urinary disorders | Rare | Urinary frequency and/or retention |
|  | Very rare | Urinary incontinence |
| General disorders and administration site conditions | Common | Fever |
|  | Uncommon | Chest pain, asthenia |

Table 7. Adverse Drug Reactions of Bupropion

Two studies with BUP SR 150 mg tablets in healthy volunteers suggest that exposure to BUP may be increased when BUP tablets are taken with food. When taken following a high fat breakfast, peak plasma concentration of bupropion (C_max_) increased by 11% and 35% in the two studies, while the overall exposure to bupropion (AUC) increased by 16% and 19%.

As with other CNS acting drugs BUP may affect ability to perform tasks that require judgement or motor and cognitive skills. BUP has also been reported to cause dizziness and lightheadedness. Study subjects should therefore exercise caution before driving or use of machinery until they are reasonably certain BUP does not adversely affect their performance. Study subjects will be given appropriate information about this tentative interaction at the randomization visit.

BUP should only be administered orally. Inhalation of crossed tablets or injection of diluted BUP has been reported and can lead to a fast absorption and a potential overdose. Seizures and/or death have been reported when BUP has been administerated intra-nasally or via parenteral injection. Such misuse of BUP will lead to immediate exlusion from the study and will be reported as a SAE (see section 7.2.3).

**Bupropion contraindications**

The eligibility criteria are defined so that all BUP contraindications, except for AUD, described in the SmPC for BUP will be avoided among the study subjects.

**Interference with urine testing**

Due the chemical similarity to amphetamine there is a risk that BUP will give false positive amphetamine urine drug screens (Casey et al., 2011). Urine toxicology dip stick is given at Screening Visit, randomization visit (Vist 1) and at End-of-Trial visit (Visit 9). A positive dip stick for amphetamine at visit 9 may need to be confirmed with a specific immunochemical analysis method.

**Bupropion overdose**

Acute intake of doses > 10 times the maximum therapeutic dose has been reported. In addition to those events reported as Undesirable Effects, overdose has resulted in symptoms including drowsiness, loss of consciousness and/or ECG changes such as conduction disturbances (including QRS prolongation), arrhythmias and tachycardia. QTc prolongation has also been reported but was generally seen in conjunction with QRS prolongation and increased heart rate. Most patients recovered without sequelae, yet there are some rare reports of deaths associated with BUP in patients ingesting large overdoses of the drug. The use of activated charcoal is recommended. No specific antidote for BUP is known. In the event of overdose, hospitalization is advised and management should be as clinically indicated.

**CYP2D6 inhibition by BUP and hydroxy-BUP**

Although not metabolised by the CYP2D6 isoenzyme itself, BUP and its main metabolite, hydroxybupropion, inhibit the CYP2D6 pathway and may thus increase the effect of certain drugs. *Action taken to prevent drug drug interactions*: To mitigate pharmacokinetic interactions, concomitant medication with drugs metabolized via CYP2D6 will be avoided as medical conditions requiring these drugs are exlusion criterias. (See prohibited medication, certain antidepressants and type 1 antiarrhythmics. For full list of interactions, see SmPc, Attachments 2.)

**Pharmacokinetic interactions between varenicline and bupropion**

VAR and BUP have no known clinically significant drug drug interactions. VAR does not change the pharmacokinetics of BUP at steady state and vice versa, there are no reports that BUP alters the excretion of VAR (92-93% excreted by the kidneys). BUP and its main active metabolite, hydroxybupropion, inhibit the CYP2D6 enzyme. *Action taken to minimize drug drug interactions*: According to clinical data, there are no pharmacokinetic interactions between BUP and alcohol. The SmPC for BUP states that there are a few reports on neuropsychiatric side effects and decreased tolerance to alcohol with BUP.

**Elderly population**

This trial includes subjects up to 70 years, whereof those between 65-70 years are classified as elderly (WHO definition is people aged 65 and over). This age group has a higher risk for reduced liver and kidney performance, and consequently a lower dose may be appropriate in some subject, as outlined in the SPC. It is the Sponsor’s judgment that dose reduction is not needed in this trial, despite including subjects up to age 70. For the purpose of safety, liver (by AST, ALT and PC) and kidney (by creatinine) status will be monitored before, during and after the treatment period.

**Expected side effects and medical risks of varenicline and bupropion used in combination**

In nicotine dependence, the safety profile of VAR and BUP in combination has been described in six studies (seven publications). In these studies, VAR was investigated in the dose of 1 mg twice daily and BUP in the dose of 150 mg twice daily (Except for study 4, where BUP was dosed as 150 mg once daily). Both drugs were titrated during the first week according to recommendations.

**Reference study 1**

VAR and BUP sustained-release combination therapy for smoking cessation (Ebbert et al., 2009). This was an open-label, one-arm, phase II pilot study with 38 enrolled subjects lasting 12 weeks with 6 months follow up. The study evaluated the combination as safe. The most common reported AE of combination therapy were sleep disturbance (26%), nausea (24%) and insomnia (16%). The study excluded active AUD but not alcohol consumption as such. Efficay outcome variable: prolonged abstinence at 12 weeks and at follow-up 6 months. Results showed 63% prolonged abstinence at 12 weeks and 53% at 6 months in the combination treatment group.

**Reference study 2**

Combination treatment with VAR and BUP in an adaptive smoking cessation paradigm (Rose and Behm, 2014). RCT, N=349. This was a 12-week randomized, adaptive treatment, double-blind phase III clinical trail. The study evaluated the combination as safe. The most common reported AEs of combo therapy were headache (9.3%), diaphoresis (8.8%), nasal/sinus irritation (5.9%), changes in taste perception (17.2%), dry mouth (10.8%), thirst (15.7%), cough (8.8%) irritability (11.3%), vivid dreams (18.1%), insomnia (13.7%) and anxiety (8.8%). The study excluded AUD but not alcohol consumption as such. Efficay: Outcome variable continuous abstinence at week 8-11. Combination therapy increased abstinence relative to varenicline monotherapy (39% vs 26%), OR 1.89. Male smokers had a greater effect than females; OR 4.26 vs 0.94. Highly dependent smokers had a greater effect; OR 3.51 vs 0.71.

**Reference study 3**

Combination VAR and BUP SR for tobacco-dependence treatment in cigarette smokers: a randomized trial (Ebbert et al., 2014). This was a 12-week randomized, blinded, placebo-controlled phase III clinical trial. RCT, N=506. The most common reported AEs of combination therapy were sleep disturbances (40.2%), nausea (22.1%), constipation (10.4%), anxiety (7.2%) and depressive symptoms (3.6%). The study excluded AUD but not alcohol consumption as such. Efficay: N=506, RCT, outcome variable prolonged abstinence at 12 weeks and 26 weeks. At 12 weeks Odds Ratio for varenicline monotherapy was 1.36 and for combination therapy combination 1.49. At 26 weeks monotherapy varenicline OR 1.32 vs OR 1.52 for the combination therapy. At 52 weeks OR 1.40 for varenicline monotherapy and 1.39 for the combination therapy.

**Reference study 4**

Effectiveness of co-administration of VAR, BUP, and serotonin reuptake inhibitors (SRI) in a smoking cessation program in the real-life setting (Issa et al., 2013). N=427. This was a retrospective outcome research study of patients that receiced VAR, VAR+BUP, VAR+SRI or VAR+BUP+SRI and were followed during 1 year. AEs were not collected in the study but nausea and abnormal dreams were reported as bothersome and primary reasons for treatment failure (15.2% of treatment failure group). Efficay: retrospective data on consecutive patients receiving treatment. Outcome variable continuous abstinence at 52 weeks. For the combination therapy of VAR and BUP the success rate was higher than for VAR monotherapy; 55% vs 32%, OR 3.21.

**Reference study 5**

Combination VAR/BUP treatment benefits highly dependent smokers in an adaptive smoking cessation paradigm (Rose and Behm, 2017). N= 122+52, RCT. This was a randomized double-blind, parallel group, adaptive treatment trial that identified 222 cigarette smokers not responding to pre-quit nicotine patch treatment, that received VAR/BUP or VAR/placebo for 12 weeks. Combination treatment was more efficacious than VAR alone among highly dependent male smokers. Overall, both treatments were well tolerated; 11.5 % required dose reductions in the VAR/BUP group and 24.8% in the VAR group. The incidence of AE did not differ between the groups. The most common AE in the VAR/BUP group were vivid dreams 14.9%, irritability (9.2%), dry mouh (9.2%) and insomnia (8.0%). Seven participants (4 in the combination arm and 3 in the VAR arm) dropped out due to AEs likely related to treatment. Male smokers and more highly dependent smokers showed the greatest therapeutic response. It was not reported that AUD was a feature for exclusion. Efficacy: outcome variable continuous abstinence at week 8-11. For highly dependent smokers (FTND score 6 and more, cigarettes 20 and more), but not lower dependency smokers, combination therapy was superior to varenicline monotherapy in increasing abstinence; OR 3.14.

**Reference study 6**

Depressive symptoms among patients receiving VAR and BUP for smoking cessation (Hong et al., 2015). N=385. This was a subsequent analysis of the Phase III trial conducted by Ebbert (Ebbert et al., 2014), study 3 above. It was reported that combination therapy with VAR and BUP increased depressive symptoms over the first 2 weeks but this did not last beyond 4 weeks.

**Reference study 7**

In the most recent published RCT with the combination of VAR and BUP, contrary to previous studies, the addition of BUP to VAR did not increase the efficacy on smoking abstinence (Cinciripini et al., 2018). Compared to placebo, the group receiving both VAR and BUP showed decreased appetite and increased dry mouth, insomnia, dysgeusia, creatinine and edema. It should be noted that pre-existing medical conditions may have caused elevations of creatinine, edema and dysgeusia. One SAE, which involved a suicide attempt, was rated as possibly related to VAR, although there were other factors surrounding the event. Efficacy outcome variable: prolonged abstinence at 12 months. Odds Ratio for VAR monotherapy, compared to placebo was 6.66. Odds ratio for combination treatment VAR and BUP, compared to placebo was 6.06. There was no BUP monotherapy group. The combination therapy was superior to VAR monotherapy only in smokers with 6 or more scoring at the Fagerström Test for Nicotine Dependency (35% vs 16%, OR 2,81).

**Varenicline and bupropion interactions with alcohol**

VAR is already tested in subjects with AUD and the interaction with alcohol was found not to be clinical significant (Litten et al., 2013;de Bejczy et al., 2015). In the clinical trials with the combination VAR and BUP, AUD patients have been excluded. However, alcohol-consuming patients were not excluded and alcohol consumption was not an exclusion criteria. No adverse interactions were described (reference studies 1-7 above). Drugs of abuse, including alcohol, enhance DA levels in the brain. Both BUP and VAR increase DA levels in the reward region of the brain. However, BUP and VAR have a slow onset of action and a much lower increase in DA levels and are not judged to have an addictive potential. According to Läkemeldesverket, no reports of addiction to VAR or BUP have been reported (oral communication). The combination of alcohol, VAR and BUP have a theoretical risk of enhancing alcohols effects and producing a high/false sense of well-being (Volkow and Swanson, 2003), although no such effects were observed in the VAR studies on alcohol. The known increase risks for seizures have been minimized by the exclusion criteria of Epilepsy and alcohol seizures (within 5 years) and need of detoxification. The risk for worsening symptoms of other psychiatric disorders has been minimized by the exclusion criteria. Common side effects of VAR and BUP include e.g. insomnia, dizziness, nausea, all of which are associated with alcohol consumption. These symptoms could possibly be more frequent in the case of VAR, BUP and alcohol in combination.

**Risks associated with study specific examinations and sampling**

Venous blood will be collected for investigation of eligibility measures and/or outcome measures and/or safety measures at every study visit (9 in total) by professional medical staff (See table 2, section 3.1 for specific investigations). The blood will be drawn from the upper or lower arm. The total amount of blood taken during the whole study will be 245 mL. It is rare for a blood test to result in serious complications but there is a small risk for complications like infections, excessive bleeding, bruising, fainting and dizziness and hematomas. *Action taken to control or minimize above described risks*. Infection control during blood sampling: the medical staff is highly trained and is required to follow the regulations regarding the disposal and storage of needles and blood samples. In rare cases, a wound accurs that become red and swollen, and if so the subject is referred to appropriate medical care. Bleeding and bruising control: if bleeding occurs this will be stopped quickly by placing a cotton wool pad or gauze patch on the wound. Mild pressure on the site after removal of the needle will minimize the bleeding which will reduce the risk of bruising*. Fainting and dizziness control.*Some people experience dizziness during or after a blood test. If the subject is feeling faint before or during a blood test, he/she is offered to lie down on the examination bench. This will increase blood flow to the brain and counteract fainting.

## Definitions

### Adverse Event (AE)

An AE is any untoward medical occurrence in a patient or clinical investigation subject administered a medicinal product and which does not necessarily have a causal relationship with this treatment. An AE can be any unfavorable and unintended sign (including an abnormal laboratory finding), symptom, or disease temporally associated with the use of the IMP, whether or not related to the IMP.

**Causality**

The Investigator is responsible for determining whether there is a causal relationship between an AE and the use of a medicinal product.

All AEs are categorized either as unrelated, possibly related or related, as defined below:

- Unrelated: the AE is not reasonably in relation to the use of the IMP, or another cause can itself explain the occurrence of the event.
- Possibly related: the AE may be explained by an effect of the IMP and the onset is reasonable in relation to the use of the medicinal product, however there is insufficient information to determine the likelihood of this possibility.
- Related: the AE is most likely explained by the IMP and the onset is reasonable in relation to the use of the medicinal product (see 7.2.2., ADR).

**Severity**

In addition to assessing the relationship of the administration of the IMPs to adverse events, an assessment is required of the intensity (severity) of the event. The following over-all classification will be used:

- Mild: An adverse event which is relatively mild and transient in nature, but can be an annoyance, and does not interfere with normal activities.
- Moderate: An adverse event which may be uncomfortable but is not hazardous to health. It may be sufficiently discomforting to interfere with normal activities but does not completely prevent them.
- Severe: An adverse event which is incapacitating and/or is a hazard to the subject

**Definition of action taken**

Action taken will be defined as

- None. No action will be taken
- Due to the AE, interruption in IMP administration
- IMP discontinued. Due to the AE, the patient’s IMP administration will be discontinued.
- Trial discontinuation
- Others (like referrals)

**Definite event outcome at resolution or End of Trial**

- Resolved without sequelae
- Resolved with sequelae
- Not resolved
- Fatal
- Unknown (if lost to follow up)

### Adverse Drug Reaction (ADR)

Adverse Drug Reactions are all AEs considered to have a reasonable causal relationship to the trial medication, as judged by a medically qualified Investigator or the Sponsor. The expression reasonable causal relationship means that there is evidence or argument to suggest a causal relationship.

### Serious Adverse Event (SAE)

A SAE is defined as an untoward and unintended response to a study drug (AE) that meets one of the following serious criteria:

1. Lead to death
2. Is life-threatening
3. Requires in-patient hospitalization or prolongations of existing hospitalization
4. Results in persistent or significant disability/incapacity
5. Is a congenital anomaly/birth defect
6. Require intervention to prevent permanent impairment or damage

Medical and scientific judgement should be exercised in deciding whether an event is serious. For example, important medical events that may not be immediately life threatening or result in death or hospitalization, but may jeopardize the subject or may require intervention to prevent one of the other outcomes listed in the definition above. These should also usually be considered SAEs.

### Suspected unexpected serious adverse reaction (SUSAR)

A suspected unexpected serious adverse reaction (SUSAR) is a SAE of which nature or severity is not consistent with the applicable product information, that is, the SmPCs for VAR and BUP. A SUSAR may be related or unrelated to the study drug, but is unexpected. The responsibilities and requirements concerning SUSAR reporting are determined by Directive 2001/20/EC and a detailed guidance document (CT-3).

If the natural course of the disease usually causes certain symptoms, hospitalizations, etc., these will not be reported as incident or SUSAR (for example hospitalization due to alcohol intoxication).

## Reporting

### Adverse Event (AE)

All AEs occurring during the trial that are observed by study personell or reported by the subject, will be recorded by the study coordinator on the CRF, whether or not attributed to trial medication. All reported AEs will be judged and signed by responsible medical doctor. The following information will be recorded: description, date of onset and end date, severity, assessment of relatedness to trial medication and action taken. Follow-up information should be provided as necessary.

The Investigator’s clinical judgment will decide whether or not an AE is of sufficient severity to require the participant’s removal from treatment. A subject may also voluntarily withdraw from treatment due to what he or she perceives as an intolerable AE. At pre-term termination due to AE, the participant must undergo an end-of-trial assessment and be given appropriate care under medical supervision until symptoms cease, or the condition becomes stable.

### Adverse Drug Reaction (ADR)

AEs considered related to the trial medication as judged by the medically qualified investigator or the Sponsor will be followed either until resolution, or the event is considered stable.

### Serious Adverse Event (SAE)

All SAEs must be reported on the SAE Reporting Form to the Sponsor or delegate within 24 hours of Site study team becoming aware of the SAE.

### Suspected Unexpected Serious Adverse Reaction

All Suspected Unexpected Serious Adverse Reactions (SUSARs) will be reported by the Sponsor to the relevant Competent Athority (CA), to the Ethics Committee and all participating Investigators. Fatal and life-threatening SUSARs, this will be reported no later than 7 calendar days after the Sponsor or delegate is first aware of the reaction. Any additional relevant information will be reported within 8 calendar days of the initial report. All other SUSARs will be reported within 15 calendar days.

This is an investigator-initiated study where the PI lacks the ability to report directly into the European database of side effects (the EudraVigilance database). The CA, in this case Swedish Medical Products Agency, will provide the service of reporting SUSARS to the EudraVigilance system. SUSARs will be reported by the Sponsor/PI via the CIOMS-form to the CA by sending it to [registrator@mpa.se](mailto:registrator@mpa.se)

### Annual Safety Report

A safety report will be completed by Sponsor once a year and sent to the CA and the Ethics Committee. The document will define the time period reported and summarize all occurred serious events (SAEs and SUSARs). The safety report should also always include a summary assessment of the safety of subjects that are still included in the trial and whether the benefit-risk assessment changed since the study was approved.

## Procedures in case of emergency, overdose or pregnancy

VAR and BUP are contraindicated in pregnant or nursing women. Thus pregnancy or nursing infant(s) as well as women of childbearing age not using a contraceptive are exclusion criteria and accordingly not included in the study.

If an emergency, an overdose or pregnancy occurs during the course of the study, the subject must be withdrawn from study medication immediately and the incidence notified to the Sponsor without delay. The patient will be followed up during the entire course of the sequele. In case of pregnancy, the pregnancy and lactation period, with prenatal and neonatal outcomes recorded even if completely normal and without Adverse Events

**Emergency phone number**

Emergencies including overdoses will be handled by an Emergeny card and a safety call center. The central call center will be serviced by professions that can provide core information to take immediate action for each emergency. The call center will be serviced 24 hrs a day by the investigational staff at the coordinating center.

**Patient Emergeny Card**

The study subject should carry an emergency card during the trial. The card contains information about the medications in the study, investigators responsible for the study and contact information. In case of an emergeny and when breakning the code is of medical importance, the responsible medical doctor can call the call center for breaking the specific code of the study participant (see Appendix 3 for Patient Emergency Card).

# Statistics

## Sample size calculation

The power analysis is based on two independent primary hypotheses;

- B-PEth differs significantly between VAR and BUP in combination and alone versus placebo
- Proportion of heavy drinking days (HDD) differ significantly between VAR and BUP in combination and alone versus placebo

Concerning B-PEth, we assume that we can use ordinary t-tests based on normal distribution, with α=0.025, 1-β=0.80 and using a standard deviation of 0.8 (previous results from (de Bejczy et al., 2015). Recruiting 320 patients, randomized in 4 groups of 80 each, we get a detectable effect of 0.35 umol/l and a Cohen’s d of 0.44.

With regards to HDD, we assume that we can use ordinary t-tests based on normal distribution, with α=0.025, 1-β=0.80, using a standard deviation of maximum 0.35 (previous results from (Litten et al., 2013). Recruiting 360 patients, randomized in 4 groups of 90 each, we get a detectable effect of 15% and a Cohen’s d of 0.43.

Hence, a sample size of approximately 360 patients should be randomized.

Drop-out rate

Based on prior studies (de Bejczy et al., 2015) with similar design and outcome, the drop-out rate is estimated to approx. 20 % between screening and randomization and approx. 6.5% for randomization to subjects included in modified ITT. To balance the drop-out, 380 subjects are estimated for randomization to reach 360 subjects in the modified ITT.

## Statistical analysis

The main hypotheses that will be tested in a hierarchial fashion are:

- Combined treatment with VAR and BUP reduces blood concentrations of B-PEth from baseline and significantly more than double-placebo, VAR or BUP alone.
- VAR reduces B-PEth from baseline and significantly more than double-placebo.

The ANCOVA analysis will compare the mean values of the variable in question for the different treatment groups hierarchially. Regulatory agency will consider only one specific primary objective and since the two primary endpoints fulfill regulatory requests independently and separately, no adjustment for multiplicity has been done. As testing between treatments are performed in a hierarchial fashion, no adjustment for multiple testing has been done.

The SAS® software, version 9.2 or higher, will be used to produce all of the statistical outputs.

All efficacy and safety variables will be presented using descriptive statistics and graphs as appropriate.

Safety measures will be summarized descriptively using the safety analysis set. Continuous variables will be presented with descriptive statistics (n, mean, standard deviation [SD], median, min, max), within treatment group. Categorical variables will be summarized by treatment group in frequency tables (number of patients and percentage). The denominators for all percentages will be defined as the number of non-missing values for each variable within the analysis set. Percentages will be presented with 1 decimal place.

**Primary efficacy variables**

- **B-PEth**

One of the primary outcomes will be level of PEth measured in blood.

The primary efficacy variable, B-PEth, is calculated as the mean value over the 8-week steady state active treatment period visit 4 – visit 8 (Day21-Day77) compared to baseline. The data is analyzed as the within patient difference. Day78-Day91 is omitted in the analysis period due to the tendency of drop-out and non-compliance at the end of a study period. Day 0-Day21 is omitted in the analysis due to titration of IMP and optimal timeframe of B-PEth analysis.

Additionally, B-PEth-level analysis compared to baseline levels will also be performed for Day7, Day 21, Day 35, Day 49, Day 63 and Day 77. B-PEth has a well-documented declining pattern and hence it follows that the mean value during the treatment period will be a sufficient statistic for the post-treatment level. B-PEth will be collected initially at the screening visit and onwards from randomization visit every 2nd week during the study (except for the first visit, which is one week after randomization). An analysis with regards to B-PEth, will be performed using an analysis of covariance (ANCOVA) using the mean value of B-PEth (day21-day77) within patient, as the dependent variable. Adjustments for following covariates will be performed: treatment group, center, nicotine use (Yes/No) and family history positive, FHP (Yes/No).

The ANCOVA model will have fixed effects for treatment and center and subject, nicotine use and FHP will be entered as random effect.

Baseline B-PEth will be used as a covariate in the analysis.

- **Heavy Drinking Days (HDD)**

The additional main outcome will be alcohol consumption as measured by Heavy Drinking Days (HDD) using the Time Line Follow Back (TLFB) procedure. The number of heavy drinking days HDD by 14 days is defined as a mean over the 8-week steady state active treatment period (Day 21 -Day 77) compared to baseline. A HDD is defined in two ways; as ≥60 grams for men and ≥40 for women according to EMA’s guideline and as ≥70 grams for men and ≥56 grams for women according to FDA’s guideline on the development of medicinal products for the treatment of alcohol dependence. The baseline mean of heavy drinking days by 14 days, is obtained at the screening visit, where the number of heavy drinking days over the last 30 days is registered.

The number of heavy drinking days, “HDD 21-77”, is the total number of heavy drinking days divided by 4 (8weeks/2), in order to get a 14 day-period measurement. The maximum total number of days measured is 56 days. Additionally, HDD analysis compared to baseline levels will also be performed for Day7, Day 21, Day 35, Day 49, Day 63 and Day 77 and parts of the 8 week steady state active treatment period.

Adjustment for the covariates treatment group, smoking (Yes/No), family history positive, FHP, (Yes/No) and center will be performed. Baseline alcohol consumption will be used as a covariate in the analysis. The ANCOVA model will have fixed effects for treatment and center and subject, nicotine use and FHP will be entered as random effect.

## Definition of analysis sets

Three analysis sets will be used: Modified Intention-To-Treat (mITT), Per Protocol (PP) and Safety. The primary and secondary efficacy endpoints will be estimated using ITT and PP analysis sets separately. The safety endpoints will be analyzed on the safety analysis set.

## Modified Intention-to-treat (mITT) analysis set

The mITT analysis set will include all trial subjects who complete screening visit and randomization visit and report that they have taken at least one dose of the randomized IMPs. Subjects will be analyzed according to their randomized IMPs.

## Per-protocol (PP) analysis set

The PP analysis set constitutes all subjects from the ITT population who are considered to be completers. A completer is defined as a subject who:

- has taken the IMP at least 80% of the planned number of days of the active treatment period
- fulfill the visit milestones, where the visit milestones are met by subjects who have:
- taken part and completed screening visit, Day 0, Day 21, Day 49 and Day 77
- successful B-PEth analyses at screening visit, Day 0, Day, 21, Day 49 and Day 77
- completed TLFB data at screening visit, Day 0, Day, 21, Day 49 and Day 77

In case of violation of any of the above-mentioned criteria the subject will not be included in the PP analysis set for the specific outcome variable. Subjects will be analyzed according to their randomized study drug.

## 3) Safety set

The safety analysis set includes all subjects who report that they have taken at least one dose of randomized study drug, and for whom any post-dose data is available up until Day 77.
Patients will be analyzed according to the actual treatment.

**Compliance**

Adherence to the study drug regimen will be analyzed by pill count and by plasma levels of IMP1 and IMP2 at visit 4 and visit 6.

### Randomization issues

Mistakenly randomizing a subject who does not meet the inclusion/exclusion criteria will be considered a protocol violation. Any such subject will not be included in the analysis sets.

**Handling of missing data**

Handling of missing data in the alcohol consumption data as measured by TLFB. In a blind data review, missing data will be assessed. If found appropriate, since we expect that the missing data are not at random, due to the tendency of alcohol patients to have a drinking pattern, we will handle the missing data not by last observation carried forward (LOCF), but instead by an imputation taking the subjects pattern into account. The missing day will be imputed by the data from the same weekday in the weeks before and after, if possible. To adjust for changes in alcohol consumption over time, the weeks closest to the missing day will have a greater weight than the weeks more distant from the missing day.

If a subject drops out from the study in early treatment, the baseline observation will be carried forward. If the subject drops out late in the treatment period the last observed 7 days will be carried forward, if there are missing days, these will be imputed as described above. This model allows for the probability that a longer treatment period will have a greater impact on the alcohol consumption. For all other variables missing data will be considered missing and no imputation will be computed.

All statistical concerns and estimations are described in a separate statistical protocol, the Statistical Analysis Plan (SAP).

# Data Management

## Recording of data

An electronic CRFs (eCRF) will be custom-made to the trial by a company appointed by the Sponsor. The trial-specific eCRF will be designed and used to record all the information required by the protocol to be reported on each visit for each trial subject. If, and only if using eCRFs is not possible, a paper CRF may be used. CRFs in paper forms will be available on each site and may be used as source documents if the use of the eCRFs is not possible.

The Investigator will ensure that study specific data are recorded in the eCRF in a timely manner according to instructions eCRF (also the transferal of source data in paper form, if direct incorporation into eCRF is not possible, .e.g. laboratory analysis). The Investigator and site personnel will be instructed by the Sponsor in the use of the eCRF. The Investigators must verify that all data entries in the eCRF are accurate. The Investigator and delegated site personell must make all eCRF entries by using a subject-unique login identity. If necessary, the Investigator will be requested to confirm or make corrections, or enter additional or missing data as required bu Queries created in the eCRF. The Investigator will also ensure that any corrections in the CRF, as specified in the study protocol, are in accordance with the instructions provided. The Investigator ensures the accuracy, completeness and timeliness of the data recorded by signing the completed CRF.

The Investigator will have access to all data captured during the trial via the eCRF that is supplied through a secure Internet access. At the end of the trail, the Investigator will also receive a certified copy of all data captures for the study subject.

## Source data and source documents

In accordance with ICH E6, source data is defined as "all information in original records and certified copies of original records or clinical findings, observations, or other activities in the clinical trial necessary for the reconstruction and evaluation of the trial”. Source data are contained in source documents (original records or certified copies). Source documents are defined as original documents, data and records. This comprises all hospital records, clinical and office charts connected to the study.

Source documents in the trial comprise:

- eCRF
- Paper copy of CRF in case direct incorporation into eCRF is not possible
- Medical journals and hospital records
- Informed consent form
- Paper laboratory analysis and laboratory notes
- Recorded data from automated instruments, e.g. blood pressure data
- Saliva sticks, e.g. cotinine analysis
- Urine dip sticks with drug indentification an pregancy results
- Study specific spreadsheets and templates
- Study specific regulatory documentation
- SUSAR and SAE documents
- IMP dispensing records and drug accountability records
- Drug accountability and drug dispensing records
- Temperature logs

The Investigator must maintain source documents for each subject in the study. A source data verification log will be included in the Investigator Study File (ISF). The Investigator must ensure that all source documents, i.e. eCRF, progress notes, copies of laboratory and medical test results must be available for inspection and assessment by the Sponsor/coordinating centre, the monitor and/or regulatory authorities.

## Data storage and management

**Storage**

All data will be recorded, handled and stored in a way that allows accurate reporting, interpretation and verification. After termination of the trial, all source data (paper and electronical files) including ICF, a copy of the completed CRF, original protocol with amendments and the final report will be stored at Sahlgrenska University Hospital, Psychiatry Archive/Addiction Biology Unit, Blå Stråket 15, floor -1, Gothenburg for a minimum period of 15 years in accordance with Swedish regulation/law (Chapter 10, 3 § in LVFS 2011:19).

**Data management**

Data management will be co-ordinated by the Principal Investigator/

Sponsor. The Sponsor’s designated investigational staff will review the data entered into the eCRFs for completeness and accuracy and will instruct the study sites to make any required corrections or additions, by queries issued electronically in the eCRF. Designated site staff is required to respond to the query and confirm or correct the data. Trial data not recorded in the eCRF (e.g. safety and outcome laboratory data) will be transferred to the eCRF system at predefined intervals in a predefined data structure form. Medical history and AE will be coded using the Medical Dictionary for Regulatory Activities (MedDRA). After End- of-Study, the occurrence of any protocol deviations will be determined. After these actions have been completed and the database has been declared complete and accurate, it will be locked and available for data analysis.

**Management of laboratory data**

Laboratory data will be handled according to the General Data Protection Regulation (EU) 2016/679 (GDPR). The primary outcome variable B-PEth will be analyzed at the Central Laboratory assigned to the Coordinating site. The central lab uses relevant quality systems according to ISO 17024.

The blood samples for B-PEth analysis will be transported to Coordinating site in accordance to Hosptial Policy in accordance to required time and temperature conditions. All other analyses, i.e. blood samples for safety measurements and the secondary outcome measures CDT and GGT will be analyzed on site by each site’s respective primary blood bank/ by the Investigator appointed local laboratory. Blood samples for analysis of IMP concentrations will be frozen and stored at respective study site. Laboratoy data will be incorporated into eCRF by investigational staff only after approval by medical doctor. When all data is received, alla data problems have been solved and quality control checks have been performed, the study database is considered clean and ready to be locked.

## Study complicance

Study compliance is the adherence to all trial related requirements, to GCP and to regulatory rules and regulations. Non-complicance will lead to prompt action by the Sponsor to secure compliance and may result in closure of the site and notification of the regulatory authorities. Sites with a very low recruitment rate or which persistently violate the protocol may be judged by the Sponsor to stop further recruitment at once or to stop participation in the trial.

# Quality Control and Quality Assurance

## Monitoring

The study will be monitored by independent monitor(s) according to GCP guidelines. The study monitor will be appointed by the Sponsor and will be appropriately trained and informed about the nature of the study, subject written information, GCP and applicable regulatory requirements. The monitor’s qualifications will be documented. The monitor should follow the Sponsor’s established written SOPs as well as those procedures that are specified by the sponsor in the monitoring plan.

The study will be monitored before, during and after the study period. Before starting the clinical trial, all centers will have a telephone/web-based or physical initiation meeting with presentation of the study, study procedures and documentation. During the study period, monitors will have regular contact with the participating clinics to ensure that the trial is conducted in compliance with the protocol and applicable regulatory requirements. The monitors will also provide information and support to the centers. The number of monitoring visits will be limited, and unless no specific problems occur the main part of the monitoring will be centralized by regular checks of the data quality in the study database. When visiting the clinics, the monitor will review source documents for verification of consistency with the study data recorded in CRF, source data verification. An agreement between the institution and the monitor will be set up to allow the monitor´s access to the patient journals. The monitor will submit a written report to the sponsor after each trial-site visit. The monitoring will be further described in the monitoring plan.

## Audits and inspections

Authorized representatives of the sponsor, a Competent Authority (CA) or an Ethics Committee may perform audits or inspection at the center, including source data verification. The Investigator must ensure that all source documents are accessible for auditing and inspection. The purpose of an audit or inspection is to systematically and independently examine all study-related activities and documents, to determine whether these activities were conducted, and data were recorded, analyzed and accurately reported according to the protocol, GCP and the applicable regulatory requirements.

# Ethics

The study will be performed in accordance with the study protocol, with the latest version of the Declaration of Helsinki, in acoordance with GCP principles, that is ICH-GCP E6(R2), and applicable regulatory requirements i.e. “Läkemedelsverkets föreskrifter” (LVFS 2011:9) . The study will be conducted after approval from CA (the Swedish Medical Product Agency) and the Etics committee (EC) (See 11.1).

## Ethics committee

The final study protocol, including the final version of the Informed Consent Form (Appendix 2) and other information given to subjects e.g. advertisements, must be approved or given a favorable opinion in writing by an EC as appropriate. The Principal Investigator is responsible for informing the EC of any amendment to the protocol, in accordance with local requirements. Progress reports and notifications of any serious and unexpected adverse drug reactions will be provided to the EC according to local regulations and guidelines.

## Informed consent

The Investigator at each center will ensure that the subject is given full and adequate oral and written information about the nature, purpose and possible risks and benefits of the study. Subjects must also be notified that they are free to discontinue from the study at any time. The subject should be given the opportunity to ask questions and allowed time to consider the information provided. The subject’s signed and dated informed consent must be obtained before conducting any procedure specifically for the study. The monitor(s), the auditor(s), and the CA(s) will be granted direct access to the subject’s original medical records for verification of clinical trial procedures and/or data, without violating the confidentiality of the subject, to the extent permitted by the applicable laws and regulations and that, by signing a written informed consent form, the subject’ is authorizing such access. The original, signed ICF must be stored in the Investigator’s Study File. A copy of the signed ICF will be given to the subject. See Appendix 3 for ICF.

If a protocol amendment requires a change to the ICF, the EC must approve modifications that lead to a revised ICF before the revised form is used.

## Subject data protection

The Informed Consent Form will incorporate wording that complies with relevant data protection, the EU General Data Protection Regulation (GDPR) 2016/679, privacy legislation. Pursuant to this wording, subjects will authorize the collection, use and disclosure of their study data by the Investigator and by those persons who need that information for the purposes of the study.

The ICF will explain that study data will be stored in a computer database, maintaining confidentiality in accordance with national data legislation. The identity of the subject is not disclosed in the stored data; all data computer processed by the sponsor will be stored by an alphanumerical code on the basis of “Study Code/Subject ID/Initials”, for example BO8-1, 1001, FL.

The conduct of the trial is subject to supervision. The ICF will explain that for data verification purposes, authorized representatives of the sponsor, a regulatory authority or an EC may require direct access to parts of the hospital or practice records relevant to the study, including subjects’ medical history.

## Insurances

The study participants are covered by the Swedish Patient Injury Act and the Swedish Pharmaceutical Insurance (Läkemedelsförsäkringen, se http://lff.se/).

# Protocol Deviations and Amendments

Changes to the signed protocol during the trial are only possible through approved protocol amendments and with the agreement of all responsible persons. Details of non-substantial amendments are to be clearly noted in the amended protocol. A change that concerns; a new trial site, new principal investigator and or a new informed consent form should only be submitted to the concerned EC.

If an amendment substantially alters the trial design, (e.g. change of; main purpose of the trial, primary/secondary variable, measurement of primary variable, investigational product, or dosing according to ‘Väsentliga ändringar i protokollet’, LVFS 2011:19), the concerned Ethics Committee and CA must be informed and asked for its opinion/approval prior to implementation of amended protocol, as to whether a full re-evaluation of the ethical aspects of the study is necessary. This should be fully documented. The EU form ‘Substantial Amendment Notification Form’ must be used.

The Investigator must not implement any deviation from, or change to the protocol, without discussion with, and agreement by the Sponsor and prior review and documented approval/favorable opinion of the amendment from the relevant ethics committee and competent authority. An exception is where it is necessary to eliminate an immediate hazard to study subjects, or where the change(s) involves only logistical or administrative aspects of the study (e.g. change in monitor(s), change of telephone numbers).

# Report and publications

No later than 90 days after End of Study the Sponsor will notify CA by using the EU form Declaration of End of Trial Notification. In case of a premature termination of the trial, CA will be notified within 15 days.

After completion of the study, the results will be analyzed and a clinical trial report (CTR) including all efficacy and safety results of the study will be prepared. Within one year after the End of study (according to the protocol), the Sponsor will submit the final CTR to the Ethics Committee and the CA. The CA will be reported through the EudraCT database. The CTR will be created according to the standards of the ICH Guideline for Structure and Content of Clinical Study Reports.

The results from the study will be presented at national and international scientific meetings and will be published in peer reviewed scientific journals. The scientific manuscript/abstracts/posters will fulfil ICMJE (International committee for medical journal editors) requirements and report the EduraCT number in the abstract. No details on specific subjects or information that may be derived from any specific subject or identify any specific subject will be made public. Data will be analysed at group level only. The trial will be registered on [www.clinicaltrials,gov](http://www.clinicaltrials,gov) as soon as the trial has been appoved by the authorities and the local ethical committee.

# Study Timetable

First-Subject-In: March 2019.

Last-Subject-Out: June 2022

Analysis and CTR preparation: July 2022- June 2023

CTR finalized and reported: June 2023

## Definition of “End of study”

End-of-study is defined as last visit of the last subject (LVLS). The sponsor will notify the concerned Ethics Committee and the CA of the end of the study within a period of 90 days. First-Subject-In is scheduled to March 2019. Last-Visit-Last-Patient is scheduled to June 2022.

# List of References

Aitken, R.C. (1969). Measurement of feelings using visual analogue scales. *Proc R Soc Med* 62**,** 989-993.

Anthenelli, R.M., Benowitz, N.L., West, R., St Aubin, L., Mcrae, T., Lawrence, D., Ascher, J., Russ, C., Krishen, A., and Evins, A.E. (2016). Neuropsychiatric safety and efficacy of varenicline, bupropion, and nicotine patch in smokers with and without psychiatric disorders (EAGLES): a double-blind, randomised, placebo-controlled clinical trial. *Lancet* 387**,** 2507-2520.

Aradottir, S., Asanovska, G., Gjerss, S., Hansson, P., and Alling, C. (2006). PHosphatidylethanol (PEth) concentrations in blood are correlated to reported alcohol intake in alcohol-dependent patients. *Alcohol Alcohol* 41**,** 431-437.

Aubin, H.J., Karila, L., and Reynaud, M. (2011). Pharmacotherapy for smoking cessation: present and future. *Curr Pharm Des* 17**,** 1343-1350.

Babor Tf, H.-B.J., Saunders Jb, Monteiro Mg. (2001). AUDIT. The Alcohol Use Disorders Identification Test. Guidelines for use in primary care. Geneva: World Health Organisation; 2001. WHO/MSD/MSB/01.6a.

Berman, A.H., Bergman, H., Palmstierna, T., and Schlyter, F. (2005). Evaluation of the Drug Use Disorders Identification Test (DUDIT) in criminal justice and detoxification settings and in a Swedish population sample. *Eur Addict Res* 11**,** 22-31.

Bien, T.H., and Burge, R. (1990). Smoking and drinking: a review of the literature. *Int J Addict* 25**,** 1429-1454.

Blomqvist, O., Ericson, M., Johnson, D.H., Engel, J.A., and Soderpalm, B. (1996). Voluntary ethanol intake in the rat: effects of nicotinic acetylcholine receptor blockade or subchronic nicotine treatment. *Eur J Pharmacol* 314**,** 257-267.

Bohn, M.J., Babor, T.F., and Kranzler, H.R. (1995). The Alcohol Use Disorders Identification Test (AUDIT): validation of a screening instrument for use in medical settings. *J Stud Alcohol* 56**,** 423-432.

Cahill, K., Stead, L.F., and Lancaster, T. (2012). Nicotine receptor partial agonists for smoking cessation. *Cochrane Database Syst Rev***,** CD006103.

Casey, E.R., Scott, M.G., Tang, S., and Mullins, M.E. (2011). Frequency of false positive amphetamine screens due to bupropion using the Syva EMIT II immunoassay. *J Med Toxicol* 7**,** 105-108.

Cinciripini, P.M., Minnix, J.A., Green, C.E., Robinson, J.D., Engelmann, J.M., Versace, F., Wetter, D.W., Shete, S., and Karam-Hage, M. (2018). An RCT with the Combination of Varenicline and Bupropion for Smoking Cessation: Clinical Implications for Front Line Use. *Addiction*.

De Bejczy, A., Lof, E., Walther, L., Guterstam, J., Hammarberg, A., Asanovska, G., Franck, J., Isaksson, A., and Soderpalm, B. (2015). Varenicline for treatment of alcohol dependence: a randomized, placebo-controlled trial. *Alcohol Clin Exp Res* 39**,** 2189-2199.

De Bejczy, A., and Soderpalm, B. (2015). The effects of mirtazapine versus placebo on alcohol consumption in male high consumers of alcohol: a randomized, controlled trial. *J Clin Psychopharmacol* 35**,** 43-50.

Ebbert, J.O., Croghan, I.T., Sood, A., Schroeder, D.R., Hays, J.T., and Hurt, R.D. (2009). Varenicline and bupropion sustained-release combination therapy for smoking cessation. *Nicotine Tob Res* 11**,** 234-239.

Ebbert, J.O., Hatsukami, D.K., Croghan, I.T., Schroeder, D.R., Allen, S.S., Hays, J.T., and Hurt, R.D. (2014). Combination varenicline and bupropion SR for tobacco-dependence treatment in cigarette smokers: a randomized trial. *JAMA* 311**,** 155-163.

Einhorn, L.C., Johansen, P.A., and White, F.J. (1988). Electrophysiological effects of cocaine in the mesoaccumbens dopamine system: studies in the ventral tegmental area. *J Neurosci* 8**,** 100-112.

Feltmann, K., Fredriksson, I., Wirf, M., Schilstrom, B., and Steensland, P. (2016). The monoamine stabilizer (-)-OSU6162 counteracts downregulated dopamine output in the nucleus accumbens of long-term drinking Wistar rats. *Addict Biol* 21**,** 438-449.

Ferry, L., and Johnston, J.A. (2003). Efficacy and safety of bupropion SR for smoking cessation: data from clinical trials and five years of postmarketing experience. *Int J Clin Pract* 57**,** 224-230.

Gershon, A.S., Campitelli, M.A., Hawken, S., Victor, C., Sproule, B.A., Kurdyak, P., and Selby, P. (2018). Cardiovascular and Neuropsychiatric Events after Varenicline Use for Smoking Cessation. *Am J Respir Crit Care Med* 197**,** 913-922.

Gnann, H., Weinmann, W., and Thierauf, A. (2012). Formation of phosphatidylethanol and its subsequent elimination during an extensive drinking experiment over 5 days. *Alcohol Clin Exp Res* 36**,** 1507-1511.

Hahn, J.A., Dobkin, L.M., Mayanja, B., Emenyonu, N.I., Kigozi, I.M., Shiboski, S., Bangsberg, D.R., Gnann, H., Weinmann, W., and Wurst, F.M. (2012). Phosphatidylethanol (PEth) as a biomarker of alcohol consumption in HIV-positive patients in sub-Saharan Africa. *Alcohol Clin Exp Res* 36**,** 854-862.

Hall, B.J., Slade, S., Wells, C., Rose, J.E., and Levin, E.D. (2015). Bupropion-varenicline interactions and nicotine self-administration behavior in rats. *Pharmacol Biochem Behav* 130**,** 84-89.

Harrison-Woolrych, M., and Ashton, J. (2011). Psychiatric Adverse Events Associated with Varenicline: An Intensive Postmarketing Prospective Cohort Study

in New Zealand. *Drug Safety*

34 (9)**,** 763-772.

Hartmann, S., Aradottir, S., Graf, M., Wiesbeck, G., Lesch, O., Ramskogler, K., Wolfersdorf, M., Alling, C., and Wurst, F.M. (2007). Phosphatidylethanol as a sensitive and specific biomarker: comparison with gamma-glutamyl transpeptidase, mean corpuscular volume and carbohydrate-deficient transferrin. *Addict Biol* 12**,** 81-84.

Heather, N., Adamson, S.J., Raistrick, D., Slegg, G.P., and Team, U.R. (2010). Initial preference for drinking goal in the treatment of alcohol problems: I. Baseline differences between abstinence and non-abstinence groups. *Alcohol Alcohol* 45**,** 128-135.

Heinz, A., Siessmeier, T., Wrase, J., Buchholz, H.G., Grunder, G., Kumakura, Y., Cumming, P., Schreckenberger, M., Smolka, M.N., Rosch, F., Mann, K., and Bartenstein, P. (2005). Correlation of alcohol craving with striatal dopamine synthesis capacity and D2/3 receptor availability: a combined [18F]DOPA and [18F]DMFP PET study in detoxified alcoholic patients. *Am J Psychiatry* 162**,** 1515-1520.

Heinz, A., Siessmeier, T., Wrase, J., Hermann, D., Klein, S., Grusser, S.M., Flor, H., Braus, D.F., Buchholz, H.G., Grunder, G., Schreckenberger, M., Smolka, M.N., Rosch, F., Mann, K., and Bartenstein, P. (2004). Correlation between dopamine D(2) receptors in the ventral striatum and central processing of alcohol cues and craving. *Am J Psychiatry* 161**,** 1783-1789.

Hill-Kapturczak, N., Dougherty, D.M., Roache, J.D., Karns-Wright, T.E., and Javors, M.A. (2018). Differences in the Synthesis and Elimination of Phosphatidylethanol 16:0/18:1 and 16:0/18:2 After Acute Doses of Alcohol. *Alcohol Clin Exp Res* 42**,** 851-860.

Hillbom, M., Pieninkeroinen, I., and Leone, M. (2003). Seizures in alcohol-dependent patients: epidemiology, pathophysiology and management. *CNS Drugs* 17**,** 1013-1030.

Hillemacher, T., Bayerlein, K., Wilhelm, J., Frieling, H., Thurauf, N., Ziegenbein, M., Kornhuber, J., and Bleich, S. (2006). Nicotine dependence is associated with compulsive alcohol craving. *Addiction* 101**,** 892-897.

Hodgins, D.C., Leigh, G., Milne, R., and Gerrish, R. (1997). Drinking goal selection in behavioral self-management treatment of chronic alcoholics. *Addict Behav* 22**,** 247-255.

Hong, A.S., Elrashidi, M.Y., Schroeder, D.R., and Ebbert, J.O. (2015). Depressive symptoms among patients receiving varenicline and bupropion for smoking cessation. *J Subst Abuse Treat* 52**,** 78-81.

Issa, J.S., Abe, T.O., Moura, S., Santos, P.C., and Pereira, A.C. (2013). Effectiveness of coadministration of varenicline, bupropion, and serotonin reuptake inhibitors in a smoking cessation program in the real-life setting. *Nicotine Tob Res* 15**,** 1146-1150.

Jonas, D.E., Amick, H.R., Feltner, C., Bobashev, G., Thomas, K., Wines, R., Kim, M.M., Shanahan, E., Gass, C.E., Rowe, C.J., and Garbutt, J.C. (2014). Pharmacotherapy for adults with alcohol use disorders in outpatient settings: a systematic review and meta-analysis. *JAMA* 311**,** 1889-1900.

Jorenby, D.E., Leischow, S.J., Nides, M.A., Rennard, S.I., Johnston, J.A., Hughes, A.R., Smith, S.S., Muramoto, M.L., Daughton, D.M., Doan, K., Fiore, M.C., and Baker, T.B. (1999). A controlled trial of sustained-release bupropion, a nicotine patch, or both for smoking cessation. *N Engl J Med* 340**,** 685-691.

Kechagias, S., Dernroth, D.N., Blomgren, A., Hansson, T., Isaksson, A., Walther, L., Kronstrand, R., Kagedal, B., and Nystrom, F.H. (2015). Phosphatidylethanol Compared with Other Blood Tests as a Biomarker of Moderate Alcohol Consumption in Healthy Volunteers: A Prospective Randomized Study. *Alcohol Alcohol* 50**,** 399-406.

Kessler, R.C., Adler, L., Ames, M., Demler, O., Faraone, S., Hiripi, E., Howes, M.J., Jin, R., Secnik, K., Spencer, T., Ustun, T.B., and Walters, E.E. (2005). The World Health Organization Adult ADHD Self-Report Scale (ASRS): a short screening scale for use in the general population. *Psychol Med* 35**,** 245-256.

Kohn, R., Saxena, S., Levav, I., and Saraceno, B. (2004). The treatment gap in mental health care. *Bull World Health Organ* 82**,** 858-866.

Koob, G.F., and Volkow, N.D. (2016). Neurobiology of addiction: a neurocircuitry analysis. *Lancet Psychiatry* 3**,** 760-773.

Kornfield, R., Watson, S., Higashi, A.S., Conti, R.M., Dusetzina, S.B., Garfield, C.F., Dorsey, E.R., Huskamp, H.A., and Alexander, G.C. (2013). Effects of FDA advisories on the pharmacologic treatment of ADHD, 2004-2008. *Psychiatr Serv* 64**,** 339-346.

Lesch, O.M., Lesch, E., Dietzel, M., Mader, R., Musalek, M., Walter, H., and Zeiler, K. (1986). [Chronic alcoholism--alcohol sequelae--causes of death]. *Wien Med Wochenschr* 136**,** 505-515.

Li, Z., Shi, H.S., Elis, O., Yang, Z.Y., Wang, Y., Lui, S.S.Y., Cheung, E.F.C., Kring, A.M., and Chan, R.C.K. (2018). The structural invariance of the Temporal Experience of Pleasure Scale across time and culture. *Psych J* 7**,** 59-67.

Litten, R.Z., Ryan, M.L., Fertig, J.B., Falk, D.E., Johnson, B., Dunn, K.E., Green, A.I., Pettinati, H.M., Ciraulo, D.A., Sarid-Segal, O., Kampman, K., Brunette, M.F., Strain, E.C., Tiouririne, N.A., Ransom, J., Scott, C., Stout, R., and Group, N.S. (2013). A double-blind, placebo-controlled trial assessing the efficacy of varenicline tartrate for alcohol dependence. *J Addict Med* 7**,** 277-286.

Mann, K., Bladstrom, A., Torup, L., Gual, A., and Van Den Brink, W. (2013). Extending the treatment options in alcohol dependence: a randomized controlled study of as-needed nalmefene. *Biol Psychiatry* 73**,** 706-713.

Martinotti;, G., Hatzigiakoumis;, De Vita, O., Clerici M., Petruccelli, F., Di Giannantonio, M., and L, J. (2012). Anhedonia and Reward System: Psychobiology, Evaluation, and Clinical Features. *International Journal of Clinical Medicine* 3**,** 697-713.

Mckee, S.A., Harrison, E.L., O'malley, S.S., Krishnan-Sarin, S., Shi, J., Tetrault, J.M., Picciotto, M.R., Petrakis, I.L., Estevez, N., and Balchunas, E. (2009). Varenicline reduces alcohol self-administration in heavy-drinking smokers. *Biol Psychiatry* 66**,** 185-190.

Mills, E.J., Wu, P., Lockhart, I., Thorlund, K., Puhan, M., and Ebbert, J.O. (2012). Comparisons of high-dose and combination nicotine replacement therapy, varenicline, and bupropion for smoking cessation: a systematic review and multiple treatment meta-analysis. *Ann Med* 44**,** 588-597.

Montgomery, S.A., and Asberg, M. (1979). A new depression scale designed to be sensitive to change. *Br J Psychiatry* 134**,** 382-389.

Nutt, D.J., and Rehm, J. (2014). Doing it by numbers: a simple approach to reducing the harms of alcohol. *J Psychopharmacol* 28**,** 3-7.

Potthoff, A.D., Ellison, G., and Nelson, L. (1983). Ethanol intake increases during continuous administration of amphetamine and nicotine, but not several other drugs. *Pharmacol Biochem Behav* 18**,** 489-493.

Prochaska, J.J., Delucchi, K., and Hall, S.M. (2004). A meta-analysis of smoking cessation interventions with individuals in substance abuse treatment or recovery. *J Consult Clin Psychol* 72**,** 1144-1156.

Rehm, J. (2011). The risks associated with alcohol use and alcoholism. *Alcohol Res Health* 34**,** 135-143.

Rehm, J., and Roerecke, M. (2013). Reduction of drinking in problem drinkers and all-cause mortality. *Alcohol Alcohol* 48**,** 509-513.

Rose, J.E., and Behm, F.M. (2014). Combination treatment with varenicline and bupropion in an adaptive smoking cessation paradigm. *Am J Psychiatry* 171**,** 1199-1205.

Rose, J.E., and Behm, F.M. (2017). Combination Varenicline/Bupropion Treatment Benefits Highly Dependent Smokers in an Adaptive Smoking Cessation Paradigm. *Nicotine Tob Res* 19**,** 999-1002.

Rosner, S., Hackl-Herrwerth, A., Leucht, S., Vecchi, S., Srisurapanont, M., and Soyka, M. (2010). Opioid antagonists for alcohol dependence. *Cochrane Database Syst Rev***,** CD001867.

Simon, J.J., Zimmermann, J., Cordeiro, S.A., Maree, I., Gard, D.E., Friederich, H.C., Weisbrod, M., and Kaiser, S. (2017). Psychometric evaluation of the Temporal Experience of Pleasure Scale (TEPS) in a German sample. *Psychiatry Res* 260**,** 138-143.

Smith, Y., and Kieval, J.Z. (2000). Anatomy of the dopamine system in the basal ganglia. *Trends Neurosci* 23**,** S28-33.

Sobell, L.C., and Sobell, M.B. (1992). Timeline Follow-back: A technique for assessing self-reported alcohol consumption. *Measuring Alcohol Consumption: Psychosocial and Biological Methods, Humana Press, New Jersey.***,** pp.41-72.

Socialstyrelsen (2010). Kostnader för alkohol och narkotika, beräkningar av samhällets direkta kostnader. *Available from:* [*http://www.socialstyrelsen.se/Lists/Artikelkatalog/Attachments/17961/2010-3-15.pdf*](http://www.socialstyrelsen.se/Lists/Artikelkatalog/Attachments/17961/2010-3-15.pdf)

Soderpalm, B., and Ericson, M. (2013). Neurocircuitry involved in the development of alcohol addiction: the dopamine system and its access points. *Curr Top Behav Neurosci* 13**,** 127-161.

Soderpalm, B., Lido, H.H., and Ericson, M. (2017). The glycine receptor - a functionally important primary brain target of ethanol. *Alcohol Clin Exp Res*.

Soderpalm, B., Lof, E., and Ericson, M. (2009). Mechanistic studies of ethanol's interaction with the mesolimbic dopamine reward system. *Pharmacopsychiatry* 42 Suppl 1**,** S87-94.

Soyka, M., and Rosner, S. (2010). Emerging drugs to treat alcoholism. *Expert Opin Emerg Drugs*.

Spanagel, R., and Holter, S.M. (2000). Pharmacological validation of a new animal model of alcoholism. *J Neural Transm* 107**,** 669-680.

Steensland, P., Simms, J.A., Holgate, J., Richards, J.K., and Bartlett, S.E. (2007). Varenicline, an alpha4beta2 nicotinic acetylcholine receptor partial agonist, selectively decreases ethanol consumption and seeking. *Proc Natl Acad Sci U S A* 104**,** 12518-12523.

Stewart, S.H., Koch, D.G., Willner, I.R., Anton, R.F., and Reuben, A. (2014). Validation of blood phosphatidylethanol as an alcohol consumption biomarker in patients with chronic liver disease. *Alcohol Clin Exp Res* 38**,** 1706-1711.

Stewart, S.H., Law, T.L., Randall, P.K., and Newman, R. (2010). Phosphatidylethanol and alcohol consumption in reproductive age women. *Alcohol Clin Exp Res* 34**,** 488-492.

Stewart, S.H., Reuben, A., Brzezinski, W.A., Koch, D.G., Basile, J., Randall, P.K., and Miller, P.M. (2009). Preliminary evaluation of phosphatidylethanol and alcohol consumption in patients with liver disease and hypertension. *Alcohol Alcohol* 44**,** 464-467.

Walther, L., De Bejczy, A., Lof, E., Hansson, T., Andersson, A., Guterstam, J., Hammarberg, A., Asanovska, G., Franck, J., Soderpalm, B., and Isaksson, A. (2015). Phosphatidylethanol is superior to carbohydrate-deficient transferrin and gamma-glutamyltransferase as an alcohol marker and is a reliable estimate of alcohol consumption level. *Alcohol Clin Exp Res* 39**,** 2200-2208.

Varga, A., Hansson, P., Johnson, G., and Alling, C. (2000). Normalization rate and cellular localization of phosphatidylethanol in whole blood from chronic alcoholics. *Clin Chim Acta* 299**,** 141-150.

Weiss, F., Parsons, L.H., Schulteis, G., Hyytia, P., Lorang, M.T., Bloom, F.E., and Koob, G.F. (1996). Ethanol self-administration restores withdrawal-associated deficiencies in accumbal dopamine and 5-hydroxytryptamine release in dependent rats. *J Neurosci* 16**,** 3474-3485.

Who, W.H.O. (2018). Global Status Report on Alcohol and Health.

Wiesbeck, G.A., Weijers, H.G., Lesch, O.M., Glaser, T., Toennes, P.J., and Boening, J. (2001). Flupenthixol decanoate and relapse prevention in alcoholics: results from a placebo-controlled study. *Alcohol Alcohol* 36**,** 329-334.

Wilens, T.E., Adler, L.A., Weiss, M.D., Michelson, D., Ramsey, J.L., Moore, R.J., Renard, D., Brady, K.T., Trzepacz, P.T., Schuh, L.M., Ahrbecker, L.M., Levine, L.R., and Atomoxetine, A.S.U.D.S.G. (2008). Atomoxetine treatment of adults with ADHD and comorbid alcohol use disorders. *Drug Alcohol Depend* 96**,** 145-154.

Vogeler, T., Mcclain, C., and Evoy, K.E. (2016). Combination bupropion SR and varenicline for smoking cessation: a systematic review. *Am J Drug Alcohol Abuse* 42**,** 129-139.

Volkow, N.D., and Swanson, J.M. (2003). Variables that affect the clinical use and abuse of methylphenidate in the treatment of ADHD. *Am J Psychiatry* 160**,** 1909-1918.

Volkow, N.D., Wang, G.J., Begleiter, H., Porjesz, B., Fowler, J.S., Telang, F., Wong, C., Ma, Y., Logan, J., Goldstein, R., Alexoff, D., and Thanos, P.K. (2006). High levels of dopamine D2 receptors in unaffected members of alcoholic families: possible protective factors. *Arch Gen Psychiatry* 63**,** 999-1008.

Volkow, N.D., Wang, G.J., Telang, F., Fowler, J.S., Logan, J., Jayne, M., Ma, Y., Pradhan, K., and Wong, C. (2007). Profound decreases in dopamine release in striatum in detoxified alcoholics: possible orbitofrontal involvement. *J Neurosci* 27**,** 12700-12706.

Wurst, F.M., Thon, N., Aradottir, S., Hartmann, S., Wiesbeck, G.A., Lesch, O., Skala, K., Wolfersdorf, M., Weinmann, W., and Alling, C. (2010). Phosphatidylethanol: normalization during detoxification, gender aspects and correlation with other biomarkers and self-reports. *Addict Biol* 15**,** 88-95.

# Appendix 1

# MÄRKNINGSFÖRSLAG Studie COMB-BO8

**Blister Vecka 1 (dag 1-7).** Upptrappning av dos

Vareniklin/Placebo

| Studie: COMB-BO8  **Läkemedel 1/Placebo** Rand. Nr: XXX  Dag: X  Sponsor/prövare: Bo Söderpalm  Satsnummer: XXXXXXX |
| --- |

X = 1-3, resp. 4-7

För dag 1-3 kommer det vara 1 st 3-kapslar blister. För dag 4-7 kommer det vara 2 st 4-kapslar blister

Bupropion/Placebo

| Studie: COMB-BO8  **Läkemedel 2/Placebo** Rand. Nr: XXX  Dag: 1-7  Sponsor/prövare: Bo Söderpalm  Satsnummer: XXXXXXX |
| --- |

**Blister Vecka 2-13**

Vareniklin/Placebo

| Studie: COMB-BO8  **Läkemedel 1/Placebo** Rand. Nr: XXX  Sponsor/prövare: Bo Söderpalm  Satsnummer: XXXXXXX |
| --- |

Bupropion/Placebo

| Studie: COMB-BO8  **Läkemedel 2/Placebo** Rand. Nr: XXX  Sponsor/prövare: Bo Söderpalm  Satsnummer: XXXXXXX |
| --- |

**Kartong för Vecka 1** Upptrappning av dos

Vareniklin/Placebo

| ENDAST FÖR KLINISK LÄKEMEDELSPRÖVNING | |
| --- | --- |
| Studie: COMB-BO8  **Kit Nr 1**  11 kapslar **Läkemedel 1/Placebo** Randomiserings Nr: XXX  Dosering:  Dag 1-3: 1 kapsel dagligen på morgonen  Dag 4-7: 1 kapsel morgon och kväll  Kapslarna sväljes hela.  Blisterkartorna ska förvaras i kartongen. | Sponsor/prövare: Bo Söderpalm,  Psykiatri/SU, Blå Stråket 15, Sahlgrenska Universitetssjukhus,  Göteborg, Sverige  Telefon: 031-3424715  Förvaras utom syn- och räckhåll för barn Förvaras vid högst 25 C°  Förvaras torrt och inte i direkt solljus Satsnummer: XXXXXXX  Utgångsdatum: ÅÅÅÅ-MM |

Bupropion/Placebo

| ENDAST FÖR KLINISK LÄKEMEDELSPRÖVNING | |
| --- | --- |
| Studie: COMB-BO8 **Kit Nr: 1**  7 kapslar **Läkemedel 2/Placebo** Randomiserings Nr: XXX  Dosering: 1 kapsel dagligen på morgonen  Kapslarna sväljes hela.  Blisterkartorna ska förvaras i kartongen. | Sponsor/prövare: Bo Söderpalm,  Psykiatri/SU, Blå Stråket 15, Sahlgrenska Universitetssjukhus,  Göteborg, Sverige  Telefon: 031-3424715  Förvaras utom syn- och räckhåll för barn Förvaras vid högst 25 C°. Förvaras torrt och inte i direkt solljus Satsnummer: XXXXXXX  Utgångsdatum: ÅÅÅÅ-MM |

**Kartong för 14 dagars behandling (Vecka 2-13)**

There av 6 kits of 35 capsules for 84 days treatment. Note that 7 extra capsules will be handed out at each visit, to meet the potential need of extra capsules due to the +/-3 days accepted deviation from scheduled study visit.

**Vareniklin/Placebo**

| ENDAST FÖR KLINISK LÄKEMEDELSPRÖVNING | |
| --- | --- |
| Studie: COMB-BO8  **Kit Nr: X**  35 kapslar  **Läkemedel 1/Placebo**  Randomiserings nr: XXX  Dosering: 1 kapsel morgon och 1 kapsel kväll  Kapslarna sväljes hela.  Blisterkartorna ska förvaras i kartongen. | Sponsor/prövare: Bo Söderpalm,  Psykiatri/SU, Blå Stråket 15, Sahlgrenska Universitetssjukhus,  Göteborg, Sverige  Telefon: 031-3424715  Förvaras utom syn- och räckhåll för barn Förvaras vid högst 25 C°. Förvaras torrt och inte i direkt solljus Satsnummer: XXXXXXX  Utgångsdatum: ÅÅÅÅ-MM |

X= Kit 2, 3, 4, 5, 6, 7

**Bupropion/Placebo**

| ENDAST FÖR KLINISK LÄKEMEDELSPRÖVNING | |
| --- | --- |
| Studie: COMB-BO8  **Kit Nr: X**  35 kapslar **Läkemedel 2/Placebo**  Randomiserings nr: XXX Dosering: 1 kapsel morgon och 1 kapsel kväll  Kapslarna sväljes hela.  Blisterkartorna ska förvaras i kartongen. | Sponsor/prövare: Bo Söderpalm,  Psykiatri/SU, Blå Stråket 15, Sahlgrenska Universitetssjukhus,  Göteborg, Sverige  Telefon: 031-3424715  Förvaras utom syn- och räckhåll för barn Förvaras vid högst 25 C°. Förvaras torrt och inte i direkt solljus Satsnummer: XXXXXXX  Utgångsdatum: ÅÅÅÅ-MM |

X= Kit 2, 3, 4, 5, 6, 7, 8

**APPENDIX 2**

**Patient Emergency Card**

Swedish version

| **Akutkort** |
| --- |
| Namn: __________________________  Personnummer:___________________  Deltar i en klinisk prövning med protokoll No: EudraCT 2018-000048-24  Medicinisk ansvarig/Ansvarig forskare: Bo Söderpalm, Prof ÖL, SU/Sahlgrenska  Titel: *Påverkar vareniklin och bupropion alkoholkonsumtionen hos*  *alkoholberoende individer?*  Behandling: vareniklin 2 mg och/eller bupropion 300 mg och/eller placebo  **Randomiseringsnummer: ___________**  Studiecenter X: Telefon dagtid: XXXXXXXXX  **Vid allvarliga akutfall:**  Jourtelefon 24 tim/dygn : Telefon: xxx-xxxxxx |

English version (if traveling abroad)

| **Emergency Card** |
| --- |
| Name:____________________________  Personal ID No:_____________________  is participating in a clinical trial with protocol  No: EudraCT 2018-000048-24  Prinicpal Investigator: Bo Söderpalm, Prof ÖL, SU/Sahlgrenska  Title: *Does varenicline and bupropion affect alcohol consumption*  *in alcohol dependent individuals?*  Treatment: Varenicline 2 mg and/or bupropion 300 mg and/or placebo  Randomization code:________________________  Phone daytime to study site: XXXXXXXX  **In case of an emergency:**  Telephone number 24 hours / day: Phone: xxx-xxxxxx |
